# Deep red photocatalysis *via* direct S_0_ → T_1_ excitation of an Ir(iii) complex using 740 nm light

**DOI:** 10.1039/d6sc01462c

**Published:** 2026-04-17

**Authors:** Robert J. Ortiz, Dion B. Nemez, Mahtasim Bhuiyan, Keighlynn A. Veilleux, David E. Herbert

**Affiliations:** a Department of Chemistry and the Manitoba Institute for Materials, University of Manitoba 144 Dysart Road Winnipeg MB R3T 2N2 Canada david.herbert@umanitoba.ca

## Abstract

We report the first examples of deep-red Ir(iii) photochemistry using [Ir(ppy)_2_(p-biphe)]PF_6_ (ppy = 2-phenylpyridine; p-biphe = 6,6′,7,7′-biphenanthridine). Red light (740 nm) directly excites into the triplet manifold, populating a long-lived excited state (54 ± 3 ns) with mixed triplet metal-to-ligand charge-transfer/ligand-centered character capable of energy-transfer and electron-transfer photocatalysis.

## Abstract 2

The first examples of deep-red Ir(iii)-mediated photochemistry are reported, *via* direct excitation into the triplet manifold of an iridium coordination complex of a benzannulated biphenanthridine ligand scaffold using 740 nm light.

## Graphical abstract

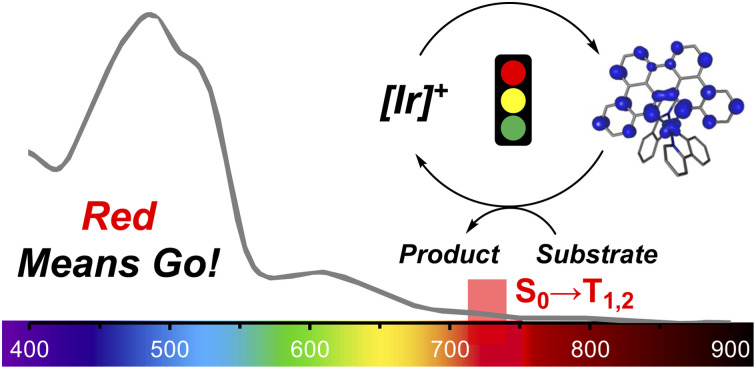

While photocatalysis is by now an established pillar of modern synthetic chemistry,^1^ the majority of reactions utilize higher energy visible light (∼390–550 nm).^2^ Longer wavelengths, notably red (600–700 nm) and near infrared (NIR) light, can penetrate deeper into solutions compared with shorter wavelengths, decreasing the chance of side reactions involving direct absorption by substrates/products, and lowering the probability of catalyst degradation, alongside the safety benefits of utilizing lower energy radiation.^3^ In addition to metalloporphyrins and related molecules, and emerging work with abundant-element chromophores,^4^ coordination complexes of osmium(ii) and ruthenium(ii) have arguably received the most attention in this area (Fig. 1).^5^ In these latter cases, the photophysics exploits the ability of heavier elements to undergo direct, spin-forbidden S_0_ → T_1_ excitation thanks to large spin–orbit coupling (SOC).^6^ Despite this success with heavier group 8 elements^7^ and the general utility of Ir(iii) photocatalysis,^8^ red-light mediated processes using group 9 chromophores has remained elusive.^9,10^ The lowest energy excitation reached with photochemically active Ir(iii) has been orange light (590 nm).^10^ This presents an opportunity for ligand design to access deeper red excitation with Ir(iii).

**Fig. 1:**
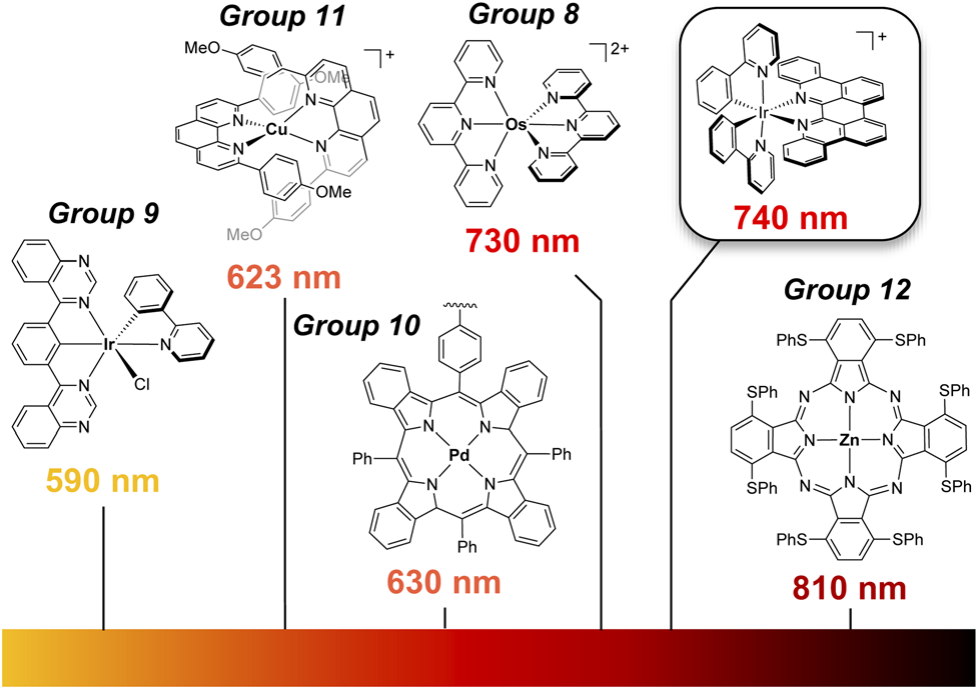
Low-energy photocatalysts in literature^4,5^ and the [Ir(ppy)_2_(p-biphe)]^+^ complex ([Ir]^+^) that is the focus of this work.

We recently reported the synthesis of [Ir(ppy)_2_(p-biphe)]PF_6_ ([Ir]PF_6_), a heteroleptic Ir(iii) complex supported by 2-phenylpyridine (ppy) and 6,6′,7,7′-biphenanthridine ligands (p-biphe).^11^ The planar, π-extended p-biphe framework^12^ allows [Ir]^+^ to absorb throughout the visible region of the electromagnetic spectrum past 800 nm and emit in the NIR. The broad absorption intrigued us with respect to the possibility of leveraging low-energy light in photocatalysis.

In particular, a weak band (*ε* ∼220 M^−1^ cm^−1^) is evident in the absorption spectrum of [Ir]^+^, assigned to direct S_0_ → T_*n*_ transitions. Exploiting this absorption using 740 nm red LEDs would represent a 150 nm (3400 cm^−1^) bathochromic shift compared to state-of-the-art low-energy Ir photocatalysts.^9,10^ The UV-Vis spectrum of a concentrated sample of [Ir]PF_6_ (0.8 mM) clearly resolves three peaks in the lowest energy region (Fig. 2a) which can be fit by Gaussian deconvolution (Fig. S1).^13^ Density functional theory (DFT) and time-dependent DFT simulations including SOC assigns these as spin-forbidden S_0_ → T_1_ (783 nm) and S_0_ → T_2_ (717 nm) transitions, overlapping with the spin-allowed S_0_ → S_1_ (605 nm; Fig. S2 and Table S1). Electron–hole density maps indicate the lowest energy S_0_ → T_1_ band has mixed metal-to-ligand charge-transfer/ligand centered (^3^MLCT/^3^LC) character, while the higher energy S_0_ → T_2_ and S_0_ → S_1_ bands present MLCT/inter-ligand charge-transfer (MLCT/ILCT) character. These bands should be directly accessible using red LEDs (red spectrum in Fig. 2a).

**Fig. 2:**
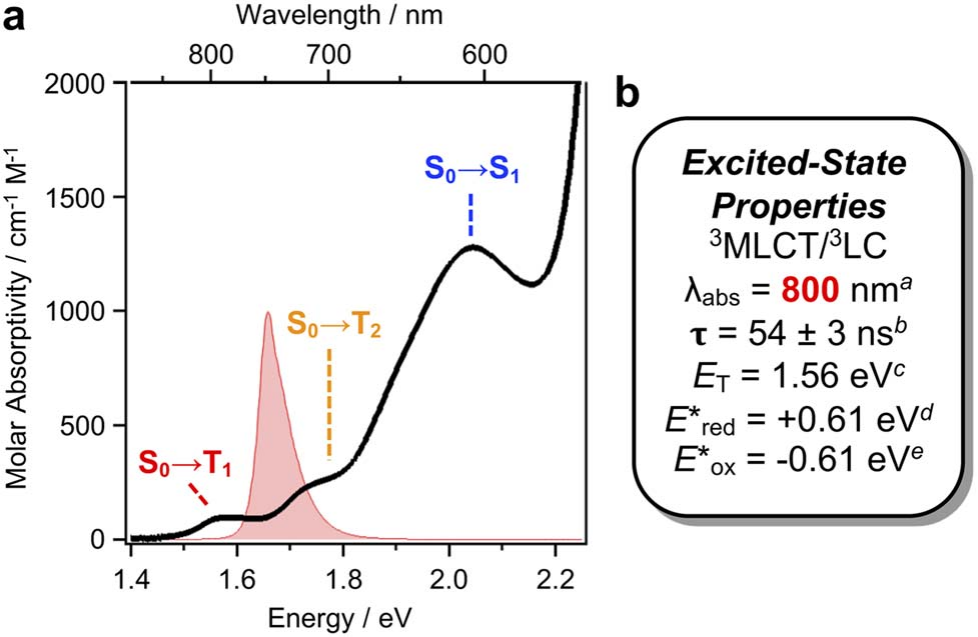
(a) The low-energy absorption spectrum of [Ir]PF_6_ with assigned transitions based on computational analysis, overlaid with the emission profile of the red LED light source used for photocatalysis. (b) Excited state properties: ^*a*^ S_0_ → T_1_ transition; ^*b*^ from oTA spectroscopy; ^*c*^ from highest energy vibrational band in the emission spectrum at 77 K; ^*d*^
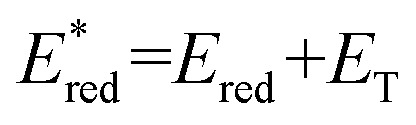
; ^*e*^
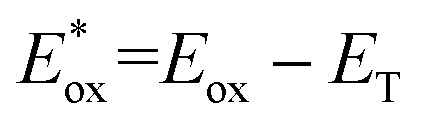
, *vs.* FcH^0/+^.

Phosphorescence from [Ir]PF_6_ (*λ*_em_ = 812 nm; *Φ*_lum_ = 0.26%) is sufficiently bright to record the lifetime of the emissive state (*τ* = 55 ns).^11^ Optical transient absorption (oTA) spectroscopy measurements of [Ir]PF_6_ in acetonitrile using a 540 nm pump confirm formation of a single, dominant excited state. The spectrum taken at a 13 ns delay shows excited-state absorptions (ESA) between 350–470 nm and 540–700 nm sandwiching a shallow bleach (480–530 nm; Fig. 3a). A linear combination of the absorption spectra of oxidized/reduced [Ir]PF_6_ measured in a spectroelectrochemical cell (Fig. S3 and S4) shows good but not perfect agreement with these features, supporting an interconfigurational ^3^LC/^3^MLCT assignment with characteristics of the MLCT state (Fig. 3b). Single-wavelength kinetic traces monitored at 600 nm can be fit to a single-exponential decay with a time constant of 54 ± 3 ns (Fig. 3c), in excellent agreement with the time constant extracted from luminescence decay measurements in dichloromethane.^11^ Overall, the 54 ns lifetime measured for the excited state of [Ir]^+^ should be more than sufficient for bimolecular quenching.

**Fig. 3:**
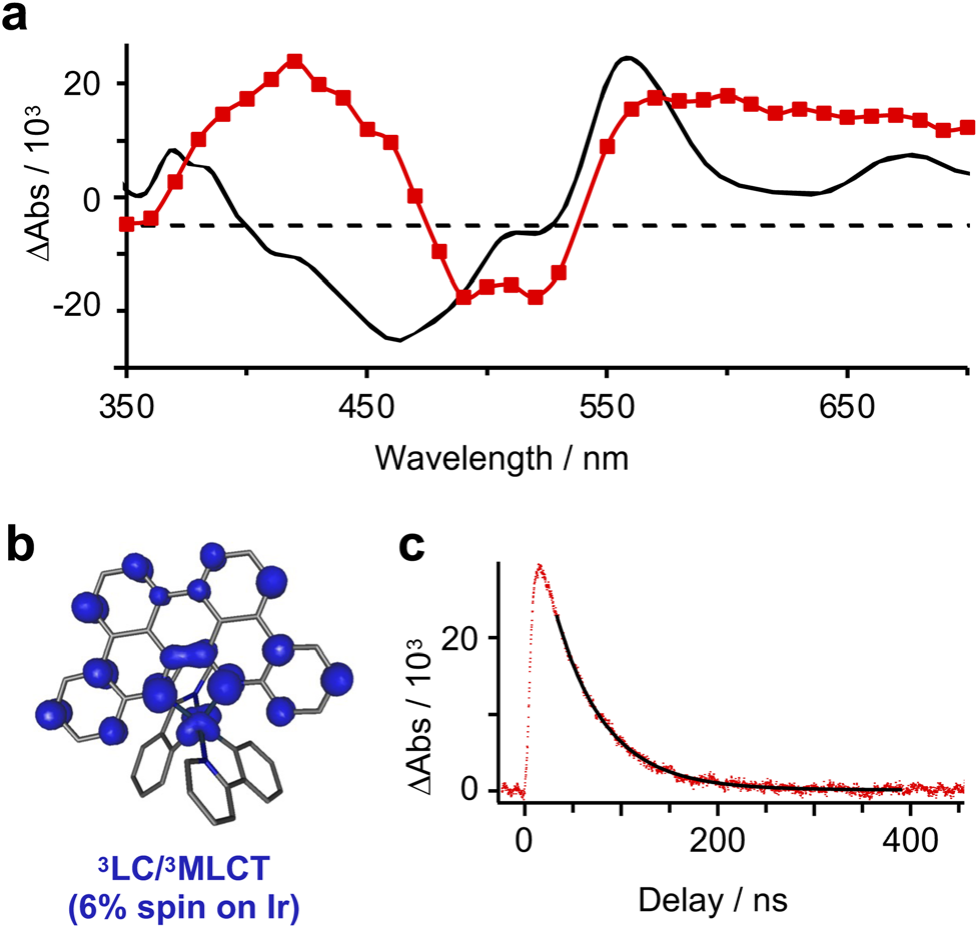
(a) Full-spectrum optical transient absorption spectroscopy of [Ir]PF_6_ in acetonitrile (*λ*_pump_ = 540 nm) at a 13 ns delay in red overlaid with the spectroelectrochemical simulation of the MLCT state in black. (b) Spin-density map of the optimized triplet state of [Ir]^+^. (c) Kinetic trace from oTA experiments monitored at 600 nm.

Cyclic voltammetry (CV) shows [Ir]^+^ can be reversibly reduced at −0.95 V and −1.53 V *vs.* FcH^0/+^ (FcH = ferrocene), with an irreversible oxidation at +0.95 V.^11^ The triplet state energy can be calculated at 1.56 eV using the highest energy emission band recorded at 77 K (Fig. S5).^14^ Using these data, we estimate excited-state redox potentials of 
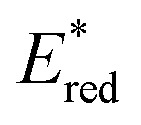
 = +0.61 V, and 
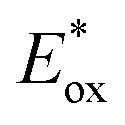
 = −0.61 V *vs.* FcH^0/+^.^15^ [Ir]^+^ thus could conceivably serve both as a strong photo-oxidant and photo-reductant.^16^ Given the irreversible nature of the oxidation event recorded by CV, we chose to target reactions in which the substrate is oxidized, as well as energy-transfer photocatalysis. This choice was bolstered by the observation that known low-energy Ir(iii) photocatalysts are generally reducing,^10^ differentiating the photochemical reactivity possible with [Ir]PF_6_. Important excited-state parameters are summarized in Fig. 2b.

First, we attempted the aerobic hydroxylation of 4-methoxyphenylboronic acid (Fig. 4a).^17^ Irradiation of [Ir]^+^ in the presence of *N*,*N*-diisopropylethylamine (DIPEA; *E*_ox_(DIPEA/DIPEA^+^) = +0.28 V *vs.* FcH^0/+^)^18,19^ leads to photooxidation of DIPEA and reduction of [Ir]^+^ (
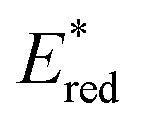
 = +0.61 V). Electron-transfer from photoreduced [Ir] to O_2_ in oxygenated CD_3_CN then facilitates boronic acid hydroxylation *via* the superoxide radical.^17^^1^H NMR spectroscopy shows 85 ± 11% conversion to the phenol in 20 h using 2 mol% [Ir]^+^ and 740 nm illumination (Fig. S6, S7 and Table S2). No conversion occurs without light, and only minor conversion is evident in the absence of [Ir]^+^ (Fig. S8, S9 and Table S2). Energy transfer mediated by ^1^O_2_ generation and reaction with furfural in alcoholic solution (Fig. 4b) is similarly effective. Using 1 mol% [Ir]^+^ excited at 740 nm in oxygenated CD_3_OD leads to 85 ± 9% conversion to 5-methoxyfuran-2-one over 23 h.^20^ When additional oxygen is bubbled in, the reaction progresses further, reaching 92 ± 4% conversion after another 3 h (Fig. S10, S11 and Table S3). A volatile side product (formic acid^21^) is evident in the ^1^H NMR spectrum and can be removed under reduced pressure (Fig. S12; see Fig. S45 for mechanism). Control reactions again confirmed the necessity of both [Ir]^+^ and light (Fig. S13, S14 and Table S3). These examples are the lowest energy photocatalysis using an Ir(iii) photocatalyst reported to date.

**Fig. 4:**
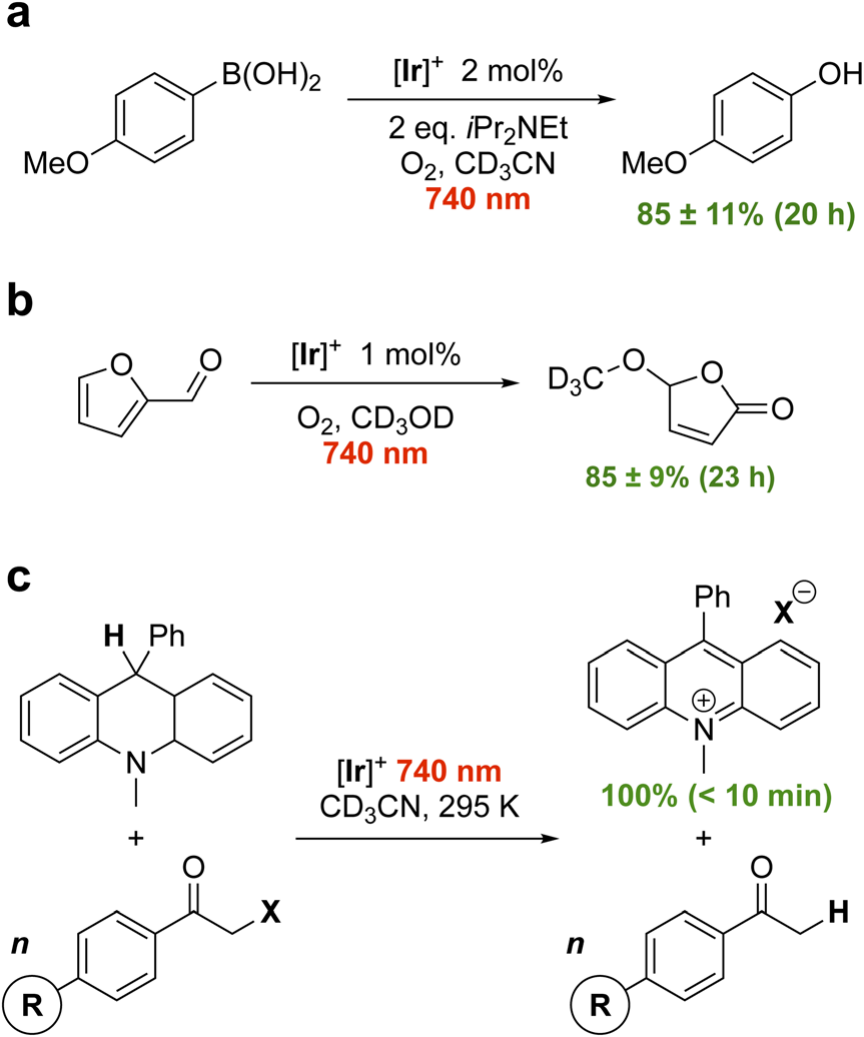
Proof-of-principle photocatalytic reactions (a–c) using [Ir]^+^ and red light (740 nm) illumination.

We next turned our attention to the thermodynamically challenging hydrodehalogenation of phenacyl halides using 9,10-dihydro-10-methylacridine as a hydride source (DHA; *E*_ox_ = +0.4 V *vs.* FcH^0/+^).^19,22^ DHA oxidation can been performed using [Ru(bpy)_3_]^2+^ (bpy = 2,2′-bipyridine) and 450 nm light.^22,23^ Here, we find photoexcited [Ir]^+^ can smoothly oxidize the even more difficult to oxidize 9-phenyl analogue 10-methyl-9-phenyl-9,10-dihydroacridine (MPA; *E*_ox_ = +0.5 V *vs.* FcH^0/+^)^19,24^ using 740 nm red light, reducing phenacyl bromide to regenerate the starting catalyst (Fig. 4c). Given the debate in literature as to the reduction potential of phenacyl bromide,^25,26^ we measure it at *E*_red_ = −1.60 V *vs.* FcH^0/+^ (Fig. S15). In the presence of excess (3 equivalents) of phenacyl bromide, MPA is completely and cleanly consumed with only acetophenone and the acridinium bromide salt observed by ^1^H NMR within 30 min at a loading of 2 mol% [Ir]^+^ (Fig. S16–S18). Reducing the catalyst loading to 0.1 mol%, the reaction still cleanly goes to completion after 65 min (Fig. S19–S22; 1.6% min^−1^). With 2 mol% [Ir]^+^, the reaction progresses at a rate of 12.1% min^−1^ and is in fact complete after 10 min of illumination (Fig. S23 and S24). Control reactions again confirm the critical role of both [Ir]^+^ and light (Fig. S25 and S26). [Ir]^+^ proved quite durable and adding additional equivalents of MPA after the initial batch is consumed restarts catalysis with no loss in activity or selectivity (Fig. S27).

Using conditions of 2 mol% [Ir]^+^ at ambient temperature (295 K), we screened the compatibility of different functional groups using a variety of 4′-functionalized 2-bromoacetophenones (R = H, OMe, Me, F, Cl, CN). In each case, 100% conversion of MPA to the acridinium bromide is observed in the presence of excess (3 equivalents) phenacyl bromide. ^1^H NMR monitoring (Fig. S28–S33) allowed us to extract the initial rates (Fig. S34) which increase with more electron-withdrawing substituents. Comparing the rate *versus* Hammett parameters (*σ*_p_),^27^ however, yielded a poor correlation (Fig. S35). A better correlation is evident using the substrate reduction potential (Fig. 5) measured *via* electrochemistry (Fig. S36–S40). The influence of the substrate reduction potential is noticeable up to a point: 2-bromo-4′-fluoro- (*E*_red_ = −1.47 V *vs.* FcH^0/+^) and 2-bromo-4′-cyanoacetophenone (*E*_red_ = −1.24 V *vs.* FcH^0/+^) are converted at similar rates (Fig. S41). We postulate that at a certain limit the reaction becomes controlled by diffusion, rather than thermodynamics (grey highlight in Fig. 5). Exchanging acylbromide for acylchloride in the form of 2-chloroacetophenone (*E*_red_ = −1.73 V *vs.* FcH^0/+^, Fig. S42), consumption of MPA is still observed, consistent with MPA photooxidation instigating catalysis, but without clean conversion to the hydrodehalogenated product (Fig. S43), in line with the thermodynamic challenge presented by this harder-to-reduce substrate.

**Fig. 5:**
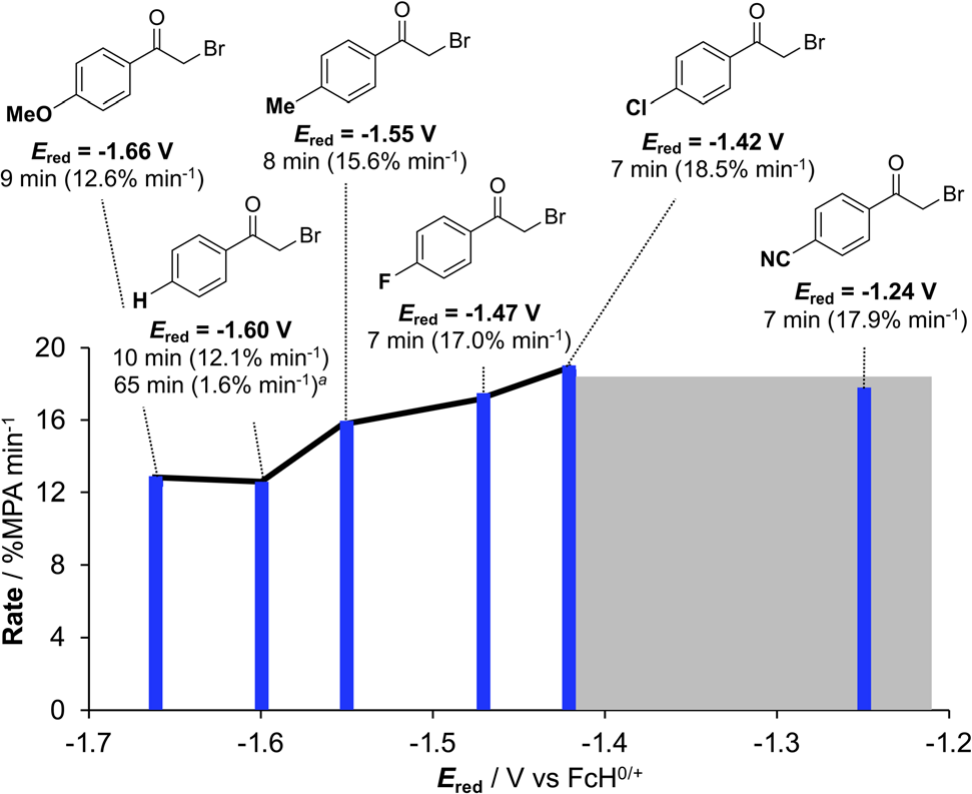
Photocatalytic hydrodehalogenation of 4-substituted phenacyl halides (2 mol% [Ir]^+^, 740 nm irradiation; ^*a*^ 0.1 mol% [Ir]^+^). Potentials are reported *versus* FcH^0/+^ along with time-to-100% consumption of MPA and the rate of consumption in parentheses (measured by ^1^H NMR). A black line to guide the eye is overlaid on the data and the diffusion-controlled potential regime beyond which the rate stabilizes is shown in grey.

## Conclusions

In summary, we report that an Ir(iii) complex of the planar, benzannulated π-extended ligand 6,6′,7,7′-biphenanthridine ([Ir]^+^)^11^ can effectively mediate photocatalysis using 740 nm deep-red light, the lowest energy illumination reported to enable such reactivity to date. This represents a 150 nm (3400 cm^−1^) bathochromic shift from the prior state-of-the-art in low-energy Ir(iii) photocatalysis^9,10^ and showcases the ability of iridium photocatalysts to reach the deep red/near-infrared region of the electromagnetic spectrum. Direct excitation of [Ir]^+^ into its triplet T_*n*_ manifold provokes population of a long-lived (55 ns) excited state with strong photoredox properties. The promise of [Ir]^+^-mediated deep red photocatalysis is demonstrated through three proof-of-principle reactions, including the aerobic hydroxylation of 4-methoxyphenylboronic acid and ^1^O_2_-mediated synthesis of 5-methoxyfuran-2-one. These reactions had not yet been reported possible *via* red-light iridium photocatalysis.^20^ In addition, we also show [Ir]^+^ to be capable of hydrodehalogenation *via* the photooxidation of MPA, a reaction that to the best of our knowledge has been previously only accessible using blue light and the easier to oxidize DHA.^23^ Efforts to expand these findings to a broader scope of reaction classes are currently underway.

## Author contributions

Conceptualization: R. J. O., D. E. H. Formal analysis: R. J. O., D. E. H. Funding acquisition: D. E. H. Investigation: R. J. O., D. B. N., M. B., K. A. V. Supervision: D. E. H. Visualization: R. J. O., D. E. H. Writing – original draft: R. J. O., D. E. H. Writing – review & editing: R. J. O., D. E. H.

## Conflicts of interest

There are no conflicts to declare.

## Supplementary Material

SC-OLF-D6SC01462C-s001

## Data Availability

The data supporting this article have been included as part of the supplementary information (SI). Supplementary information: supporting computational data, NMR spectra, electrochemical plots, time-resolved spectroscopy data, and further experimental details. See DOI: https://doi.org/10.1039/d6sc01462c.
