# Supplementary material for: Deep red photocatalysis via direct S0 → T1 excitation of an Ir(iii) complex using 740 nm light
Source: Chem Sci. 2026 Apr 17;17(23):11538–41. doi: 10.1039/d6sc01462c (PMC13133730; doi:10.1039/d6sc01462c)
Supplement: SC-017-D6SC01462C-s001 [file SC-017-D6SC01462C-s001.pdf]

Supplementary Information (SI) for Chemical Science.
This journal is © The Royal Society of Chemistry 2026

Supporting Information

for

Deep Red Photocatalysis via Direct $S_0 \rightarrow T_1$ Excitation of an Ir(III) Complex using 740 nm Light

*Robert J. Ortiz, Dion B. Nemez, Mahtasim Bhuiyan, Keighlynn A. Veilleux, David E. Herbert**

Department of Chemistry and the Manitoba Institute for Materials, University of Manitoba, 144 Dysart Road, Winnipeg, Manitoba, R3T 2N2, Canada

*david.herbert@umanitoba.ca

Table of Contents

SUPPORTING FIGURES AND TABLES 7

Figure S1. The low energy region of the absorption spectrum (black) of an 0.8 mM acetonitrile solution of [**Ir**]PF_6. A Gaussian deconvolution was performed with three components used to fit the experimental spectrum (overall fit: green dashes ---). The three fitted components are: the $S_0 \rightarrow T_1$ transition in red, the $S_0 \rightarrow T_2$ in orange, and the $S_0 \rightarrow S_1$ in blue. The wavelengths noted in the figure represent the maximum of the Gaussian peaks used in the fit. 7

Figure S2. Comparison of the experimental (—) and TD-DFT computed (---) electronic absorption spectra (including spin-orbit coupling) of [**Ir**]$^+$ in acetonitrile. Vertical energy transitions are shown in red. 8

Table S1. Spin-orbital coupling-corrected TD-DFT calculated electronic transitions along with the corresponding excitation energies, oscillator strengths (f_{OSC}), and percentages of excited states contributing to each transition for [**Ir**]$^+$ (contributions > 5%). 9

Figure S3. Spectroelectrochemical plots for [**Ir**]PF_6 in acetonitrile solution containing [$n$$Bu_4N$]$PF_6$. 9

Figure S4. Spectroelectrochemical plots for *p*-biphe in acetonitrile containing [$n$$Bu_4N$]$PF_6$ with a reducing potential applied from 0 V to -0.9 V vs Ag/AgCl. 10

Determination of Excited State Redox Potentials of [**Ir**]$^+$ 10

Figure S5. Overlay of the absorption spectrum of [**Ir**]$^+$ (black line) and emission spectra (λ_{exc} = 590 nm) in degassed CH_2Cl_2 at 295 K (red line) and butyronitrile at 77 K (blue line). Data reproduced from reference[1]. 11

Figure S6. ^{1}H NMR monitoring of the photocatalytic oxidative hydroxylation of 4-methoxyphenyl boronic acid. 12

Figure S7. Expansion of ^{1}H NMR spectra monitoring the photocatalytic oxidative hydroxylation of 4-methoxyphenyl boronic acid. 13

Table S2. Conversion of 4-methoxyphenylboronic acid to 4-methoxyphenol via photocatalysis using 740 nm irradiation and [**Ir**]$^+$ as catalyst. 13

Figure S8. Expansion of ^{1}H NMR spectra monitoring of a control oxidative hydroxylation of 4-methoxyphenyl boronic acid under 740 nm irradiation but without [**Ir**]$^+$ present. 14

Figure S9. Expansion of ^{1}H NMR spectra monitoring of the oxidative hydroxylation of 4-methoxyphenyl boronic acid without photoirradiation. 15

Figure S10. ^{1}H NMR spectra monitoring of the energy-transfer photoreactivity of furfural in oxygenated alcoholic solution. 16

Figure S11. Expansion of ^{1}H NMR spectra monitoring of the energy transfer photocatalysis of furfural in oxygenated alcoholic solution. 17

Table S3. Conversion of furfural via energy transfer photocatalysis in oxygenated alcoholic solution using 740 nm irradiation and [**Ir**]$^{+}$ as photocatalyst. 18

Figure S12. Expansion of a ^{1}H NMR spectrum of the energy transfer photocatalysis of furfural in oxygenated alcoholic solution following completion of the reaction and removal of volatiles under reduced pressure. 18

Figure S13. Expansion of ^{1}H NMR spectra monitoring of the energy transfer reaction of furfural in oxygenated alcoholic solution in the absence of [**Ir**]$^{+}$. 19

Figure S14. Expansion of ^{1}H NMR spectra monitoring the energy transfer reaction of furfural in oxygenated alcoholic solution with no photoirradiation. 20

Figure S15. Overlap of cyclic voltammogram (CV; —) and differential pulse voltammogram (DPV; ---) of 2-bromoacetophenone. 21

Figure S16. ^{1}H NMR monitoring of the photooxidation of 10-methyl-9-phenyl-9,10-dihydroacridine using [**Ir**]$^{+}$ in the presence of 2-bromoacetophenone. 22

Figure S17. ^{1}H NMR spectroscopic comparison after 30 min of 740 nm LED irradiation of 2-bromoacetophenone in the presence of [**Ir**]$^{+}$ and MPA, verifying the production of acetophenone in the products (highlighted in blue). 23

Figure S18. ^{1}H NMR spectroscopic comparison verifying the production of 9-phenyl-10-methyl-acridinium bromide (highlighted in red) from the photoreaction of MPA and 2-bromoacetophenone in the presence of [**Ir**]$^{+}$. 24

Figure S19. ^{1}H NMR spectra monitoring of the photooxidation of 10-methyl-9-phenyl-9,10-dihydroacridine in the presence of excess 2-bromoacetophenone and 0.1 mol% [**Ir**]$^{+}$. 25

Figure S20. ^{1}H NMR spectra monitoring of the photooxidation of 10-methyl-9-phenyl-9,10-dihydroacridine in the presence of excess 2-bromoacetophenone and 0.1 mol% [**Ir**]$^{+}$. 26

Figure S21. Conversion (as monitored by ^{1}H NMR) over time of 10-methyl-9-phenyl-9,10-dihydroacridine in the presence of 3 equivalents of 2-bromoacetophenone and 0.1 mol% [**Ir**]$^{+}$ under 740 nm illumination. 27

Figure S22. Expansion of the early time points of photooxidation of 10-methyl-9-phenyl-9,10-dihydroacridine in the presence of 3 equivalents of 2-bromoacetophenone and 0.1 mol% [**Ir**]$^{+}$ under 740 nm illumination. 28

Figure S23. ^{1}H NMR spectra monitoring the photooxidation of 10-methyl-9-phenyl-9,10-dihydroacridine in the presence of 3 equivalents of 2-bromoacetophenone and 2 mol% [**Ir**]$^{+}$ under 740 nm illumination 29

Figure S24. ^{1}H NMR spectra monitoring the photooxidation of 10-methyl-9-phenyl-9,10-dihydroacridine in the presence of 3 equivalents of 2-bromoacetophenone and 2 mol% [**Ir**]$^{+}$.30

Figure S25. ^{1}H NMR spectra monitoring the reaction of 10-methyl-9-phenyl-9,10-dihydroacridine in the presence of 3 equivalents of 2-bromoacetophenone and 2 mol% [**Ir**]$^{+}$ in the dark. 31

Figure S26. ^{1}H NMR spectra monitoring the photoreaction of 10-methyl-9-phenyl-9,10-dihydroacridine in the presence of 3 equivalents of 2-bromoacetophenone under 740 nm illumination but with no added [**Ir**]$^{+}$. 32

Figure S27. ^{1}H NMR spectra monitoring the photoreaction of 10-methyl-9-phenyl-9,10-dihydroacridine (MPA) in the presence of 3 equivalents of 2-bromoacetophenone and 2 mol% [**Ir**]$^{+}$ under 740 nm illumination with additional portions of MPA introduced. 33

Figure S28. ^{1}H NMR spectra monitoring the photooxidation of 10-methyl-9-phenyl-9,10-dihydroacridine in the presence of 3 equivalents of 2-bromo-4′-methoxyacetophenone and 2 mol% [**Ir**]$^{+}$ under 740 nm light. 34

Figure S29. ^{1}H NMR spectra monitoring the photooxidation of 10-methyl-9-phenyl-9,10-dihydroacridine in the presence of 3 equivalents of 2-bromo-4′-methylacetophenone, 2 mol% [**Ir**]$^{+}$ and 740 nm light. 35

Figure S30. ^{1}H NMR spectra monitoring the photooxidation of 10-methyl-9-phenyl-9,10-dihydroacridine in the presence of 3 equivalents of 2-bromo-4′-fluoroacetophenone, 2 mol% [**Ir**]$^{+}$ and 740 nm light. 36

Figure S31. ^{1}H NMR spectra monitoring the photooxidation of 10-methyl-9-phenyl-9,10-dihydroacridine in the presence of 3 equivalents of 2-bromo-4′-chloroacetophenone, 2 mol% [**Ir**]$^{+}$ and 740 nm light. 37

Figure S32. ^{1}H NMR spectra monitoring the photooxidation of 10-methyl-9-phenyl-9,10-dihydroacridine in the presence of 3 equivalents of 2-bromo-4′-cyanoacetophenone, 2 mol% [**Ir**]$^{+}$ and 740 nm light. 38

Figure S33. Comparison of the ^{1}H NMR conversion of MPA in the presence of threefold-excess of various 2-bromo-4′-functionalized acetophenones (2 mol% [**Ir**]$^{+}$, 740 nm irradiation). 39

Figure S34. Expansion of the first 5 min of the ^{1}H NMR conversion of MPA in the presence of threefold-excess of various 2-bromo-4′-functionalized acetophenones (2 mol% [**Ir**]$^{+}$, 740 nm irradiation). 40

Figure S35. Comparison of the rate of conversion of MPA in the presence of threefold-excess of various 2-bromo-4′-functionalized acetophenones (2 mol% [**Ir**]$^{+}$, 740 nm irradiation) vs. the Hammett parameter (σ_p) of the functional group on 2-bromo-4′-functionalized acetophenones. 41

Figure S36. Overlap of cyclic voltammogram (CV; —) and differential pulse voltammogram (DPV; ---) of 2-bromo-4′-methoxyacetophenone. 42

Conditions: 6 mM acetonitrile solution containing 0.1 M [nBu$_4$N]PF$_6$ electrolyte at a CV scan rate of 100 mV/s. The $E_{1/2}$ of the irreversible reduction is estimated from the DPV to be -1.55 V vs FcH$^{0/+}$. 43

Figure S38. Overlap of cyclic voltammogram (CV; —) and differential pulse voltammogram (DPV; ---) of 2-bromo-4′-fluoroacetophenone. 44

Figure S39. Overlap of cyclic voltammogram (CV; —) and differential pulse voltammogram (DPV; ---) of 2-bromo-4′-chloroacetophenone. 45

Figure S40. Overlap of cyclic voltammogram (CV; —) and differential pulse voltammogram (DPV; ---) of 2-bromo-4′-cyanoacetophenone. 46

Figure S41. Comparison of the conversion rate of MPA photooxidation versus the reduction potential (E_{red}) of 2-bromo-4′-functionalized acetophenones. 47

Figure S42. Overlap of cyclic voltammogram (CV; —) and differential pulse voltammogram (DPV; ---) of 2-chloroacetophenone. 48

Figure S43. ^{1}H NMR spectra monitoring the photoreaction of 10-methyl-9-phenyl-9,10-dihydroacridine in the presence of 3 equivalents of 2-chloroacetophenone, 2 mol % [**Ir**]$^{+}$ and 740 nm light. 49

EXPERIMENTAL DETAILS 50

UV-Vis Absorbance 50

Electrochemistry 50

Spectroelectrochemistry 51

Time-resolved Absorption Spectroscopy 51

Computational Details 52

Photocatalytic Experiments 52

Figure S44. Emission spectrum of the 740 nm LED (Hepatochem) used in the photocatalytic experiments. 53

Electron-Transfer Photocatalytic Synthesis of 4-Methoxyphenol in Oxygenated Solution 53

Energy Transfer Photocatalysis of Furfural in Oxygenated Alcoholic Solution 54

Figure S45. Proposed mechanism of furanone production from singlet oxygen-mediated reactivity of furfural in deuterated methanol.[21–24] 54

General Procedure for the Photocatalytic Oxidation of 10-Methyl-9-phenyl-9,10-dihydroacridine ____ 54

Using 0.1 mol% of Photocatalyst for the Photocatalytic Oxidation of 10-Methyl-9-phenyl-9,10-dihydroacridine ____ 55

Subsequent Additions of 10-Methyl-9-phenyl-9,10-dihydroacridine During Photocatalytic Reactions ____ 55

Figure S46. UV-Vis absorption spectra collected before and after ns oTA experiments (λ_{probe} = 540 nm) demonstrating the photostability of [**Ir**]PF_6. ____ 56

References ____ 57

SUPPORTING FIGURES AND TABLES

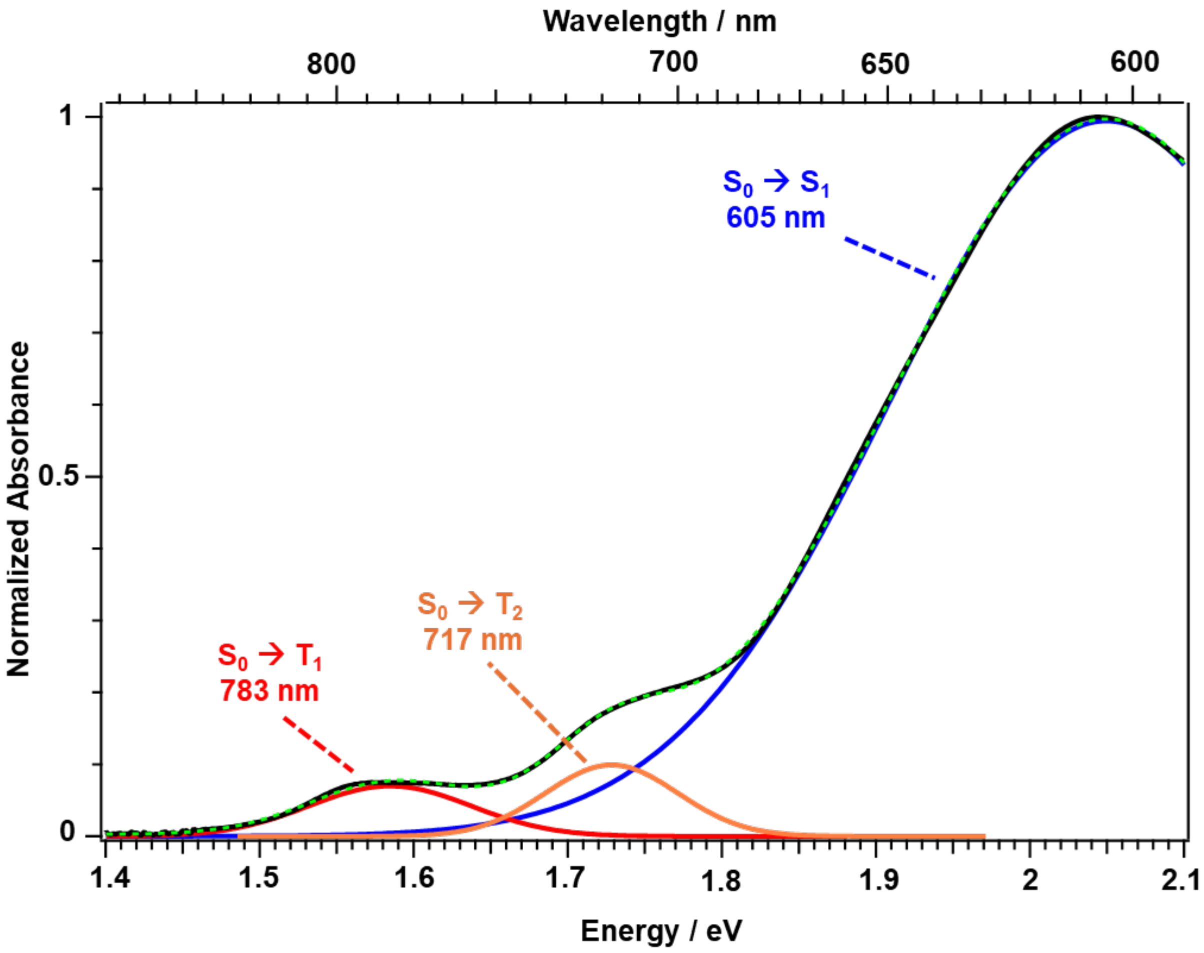

Figure S1. The low energy region of the absorption spectrum (black) of an 0.8 mM acetonitrile solution of [**Ir**]PF_6. A Gaussian deconvolution was performed with three components used to fit the experimental spectrum (overall fit: green dashes ---). The three fitted components are: the $S_0 \rightarrow T_1$ transition in red, the $S_0 \rightarrow T_2$ in orange, and the $S_0 \rightarrow S_1$ in blue. The wavelengths noted in the figure represent the maximum of the Gaussian peaks used in the fit.

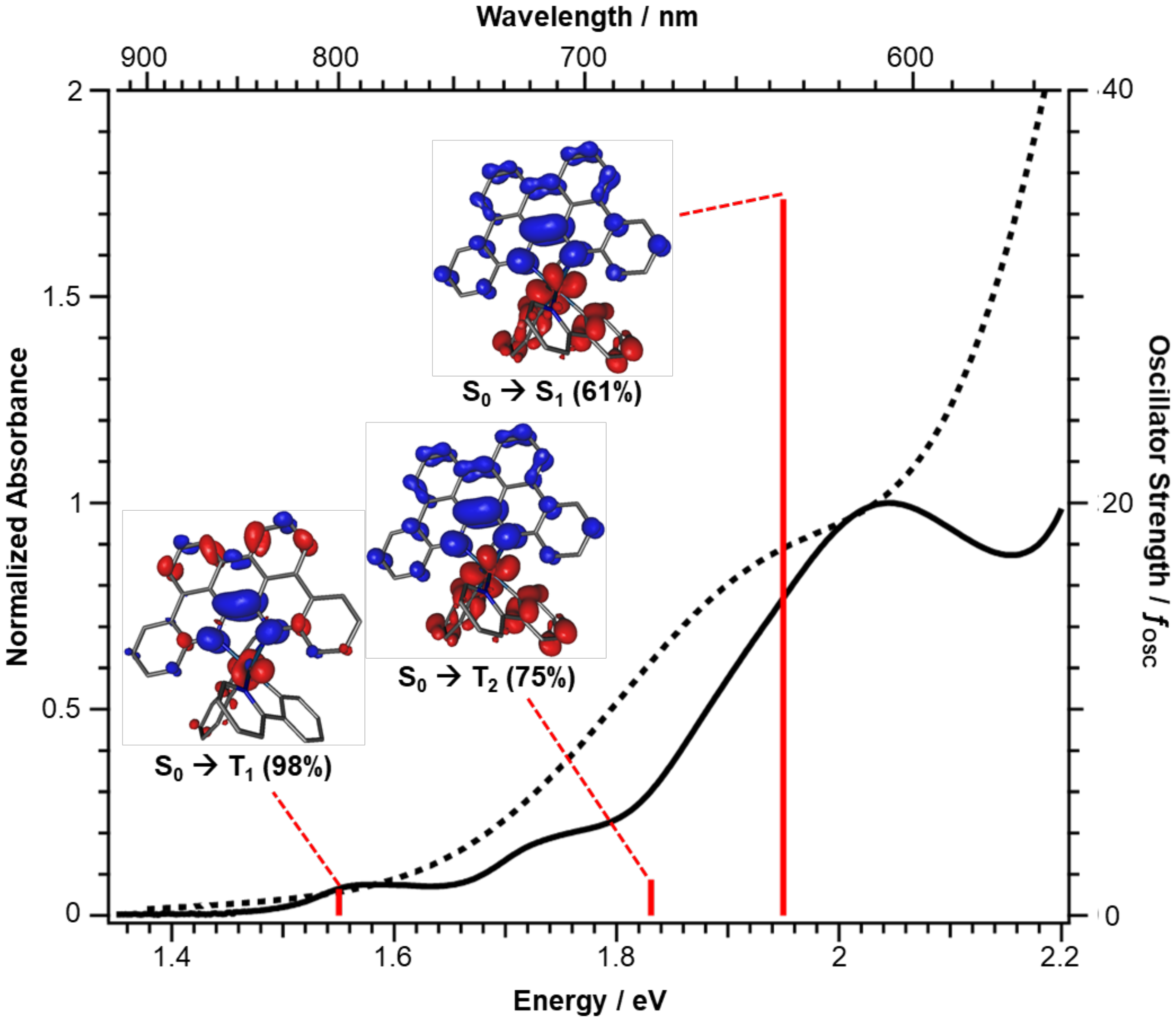

Figure S2. Comparison of the experimental (—) and TD-DFT computed (---) electronic absorption spectra (including spin-orbit coupling) of [**Ir**]$^{+}$ in acetonitrile. Vertical energy transitions are shown in red.

TD-DFT simulation parameters: FWHM = 0.35 eV; SO-RIJCOSX-ZORA-SMD-PBE0/OLD-ZORA-TZVP+SARC/J//SMD-PBE0-D3(BJ)/def2-SVP. The spectrum is normalized to the highest energy transition shown. Only transitions with oscillator strengths > 0.001 are shown. Electron-hole density maps are provided for each transition (blue = electron; red = hole; isosurface value = 0.0015). As the spectrum is spin-orbit corrected, the percentages in parentheses indicate how much of each transition is attributed the indicated state.

Table S1. Spin-orbital coupling-corrected TD-DFT calculated electronic transitions along with the corresponding excitation energies, oscillator strengths (f_{OSC}), and percentages of excited states contributing to each transition for [**Ir**]$^+$ (contributions > 5%).

No.	E / nm	E / eV	f_{OSC}	Composition
3	799.9	1.55	0.0013	**T_1 (98%)**
4	677.3	1.83	0.0017	**T_2 (75%)**, T_3 (6%), S_1 (6%), S_2 (7%),
7	635.9	1.95	0.0347	**S_1 (61%)**, T_2 (16%), T_3 (9%), T_4 (6%)

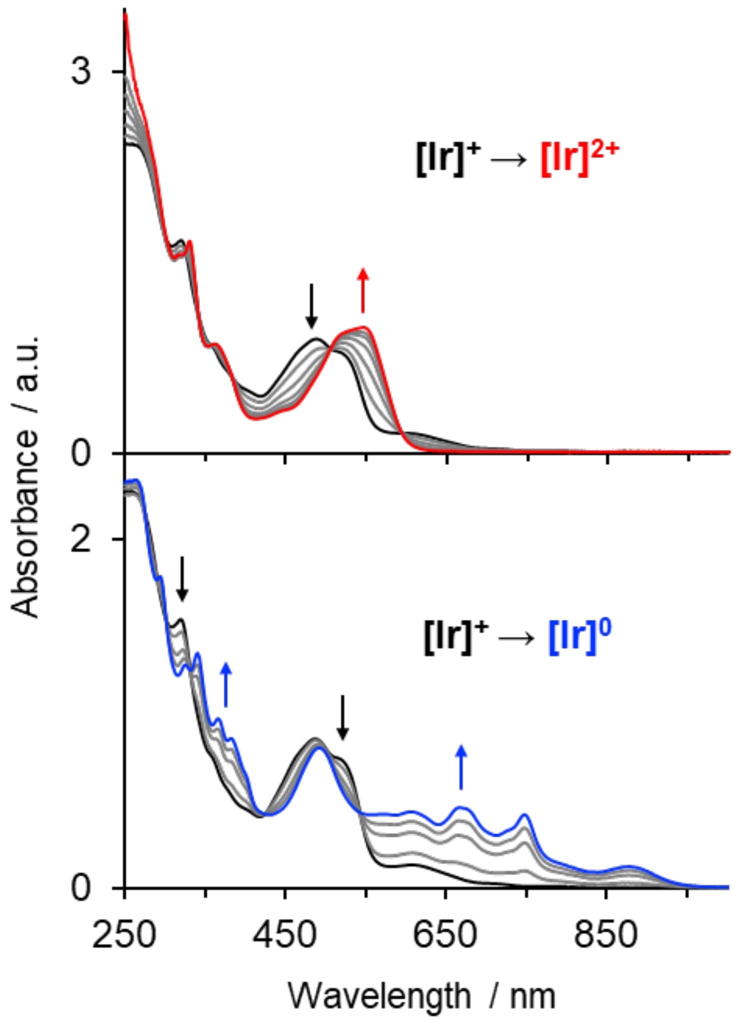

Figure S3. Spectroelectrochemical plots for [**Ir**]PF_6 in acetonitrile solution containing [$n$$Bu_4N$]$PF_6$.

Conditions: Top - an oxidizing potential was applied from 1.35 V to 1.51 V vs Ag/AgCl; Bottom - a reducing potential was applied from 0 V to -0.45 V vs Ag/AgCl. In both spectra, the black trace represents the steady-state absorption, while the coloured traces represent the final spectrum following oxidation or reduction.

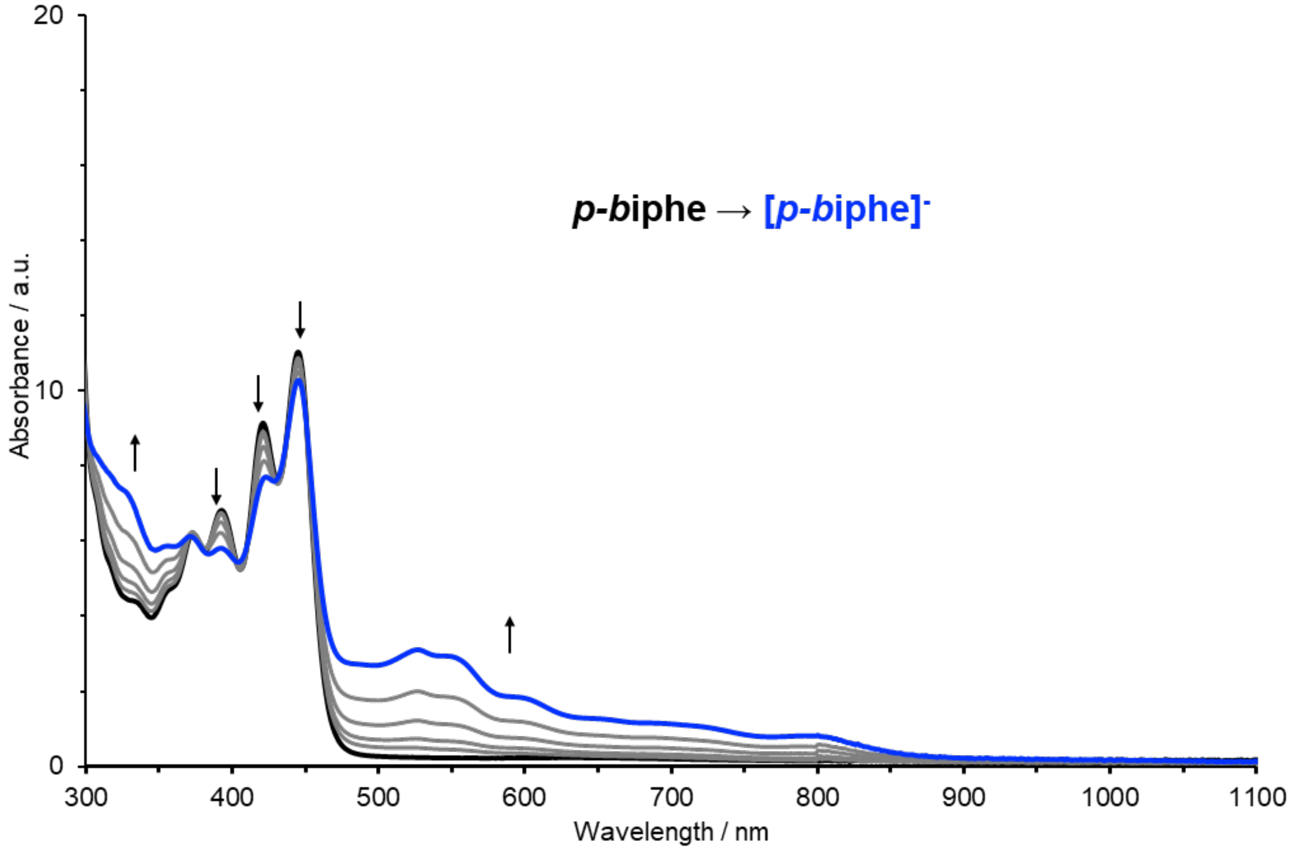

Figure S4. Spectroelectrochemical plots for *p*-biphe in acetonitrile containing [nBu$_4$N]PF$_6$ with a reducing potential applied from 0 V to -0.9 V vs Ag/AgCl.

The black trace represents the steady-state absorption, while the coloured traces represent the final spectrum following oxidation or reduction.

Determination of Excited State Redox Potentials of [Ir]$^+$

The following equations were used:

$$E^*{}_{red} = E_{red} + E_T$$
$$E^*{}_{ox} = E_{ox} - E_T$$

E_T was determined from the maximum of the emission spectrum recorded at 77 K, as the highest energy peak should represent lowest energy triplet state:

$$E_T = 1.56 \text{ eV}$$

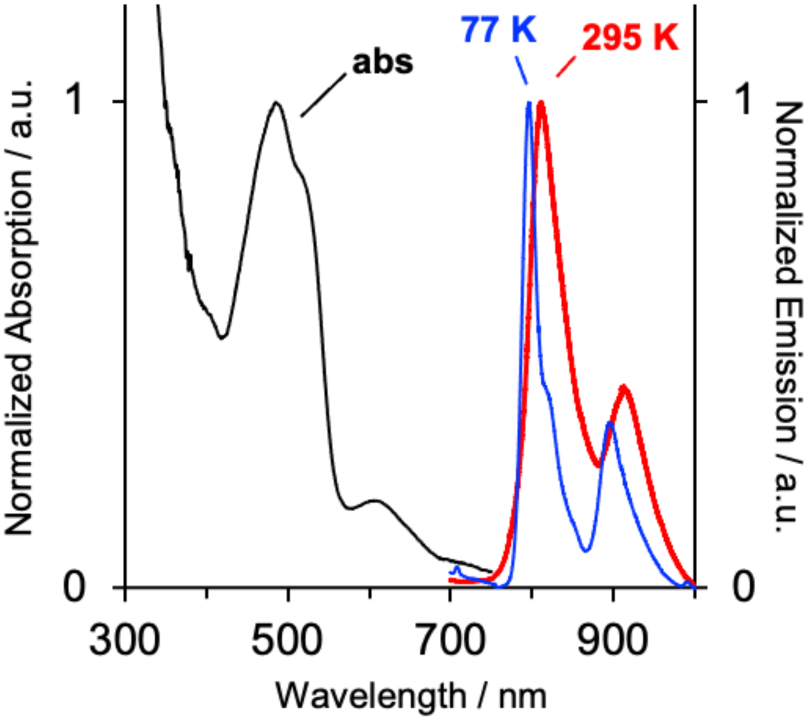

Figure S5. Overlay of the absorption spectrum of [**Ir**]$^+$ (black line) and emission spectra (λ_{exc} = 590 nm) in degassed CH_2Cl_2 at 295 K (red line) and butyronitrile at 77 K (blue line). Data reproduced from reference[1].

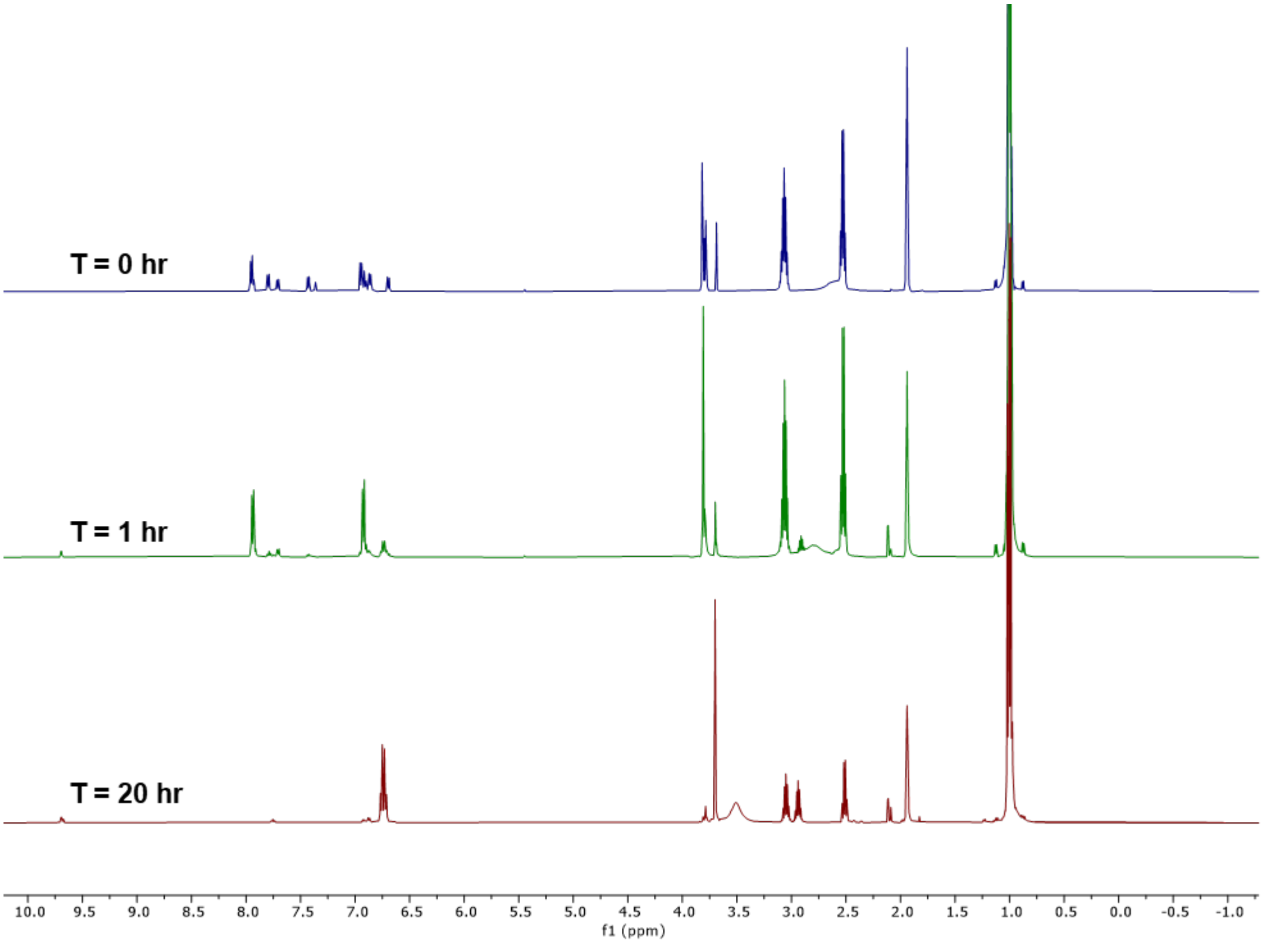

Figure S6. ^{1}H NMR monitoring of the photocatalytic oxidative hydroxylation of 4-methoxyphenyl boronic acid.

Conditions: 2 mol% loading of [**Ir**]$^+$ excited with a 740 nm LED in 0.6 mL oxygenated CD_3CN containing 2 equivalents of *N,N*-diisopropylethylamine. Product formation was confirmed via match to literature.[2]

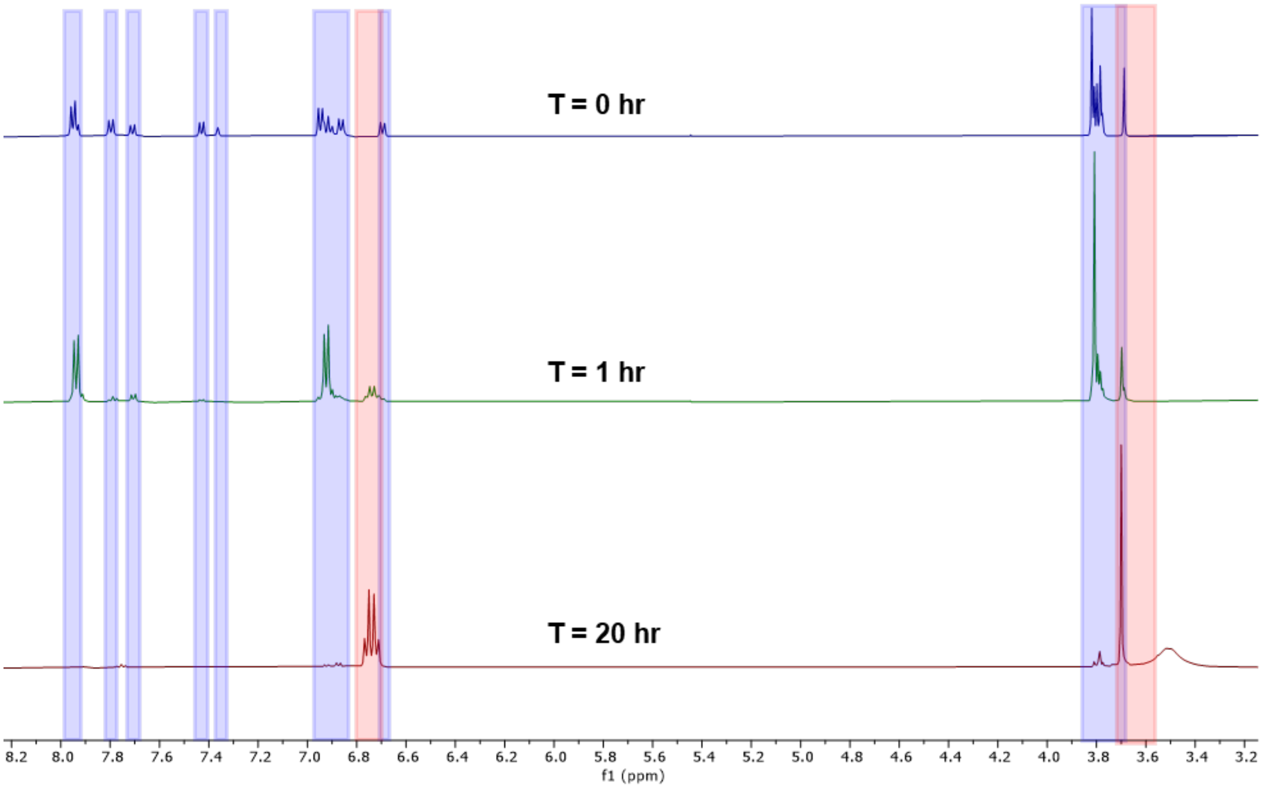

Figure S7. Expansion of ^{1}H NMR spectra monitoring the photocatalytic oxidative hydroxylation of 4-methoxyphenyl boronic acid.

The blue boxes highlight the starting material, red highlights product peaks. Product formation was confirmed via match to literature.[2]

Table S2. Conversion of 4-methoxyphenylboronic acid to 4-methoxyphenol via photocatalysis using 740 nm irradiation and [**Ir**]$^{+}$ as catalyst.

Conversion (time / hr)	No light	No Catalyst
0 % (0)	0 % (0)	0 % (0)
13 ± 1 % (1)	0 % (1)	22 % (18)
85 ± 11 % (20)	0 % (20)	27 % (24)
-	0 % (24)	-

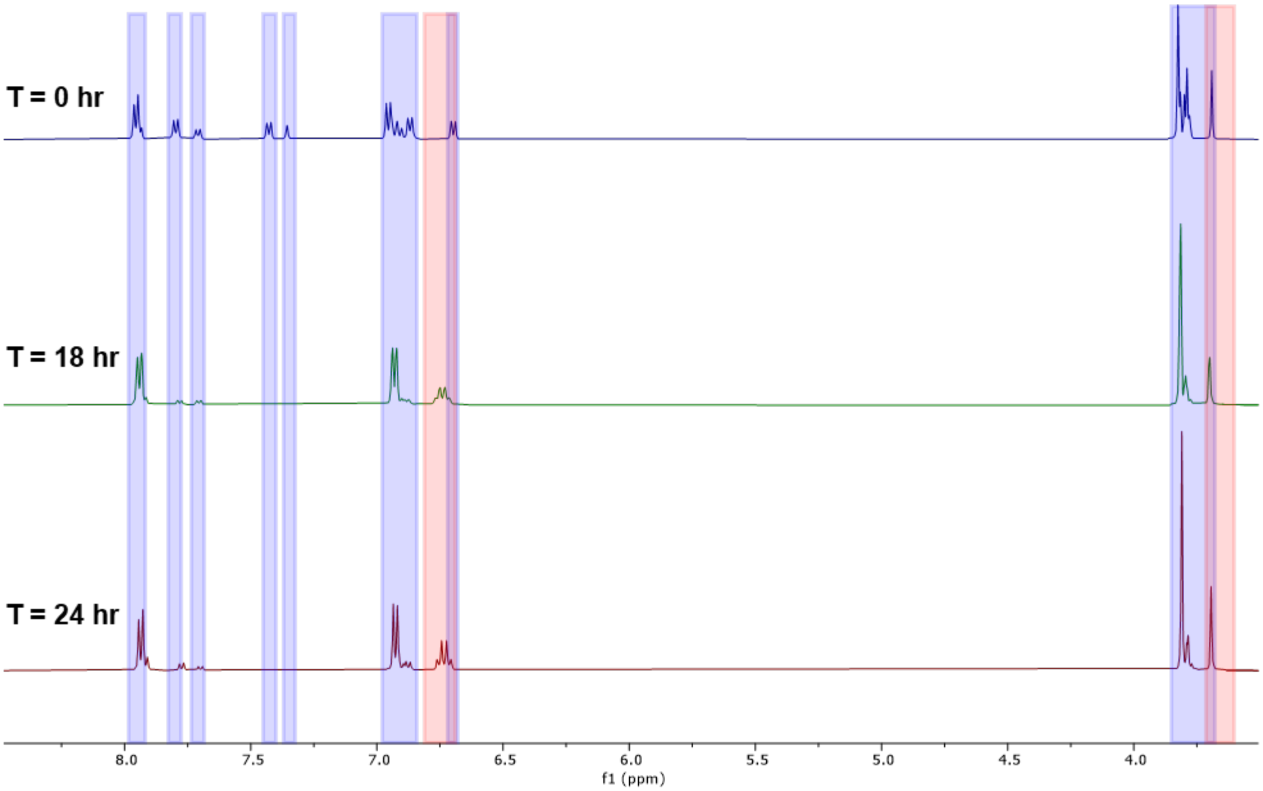

Figure S8. Expansion of ^{1}H NMR spectra monitoring of a control oxidative hydroxylation of 4-methoxyphenyl boronic acid under 740 nm irradiation but without [**Ir**]$^{+}$ present.

The blue boxes highlight the starting material, red highlights product peaks. Product formation was confirmed via match to literature.[2]

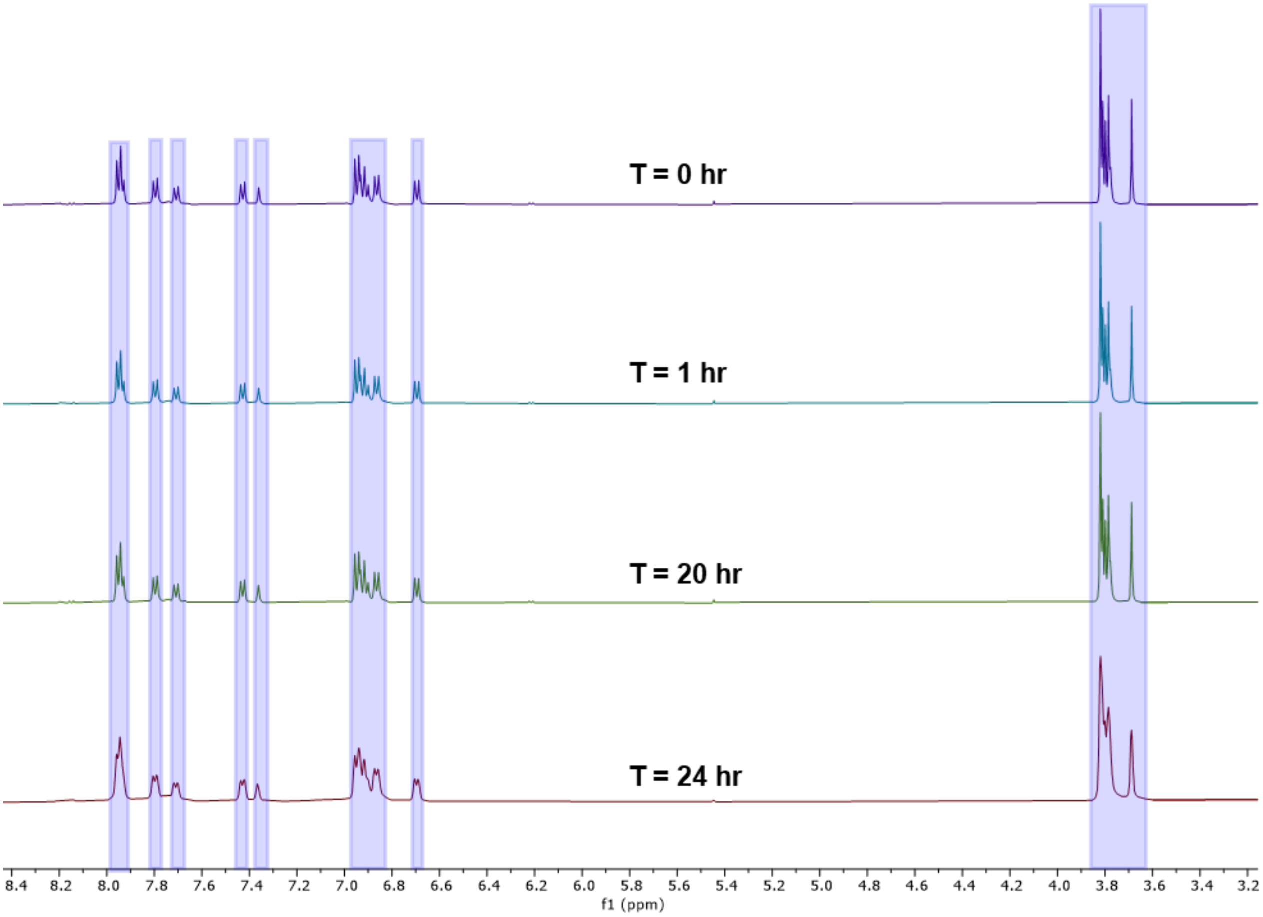

Figure S9. Expansion of ^{1}H NMR spectra monitoring of the oxidative hydroxylation of 4-methoxyphenyl boronic acid without photoirradiation.

The blue boxes highlight the starting material; no conversion was observed.

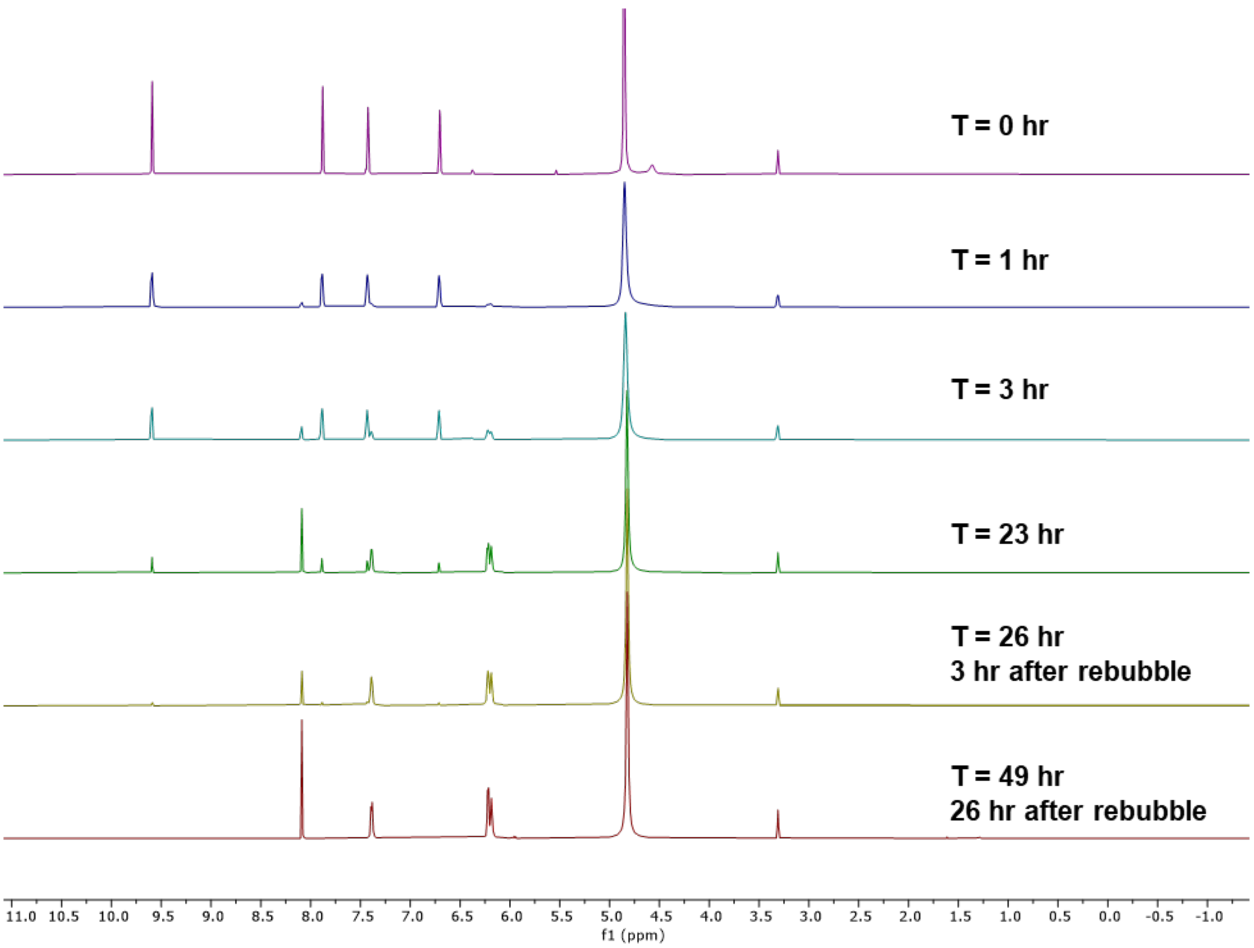

Figure S10. ^{1}H NMR spectra monitoring of the energy-transfer photoreactivity of furfural in oxygenated alcoholic solution.

Conditions: 1 mol% loading of [**Ir**]$^+$ excited using a 740 nm LED in 0.5 mL of oxygenated CD_3OD. Product formation was confirmed via match to literature.[3] Note: additional O_2 was bubbled in at the 23 h time point.

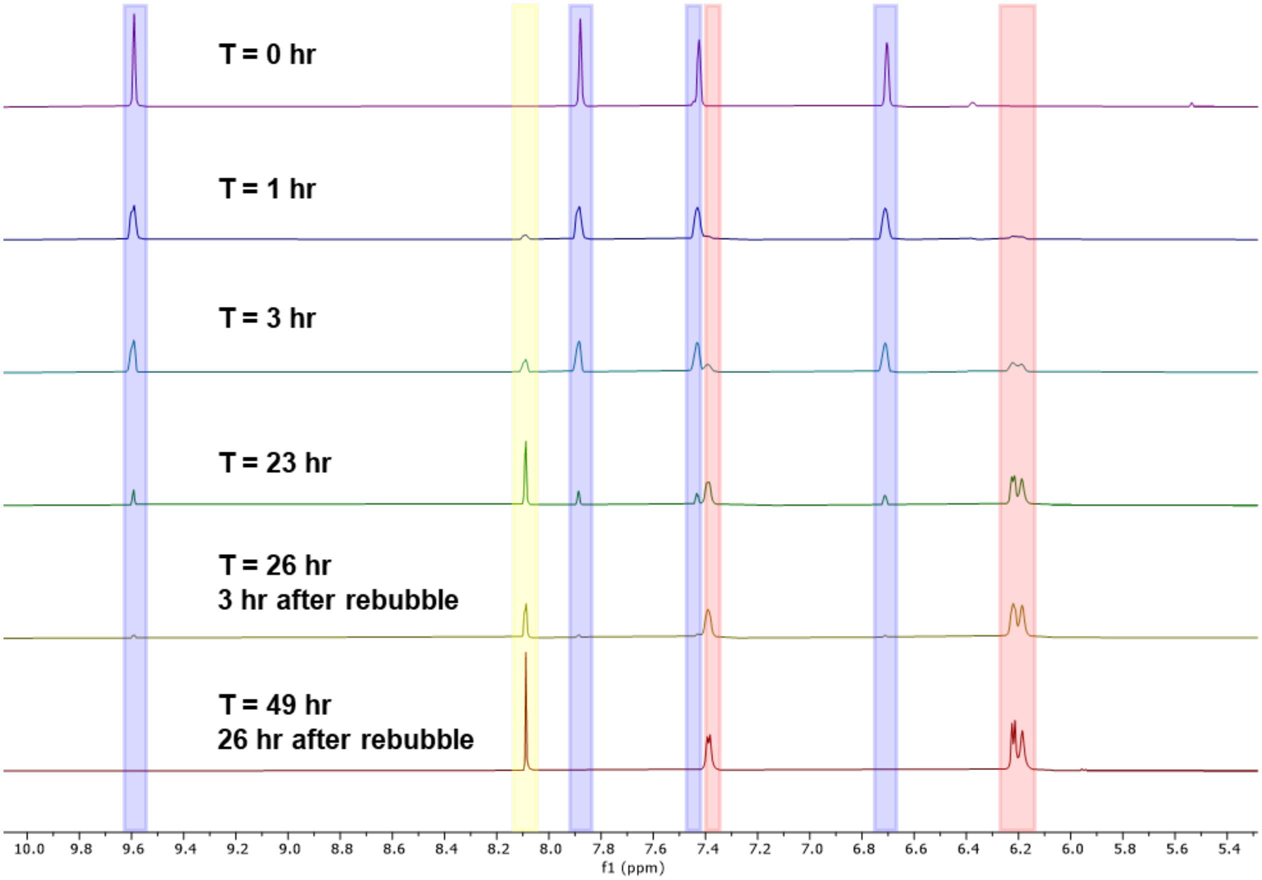

Figure S11. Expansion of ^1H NMR spectra monitoring of the energy transfer photocatalysis of furfural in oxygenated alcoholic solution.

The blue boxes highlight starting material peaks, while the red boxes highlight product peaks and yellow box highlights the formic acid byproduct. Product formation was confirmed via match to literature.[3]

Table S3. Conversion of furfural via energy transfer photocatalysis in oxygenated alcoholic solution using 740 nm irradiation and [**Ir**]$^+$ as photocatalyst.

Conversion (time / hr)	No light	No Catalyst
0 % (0)	0 % (0)	0 % (0)
28 ± 2 % (3)	0 % (1)	4 % (21)
85 ± 9 % (23)	0 % (20)	15 % (93)
92 ± 4 % (26 – *3 h after O_2 rebubbled)*[a]	0 % (24)	-
100 ± 0 % (49 – *26 h after O_2 rebubbled)*[a]	-	-

[a] For the full reaction, oxygen was reintroduced in at the 23 h mark.

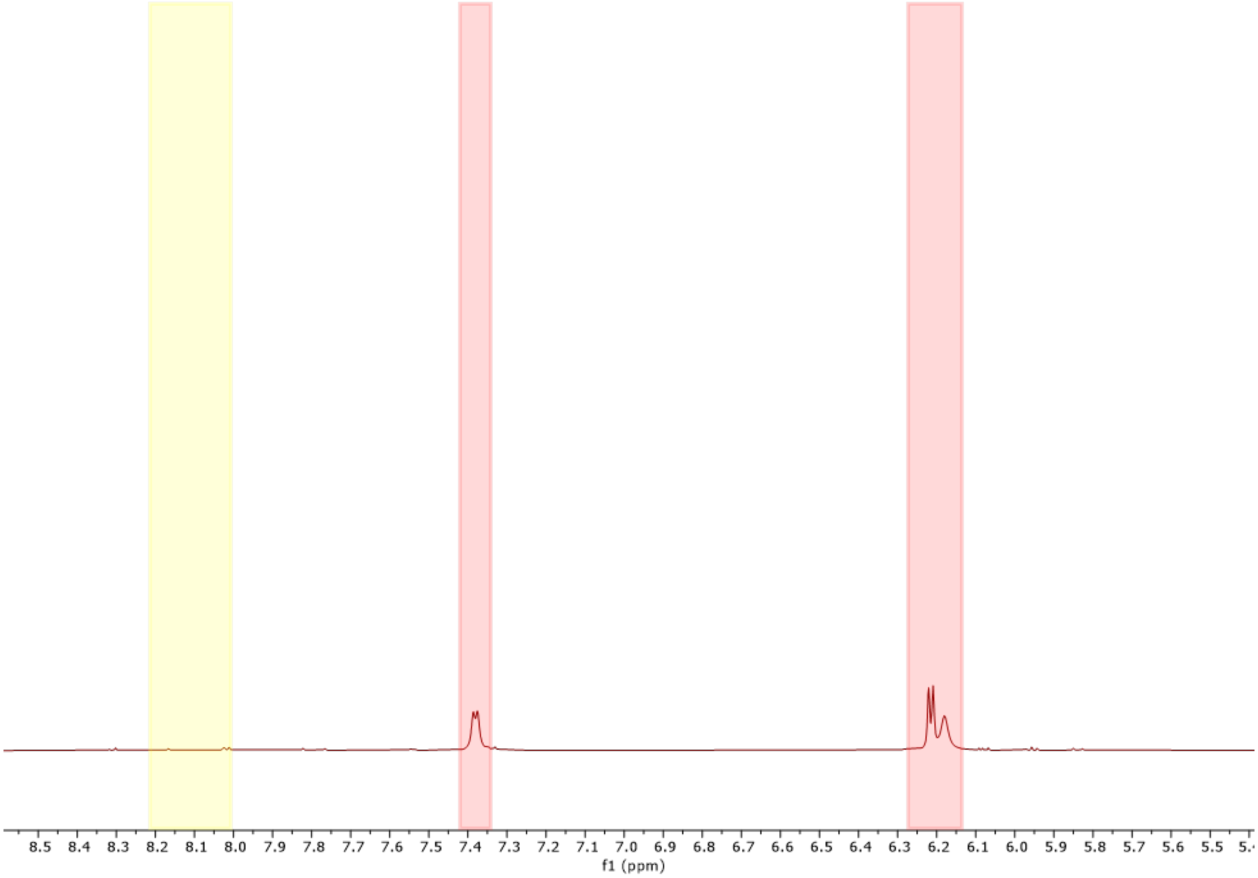

Figure S12. Expansion of a ^{1}H NMR spectrum of the energy transfer photocatalysis of furfural in oxygenated alcoholic solution following completion of the reaction and removal of volatiles under reduced pressure.

This spectrum demonstrates that the side product (formic acid) can be removed *in vacuo*. The yellow box highlights where the side product was observed (now absent) while the red boxes highlight retention of the non-volatile reaction product.

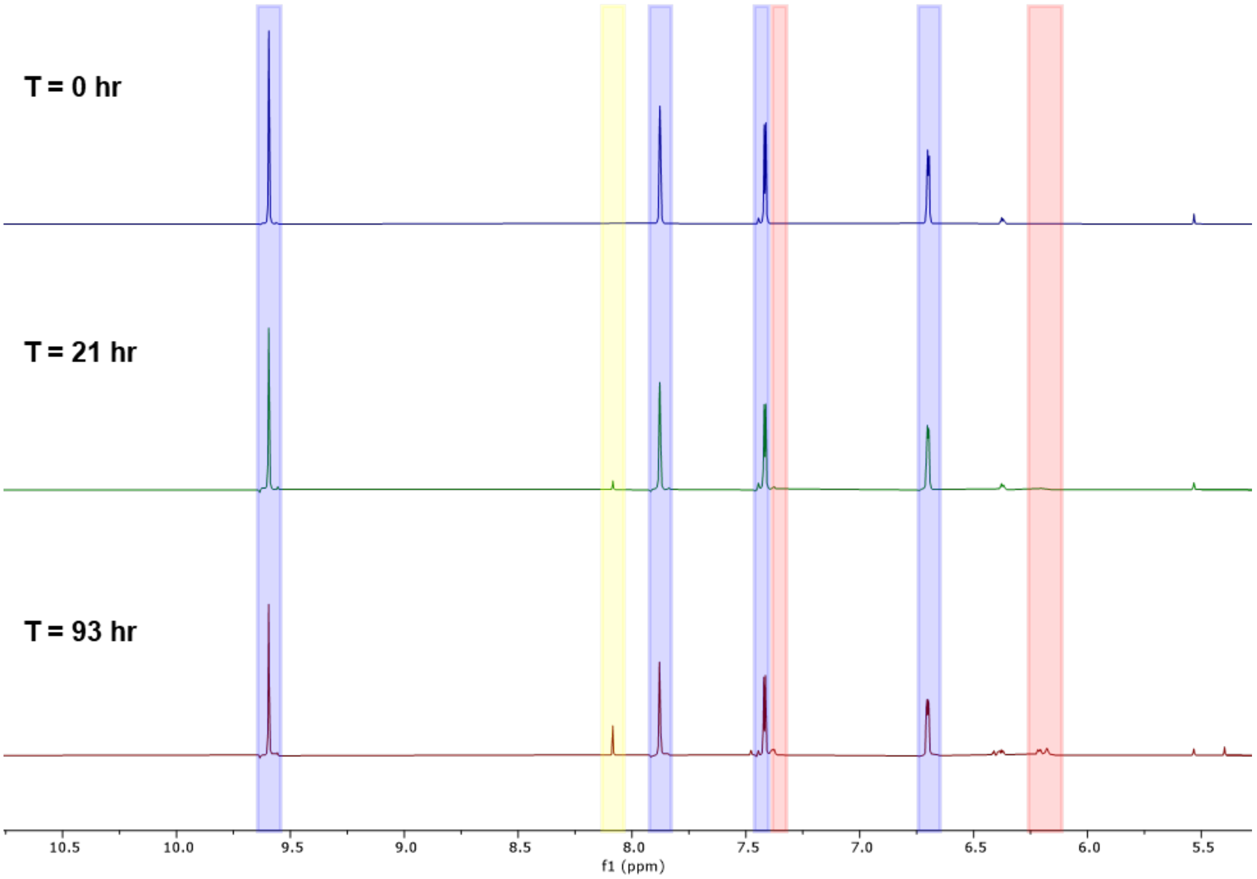

Figure S13. Expansion of ^{1}H NMR spectra monitoring of the energy transfer reaction of furfural in oxygenated alcoholic solution in the absence of [**Ir**]$^+$.

The blue boxes highlight peaks corresponding to starting material; the red boxes highlight product peaks; the yellow box highlights the formic acid side product (see Figure S12). Product formation was confirmed via match to literature.[3]

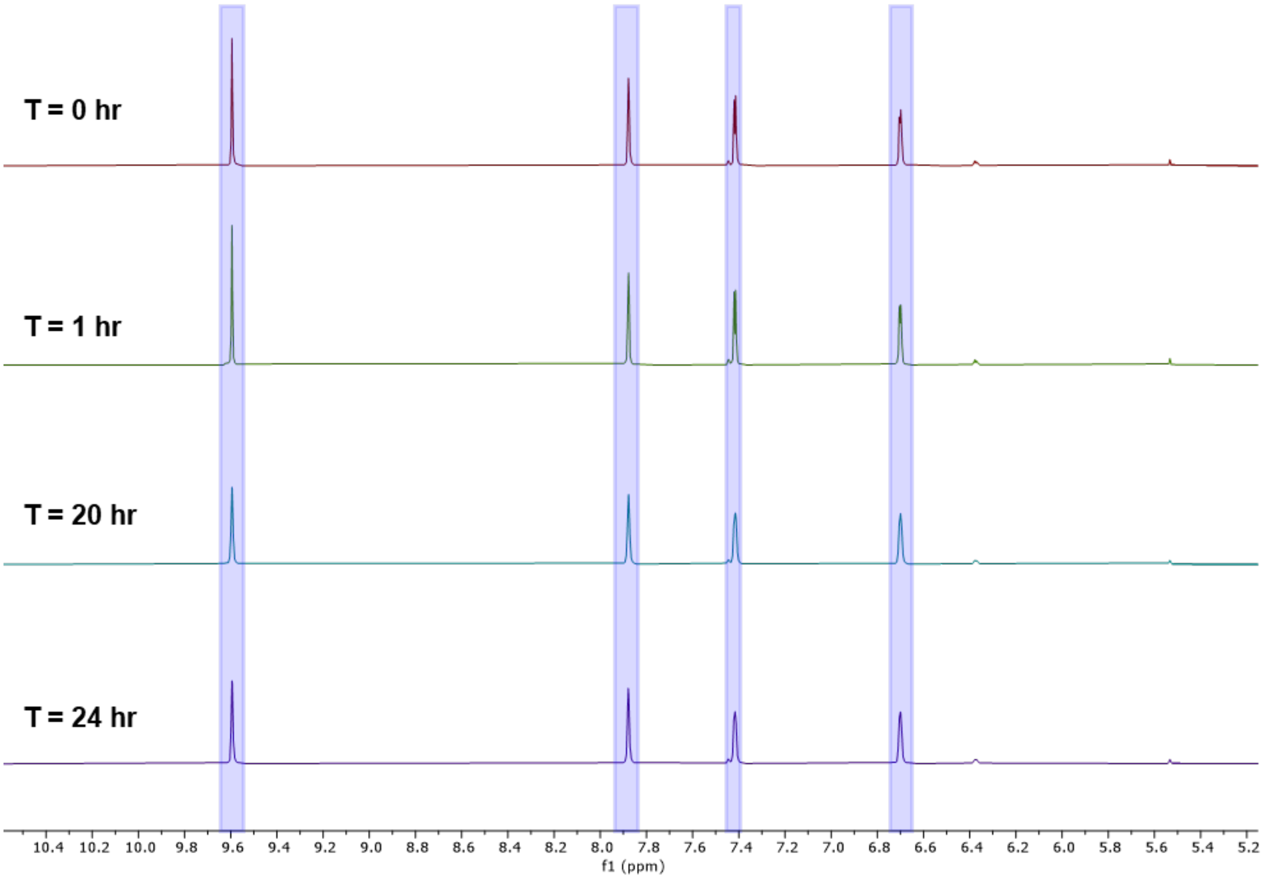

Figure S14. Expansion of ^{1}H NMR spectra monitoring the energy transfer reaction of furfural in oxygenated alcoholic solution with no photoirradiation.

The blue boxes highlight the starting material; no conversion to product is observed.

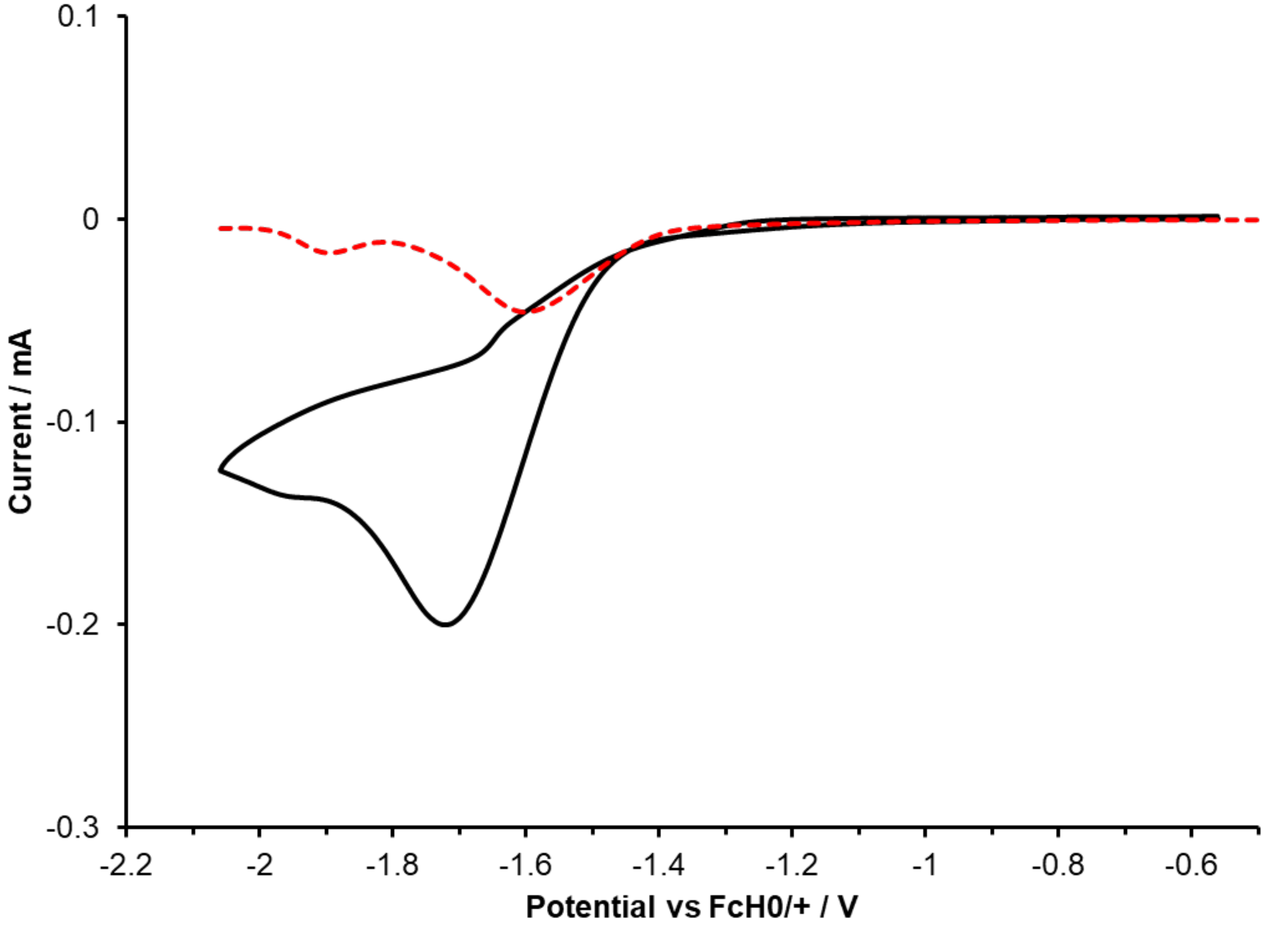

Figure S15. Overlap of cyclic voltammogram (CV; —) and differential pulse voltammogram (DPV; ---) of 2-bromoacetophenone.

Conditions: 7 mM acetonitrile solution of [**Ir**]PF_6 containing 0.1 M [$n$$Bu_4N$]$PF_6$ electrolyte; CV scan rate of 100 mV s^{-1}. The $E_{1/2}$ of the irreversible reduction is estimated from the DPV to be -1.60 V vs $FcH^{0/+}$.

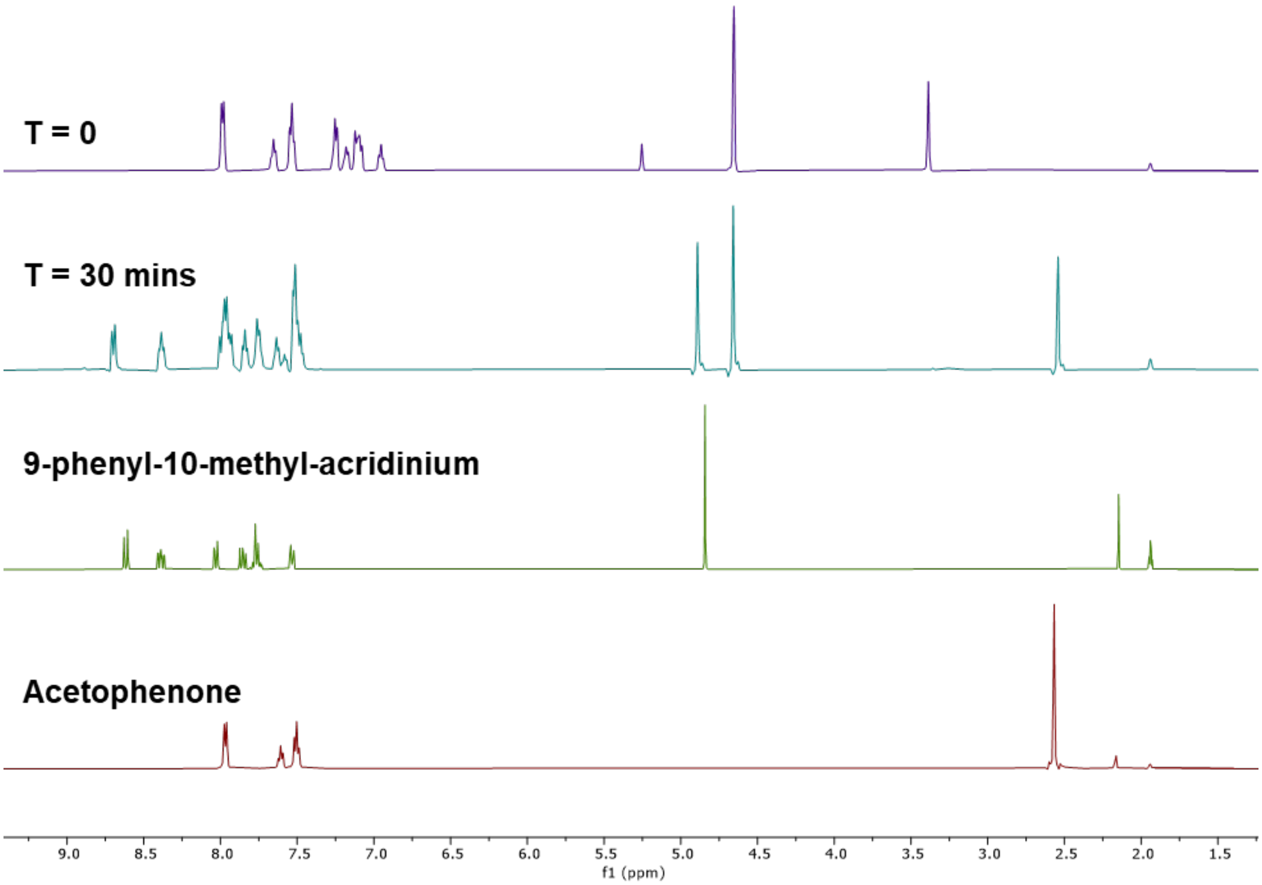

Figure S16. ^{1}H NMR monitoring of the photooxidation of 10-methyl-9-phenyl-9,10-dihydroacridine using [**Ir**]$^+$ in the presence of 2-bromoacetophenone.

Conditions: 2 mol % [**Ir**]$^+$ excited at 740 nm. Acetophenone and 9-phenyl-10-methyl-acridinium bromide are the sole products.

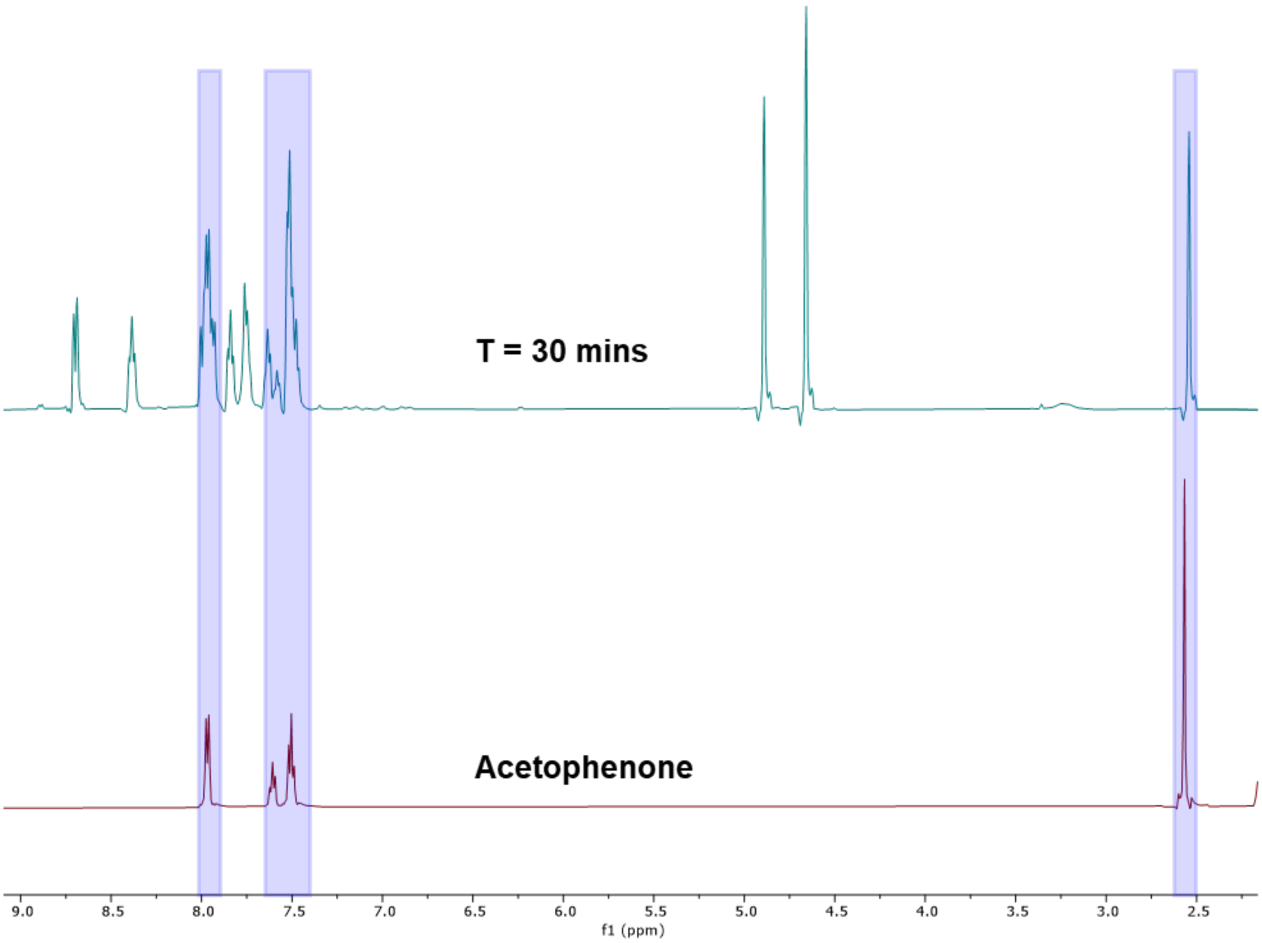

Figure S17. ^{1}H NMR spectroscopic comparison after 30 min of 740 nm LED irradiation of 2-bromoacetophenone in the presence of [**Ir**]$^{+}$ and MPA, verifying the production of acetophenone in the products (highlighted in blue).

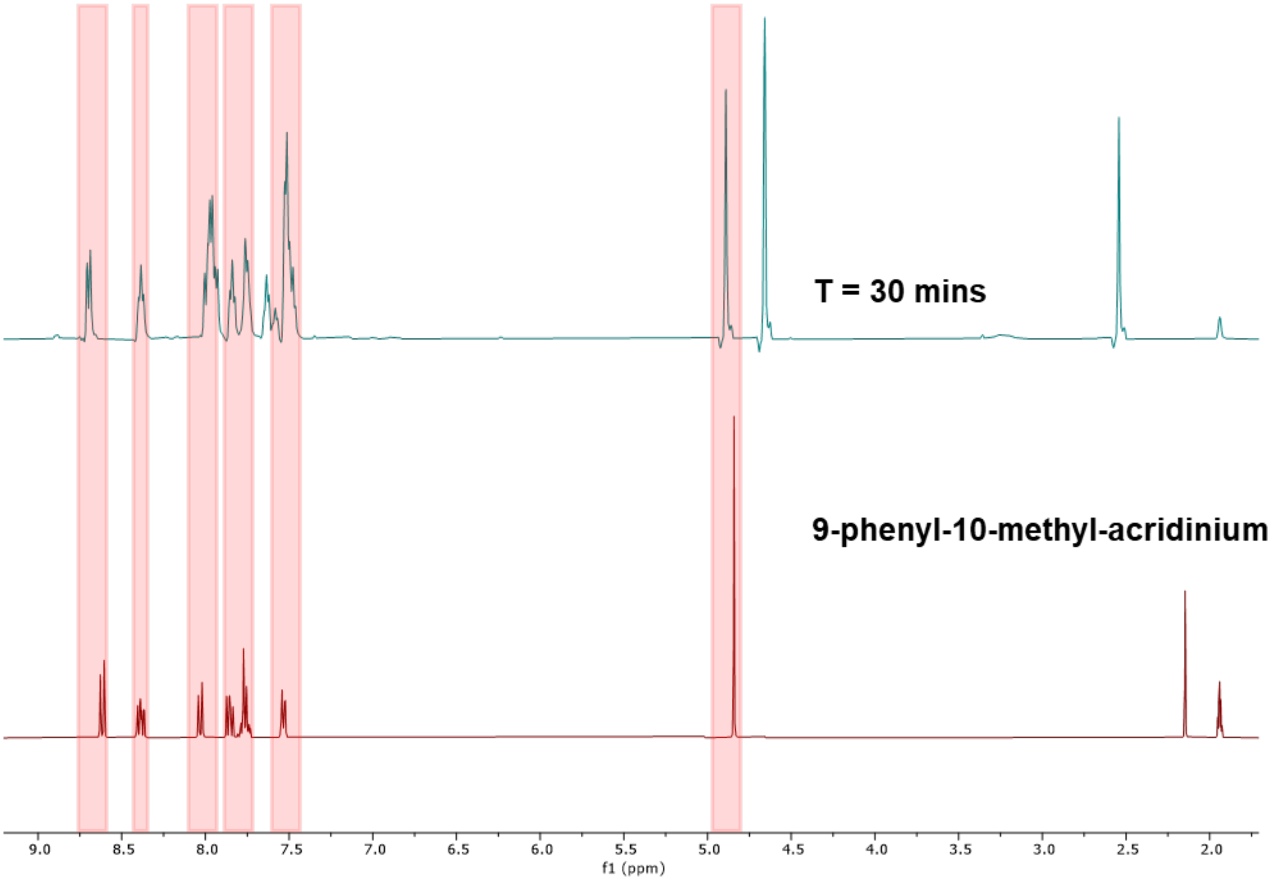

Figure S18. ^{1}H NMR spectroscopic comparison verifying the production of 9-phenyl-10-methyl-acridinium bromide (highlighted in red) from the photoreaction of MPA and 2-bromoacetophenone in the presence of [**Ir**]$^{+}$.

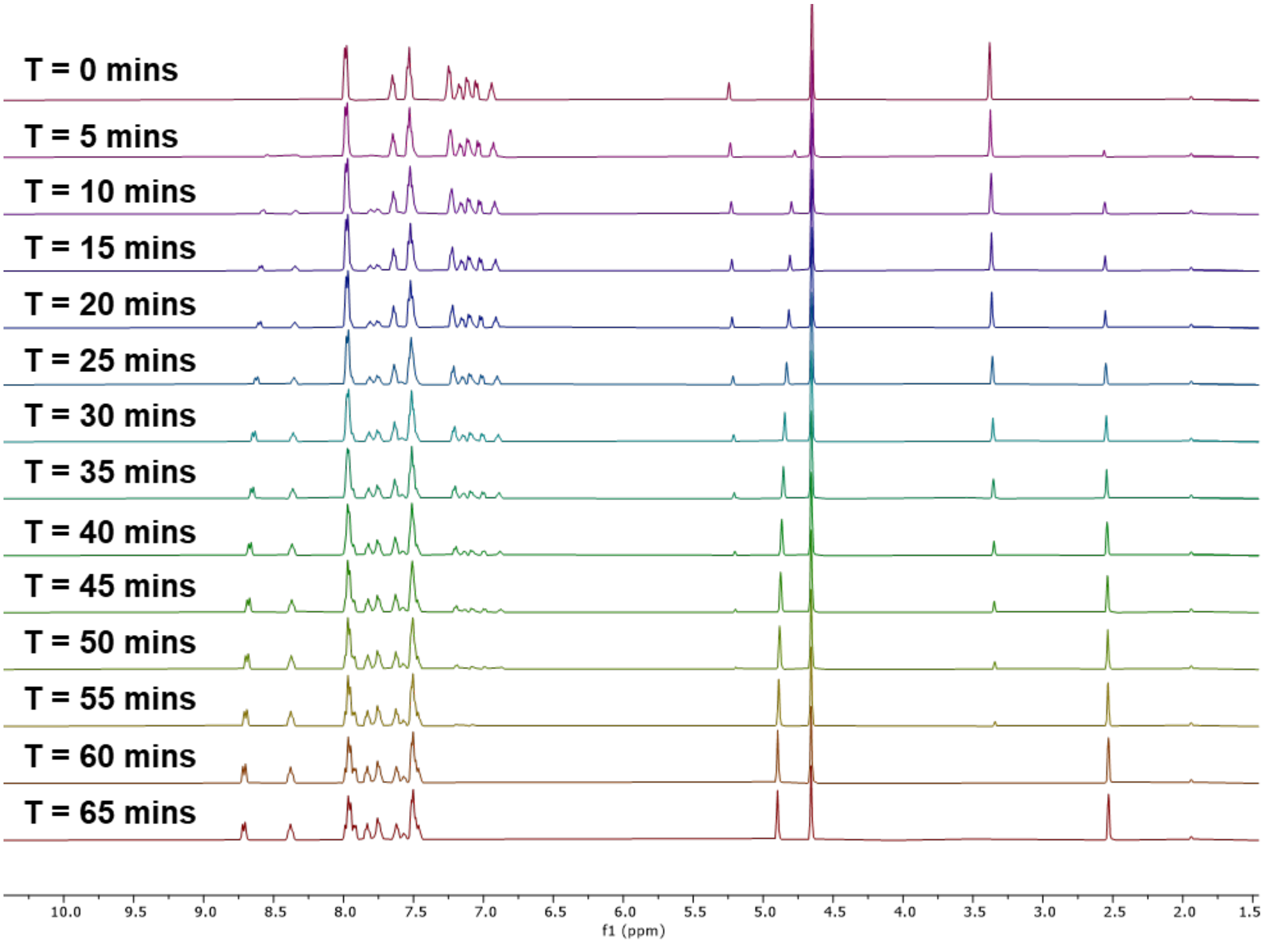

Figure S19. ^{1}H NMR spectra monitoring of the photooxidation of 10-methyl-9-phenyl-9,10-dihydroacridine in the presence of excess 2-bromoacetophenone and 0.1 mol% [**Ir**]$^{+}$.

Note: NMR spectra were collected after 5 min increments of irradiation. The reaction was complete within 65 min.

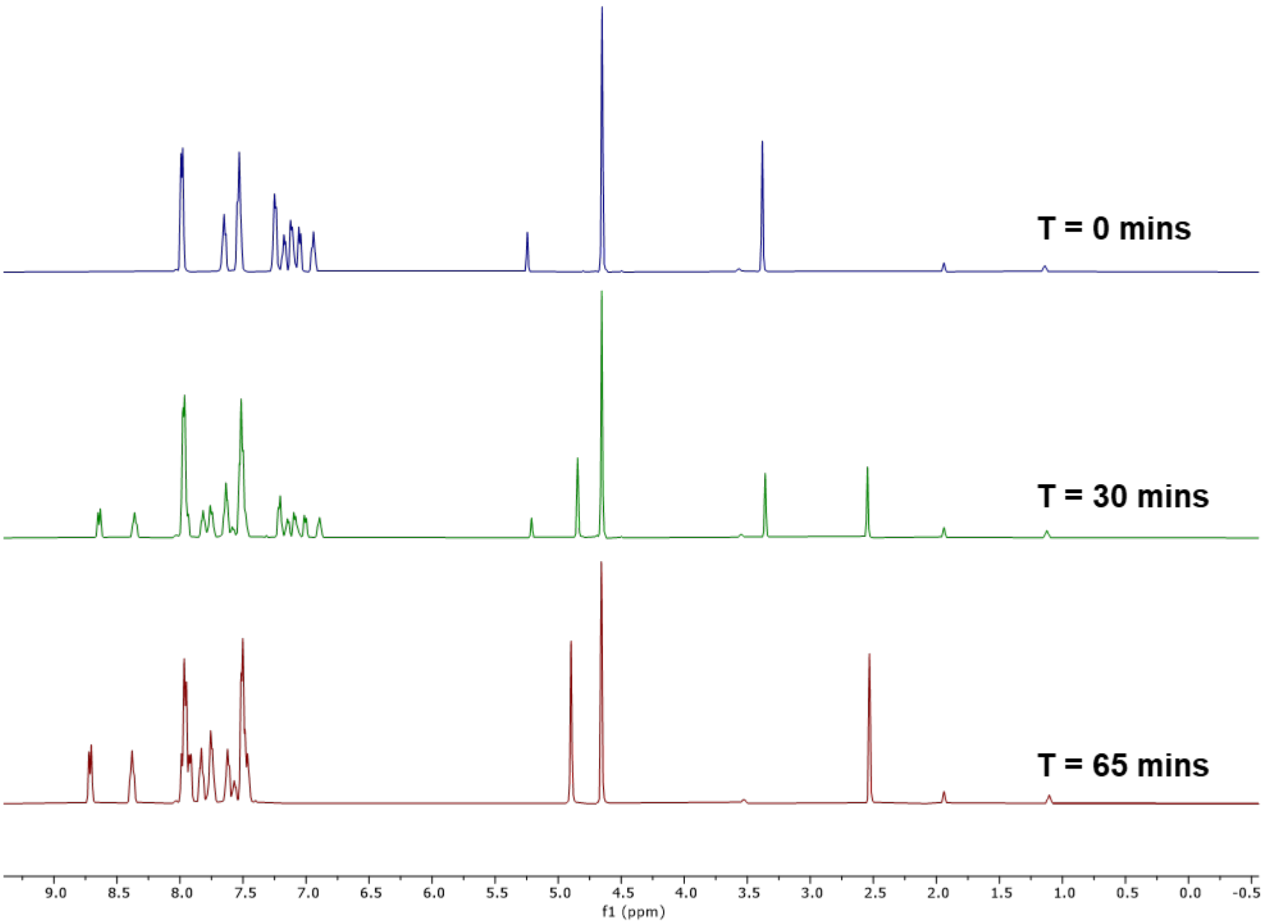

Figure S20. ^{1}H NMR spectra monitoring of the photooxidation of 10-methyl-9-phenyl-9,10-dihydroacridine in the presence of excess 2-bromoacetophenone and 0.1 mol% [**Ir**]$^+$.

Note: NMR spectra were collected after 5 min increments of irradiation. The stackplot shows three selected spectra for a clearer view. The reaction was complete within 65 min.

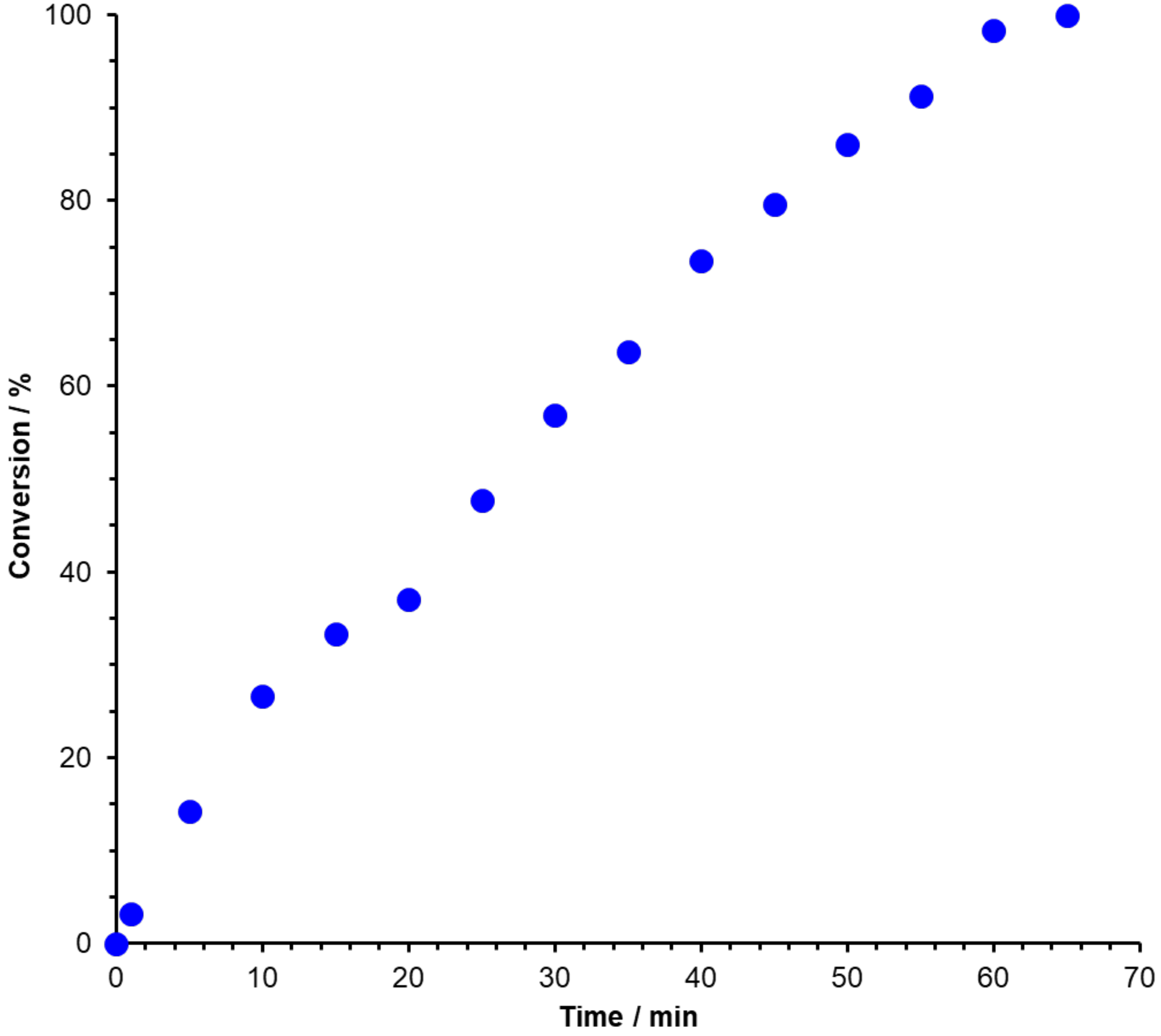

Figure S21. Conversion (as monitored by ^{1}H NMR) over time of 10-methyl-9-phenyl-9,10-dihydroacridine in the presence of 3 equivalents of 2-bromoacetophenone and 0.1 mol% [**Ir**]$^{+}$ under 740 nm illumination.

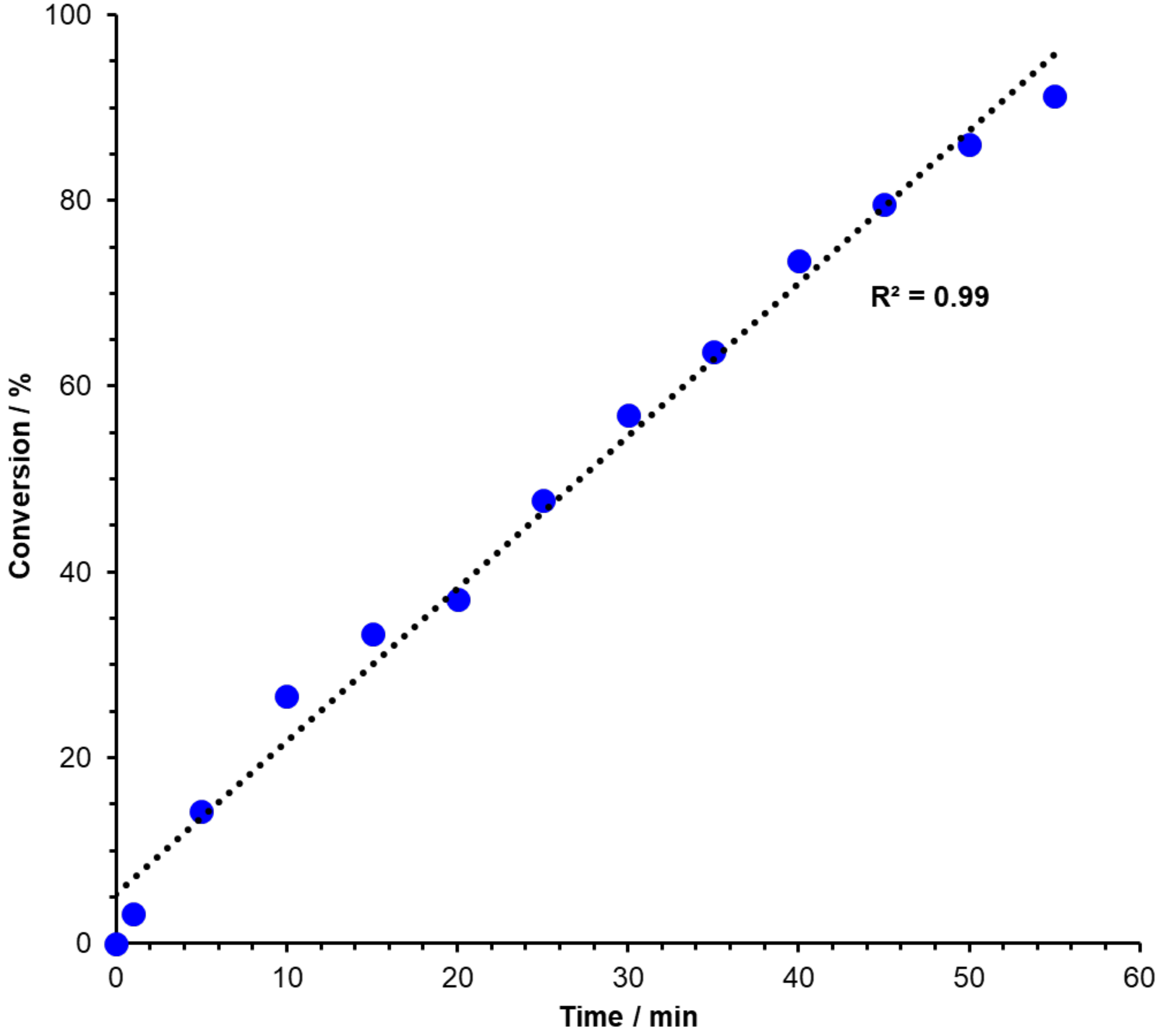

Figure S22. Expansion of the early time points of photooxidation of 10-methyl-9-phenyl-9,10-dihydroacridine in the presence of 3 equivalents of 2-bromoacetophenone and 0.1 mol% [**Ir**]$^+$ under 740 nm illumination.

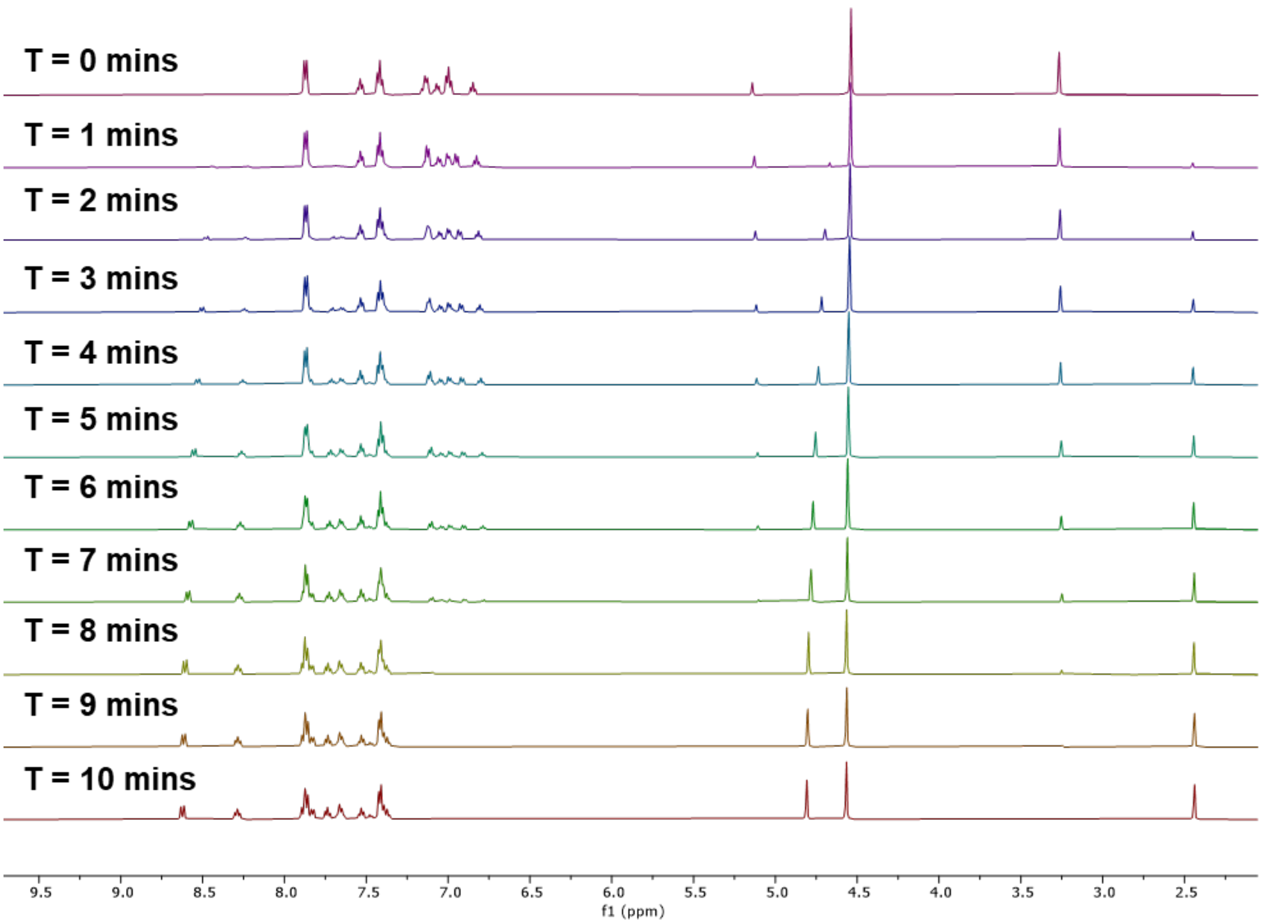

Figure S23. ^{1}H NMR spectra monitoring the photooxidation of 10-methyl-9-phenyl-9,10-dihydroacridine in the presence of 3 equivalents of 2-bromoacetophenone and 2 mol% [**Ir**]$^{+}$ under 740 nm illumination

Note: NMR spectra were collected after 1 min increments of illumination. The reaction was complete within 10 min.

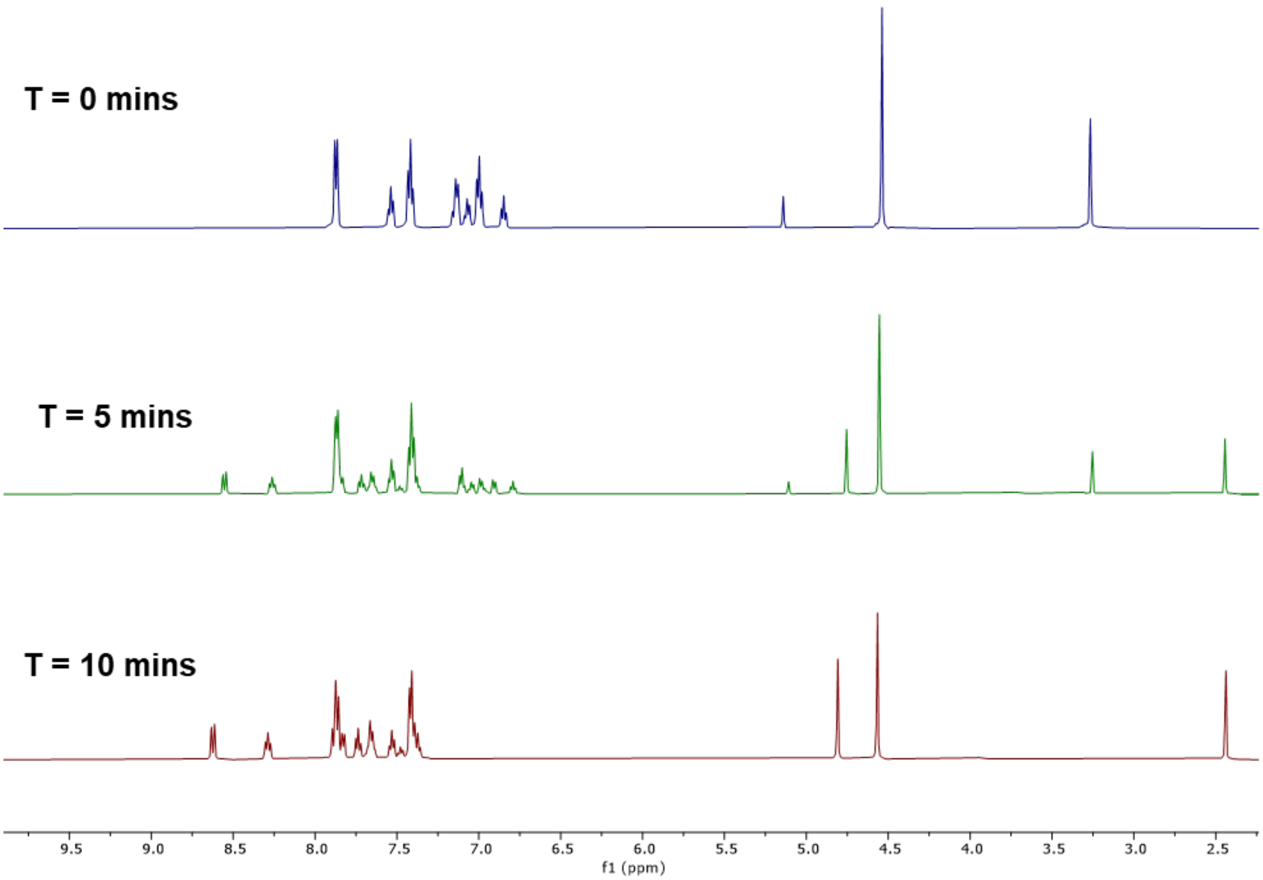

Figure S24. ^{1}H NMR spectra monitoring the photooxidation of 10-methyl-9-phenyl-9,10-dihydroacridine in the presence of 3 equivalents of 2-bromoacetophenone and 2 mol% [**Ir**]$^{+}$.

Note: NMR spectra were collected after 1 min increments of illumination. The reaction was complete within 10 min.

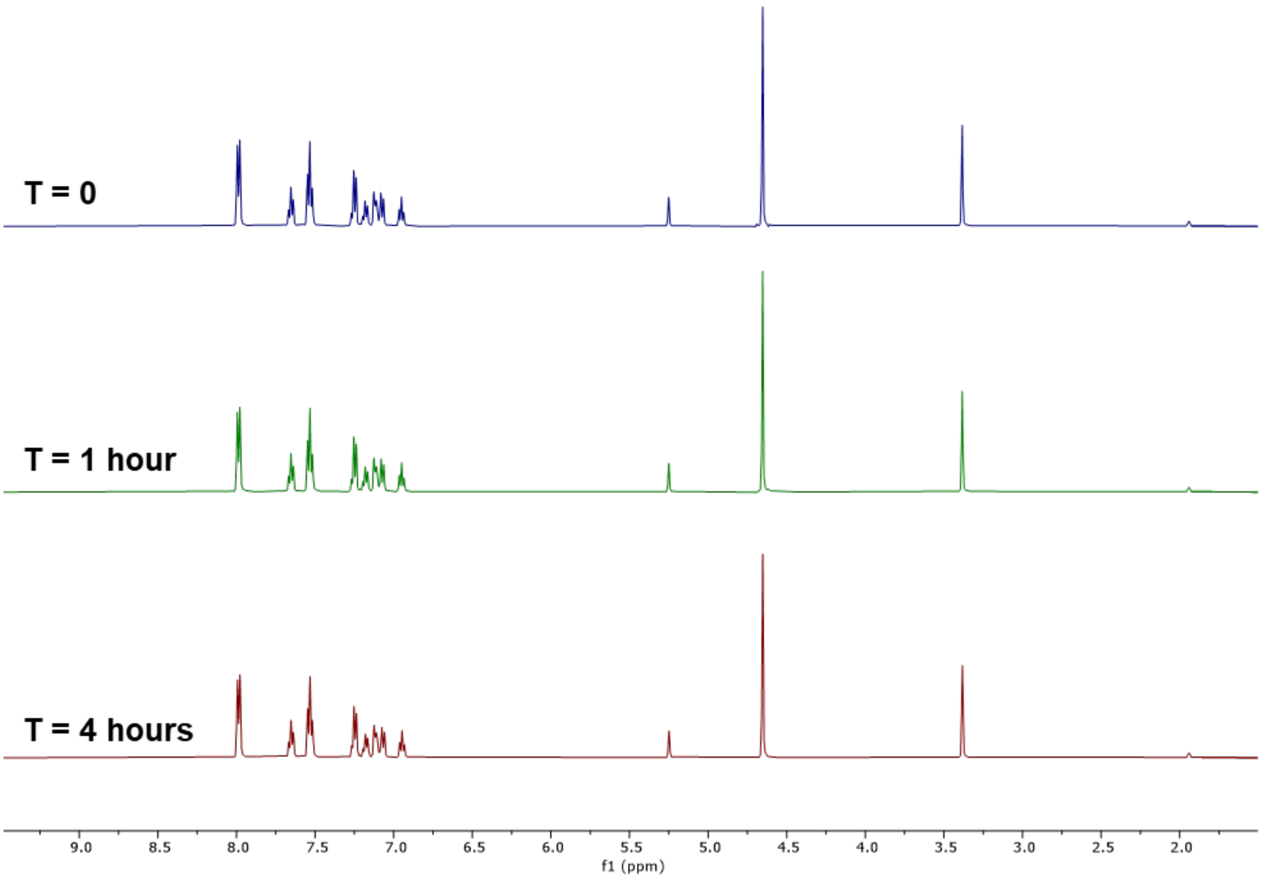

Figure S25. ^{1}H NMR spectra monitoring the reaction of 10-methyl-9-phenyl-9,10-dihydroacridine in the presence of 3 equivalents of 2-bromoacetophenone and 2 mol% [**Ir**]$^{+}$ in the dark.

No conversion was observed.

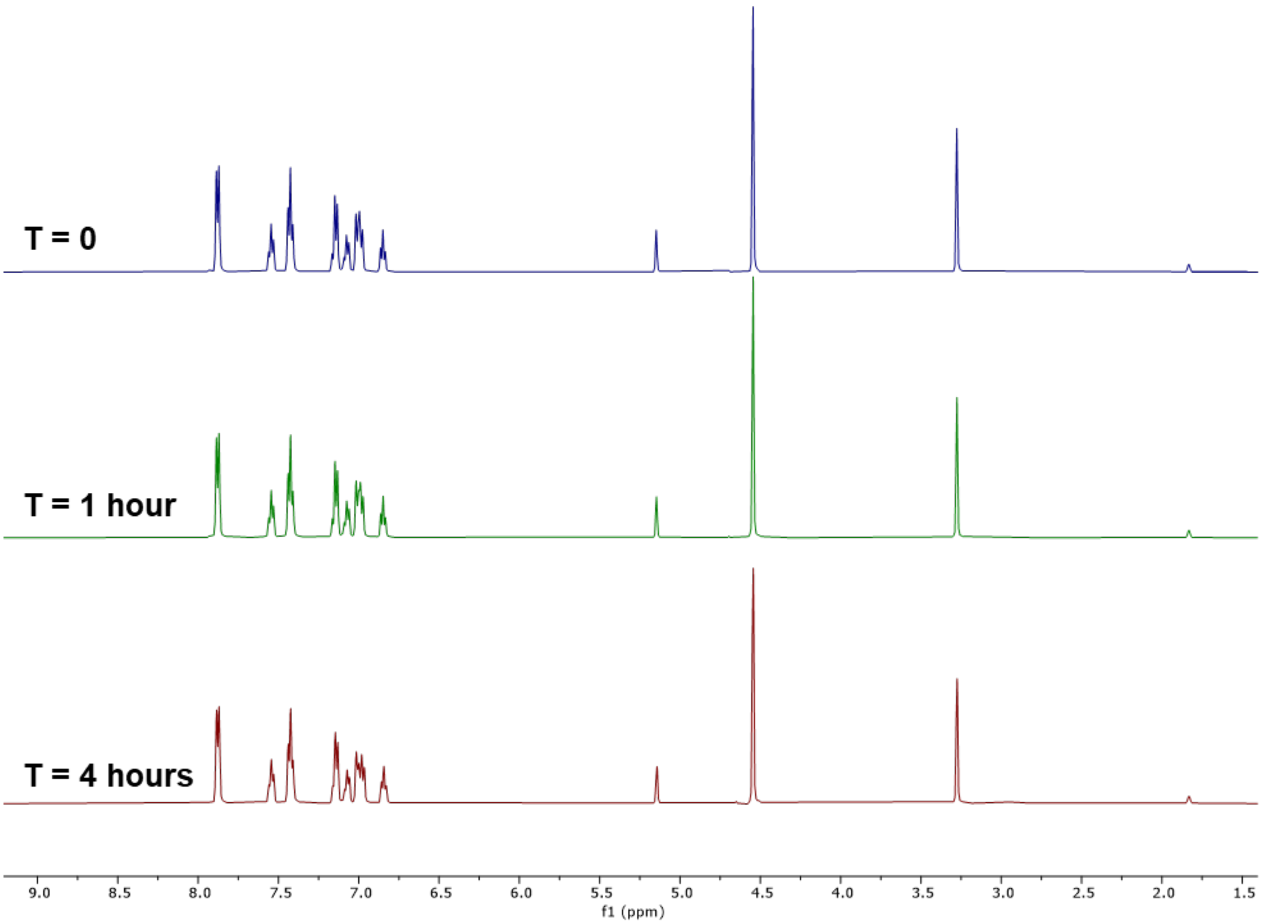

Figure S26. ^{1}H NMR spectra monitoring the photoreaction of 10-methyl-9-phenyl-9,10-dihydroacridine in the presence of 3 equivalents of 2-bromoacetophenone under 740 nm illumination but with no added [**Ir**]$^+$.

No conversion was observed.

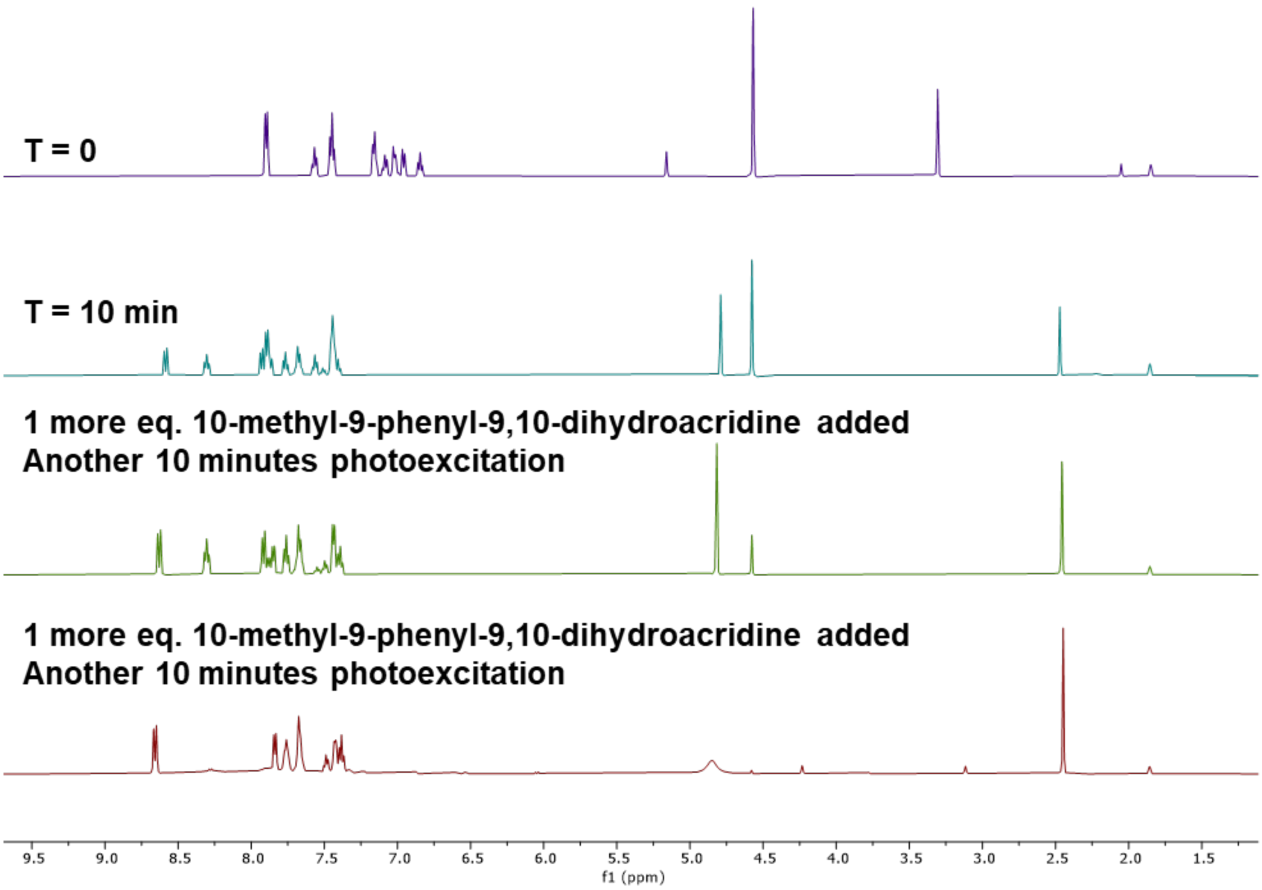

Figure S27. ^{1}H NMR spectra monitoring the photoreaction of 10-methyl-9-phenyl-9,10-dihydroacridine (MPA) in the presence of 3 equivalents of 2-bromoacetophenone and 2 mol% [**Ir**]$^+$ under 740 nm illumination with additional portions of MPA introduced.

Observations: At 10 min of irradiation, complete consumption of MPA was observed. At this point, an additional equivalent of MPA was added, and the reaction was seen to progress further. The addition of MPA was repeated after another 10 min of illumination.

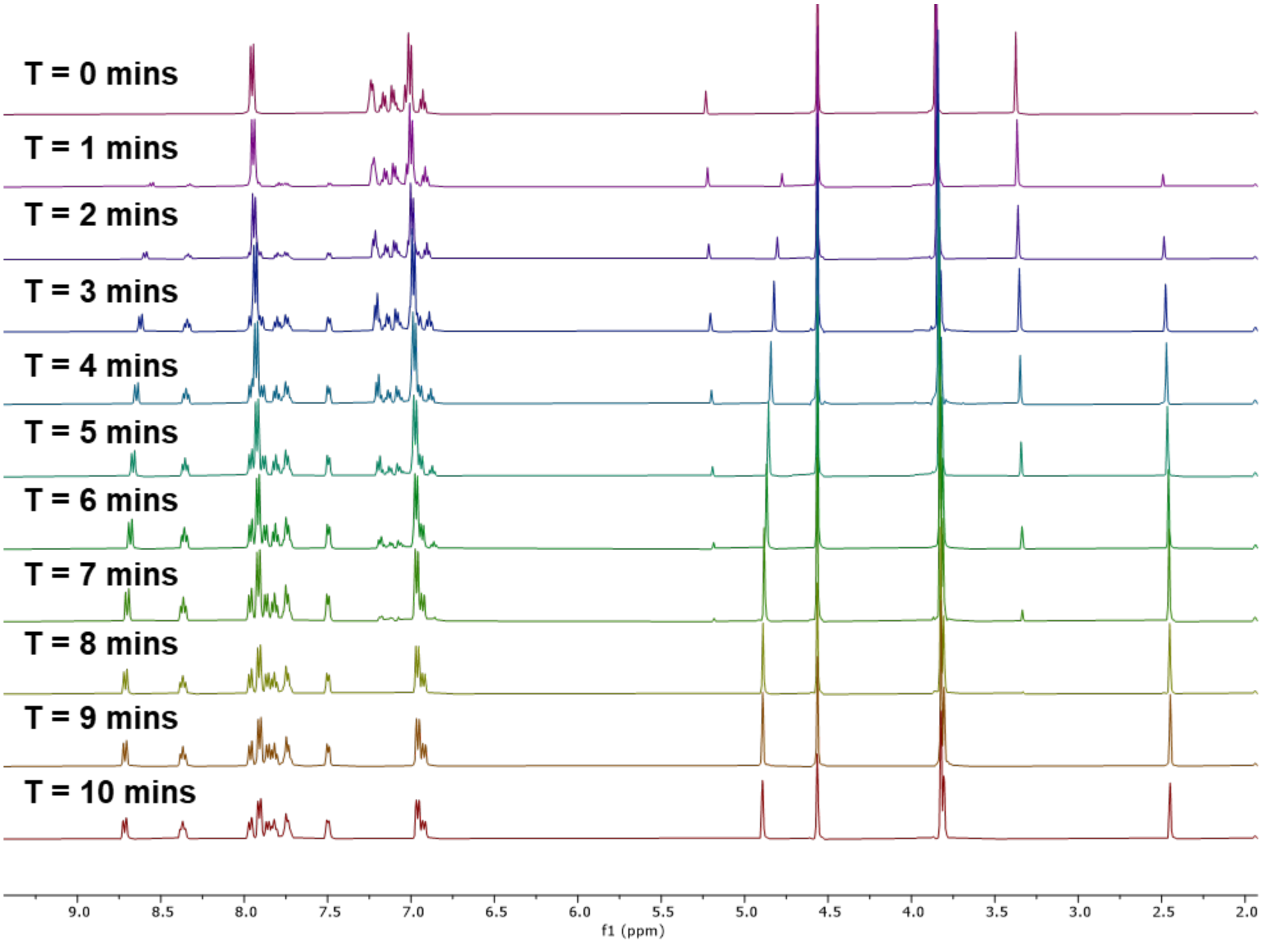

Figure S28. ^1H NMR spectra monitoring the photooxidation of 10-methyl-9-phenyl-9,10-dihydroacridine in the presence of 3 equivalents of 2-bromo-4′-methoxyacetophenone and 2 mol% [**Ir**]$^+$ under 740 nm light.

Conditions: NMR spectra were collected after 1 min increments of illumination. The reaction was complete within 10 min.

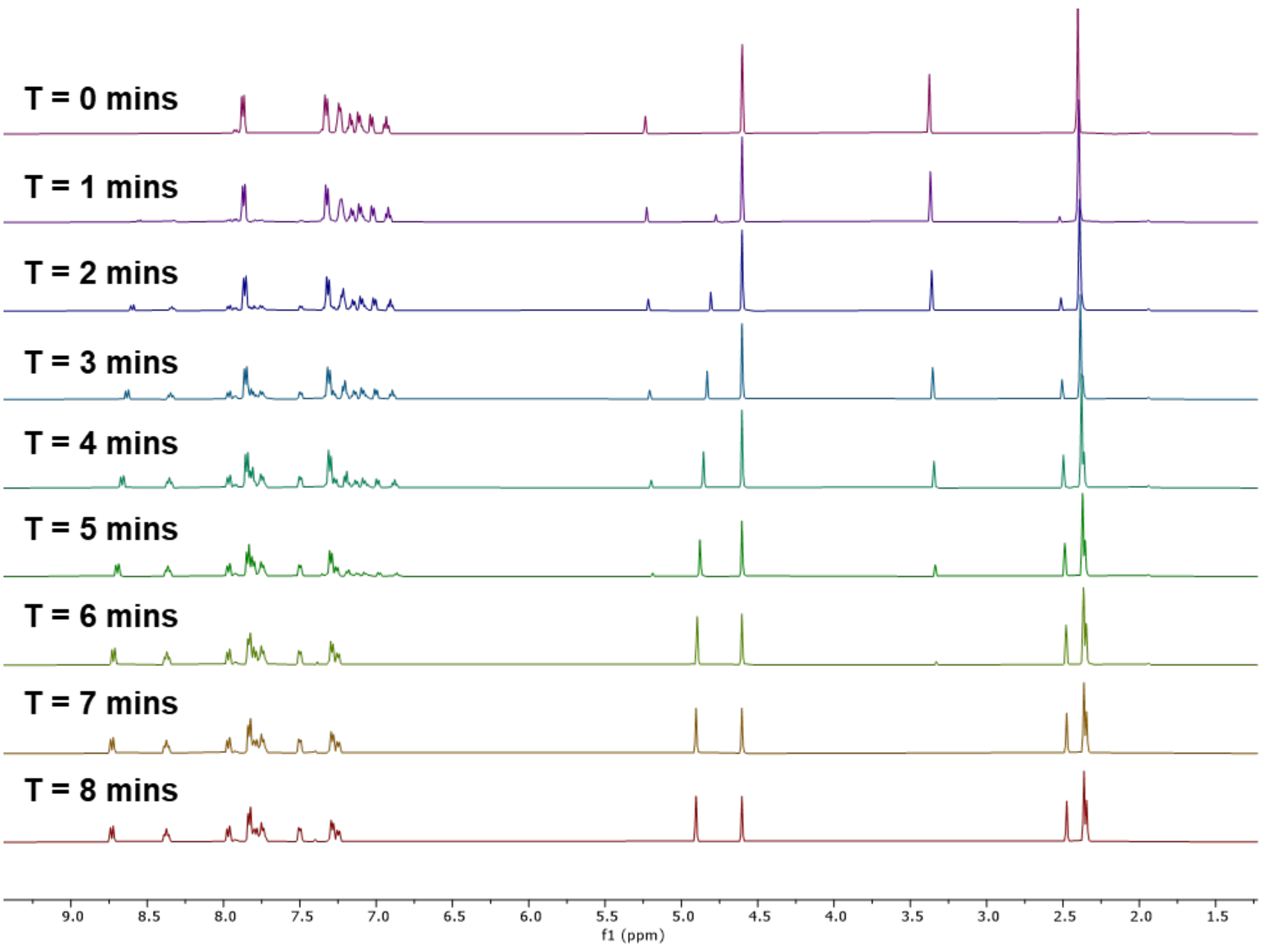

Figure S29. ^{1}H NMR spectra monitoring the photooxidation of 10-methyl-9-phenyl-9,10-dihydroacridine in the presence of 3 equivalents of 2-bromo-4′-methylacetophenone, 2 mol% [**Ir**]$^{+}$ and 740 nm light.

Conditions: NMR spectra were collected after 1 min increments of illumination. The reaction was complete within 8 min.

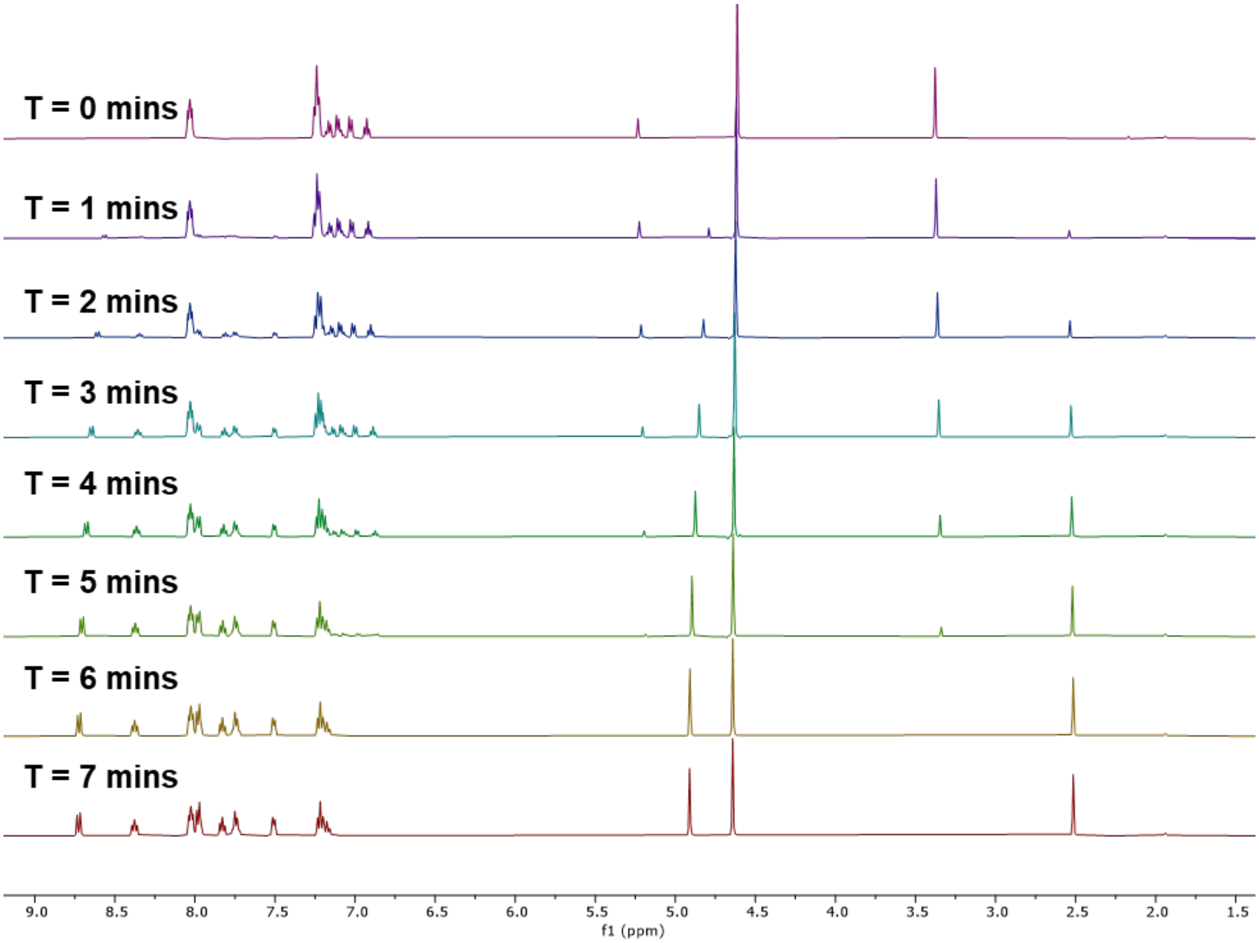

Figure S30. ^{1}H NMR spectra monitoring the photooxidation of 10-methyl-9-phenyl-9,10-dihydroacridine in the presence of 3 equivalents of 2-bromo-4′-fluoroacetophenone, 2 mol% [**Ir**]$^+$ and 740 nm light.

Conditions: NMR spectra were collected after 1 min increments of illumination. The reaction was complete within 7 min.

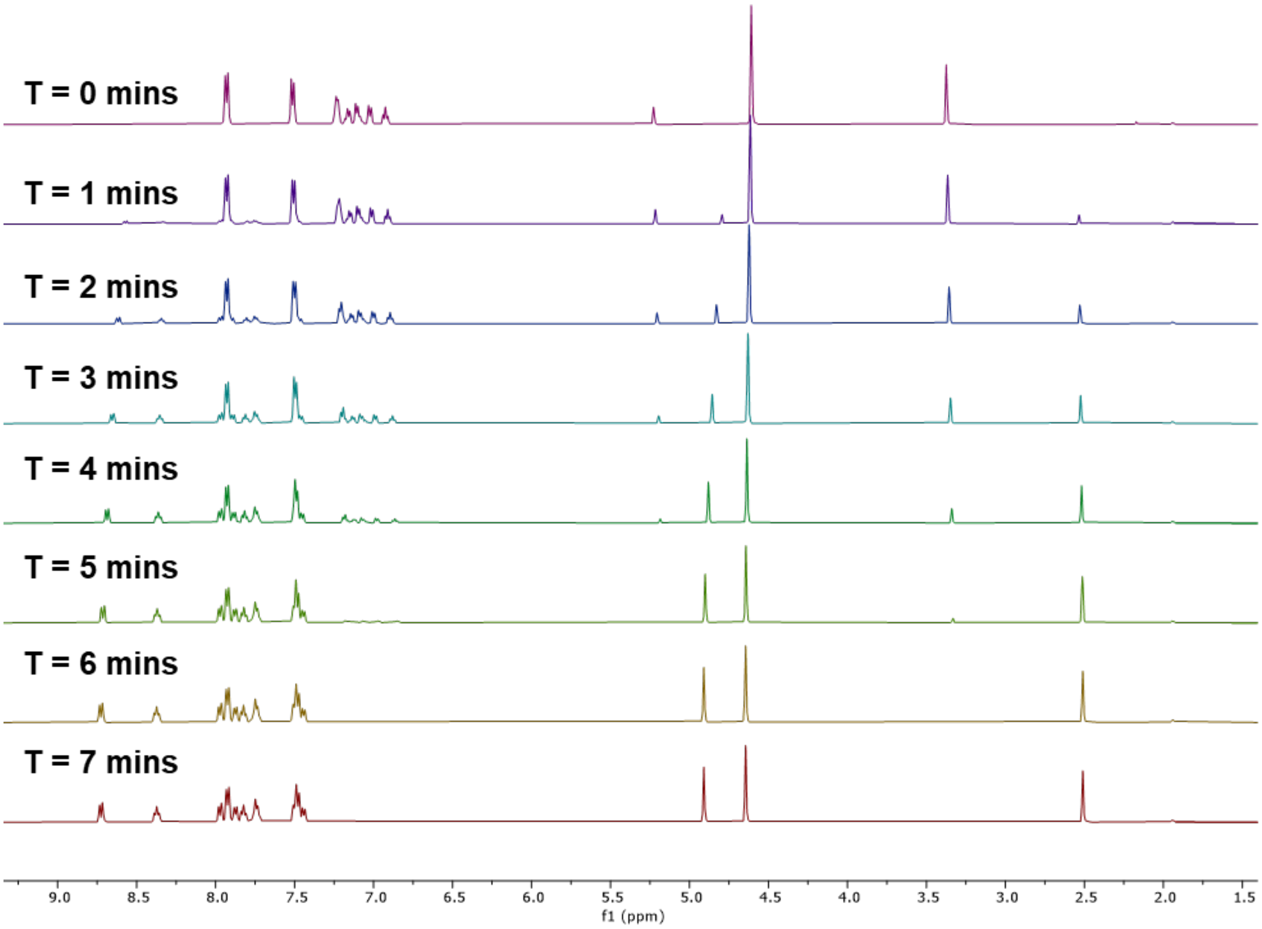

Figure S31. ^{1}H NMR spectra monitoring the photooxidation of 10-methyl-9-phenyl-9,10-dihydroacridine in the presence of 3 equivalents of 2-bromo-4′-chloroacetophenone, 2 mol% [**Ir**]$^{+}$ and 740 nm light.

Conditions: NMR spectra were collected after 1 min increments of illumination. The reaction was complete within 7 min.

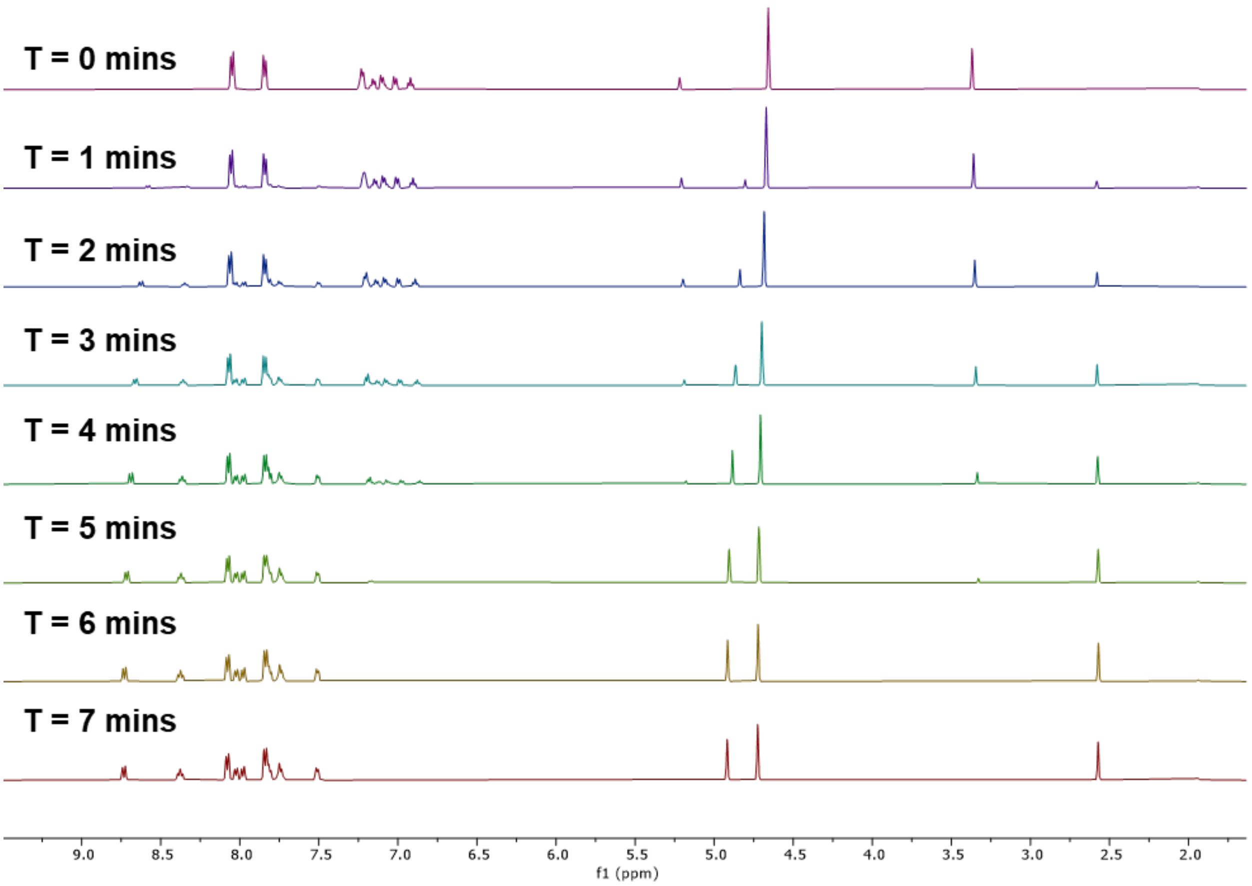

Figure S32. ^{1}H NMR spectra monitoring the photooxidation of 10-methyl-9-phenyl-9,10-dihydroacridine in the presence of 3 equivalents of 2-bromo-4′-cyanoacetophenone, 2 mol% [**Ir**]$^+$ and 740 nm light.

Conditions: NMR spectra were collected after 1 min increments of illumination. The reaction was complete within 7 min.

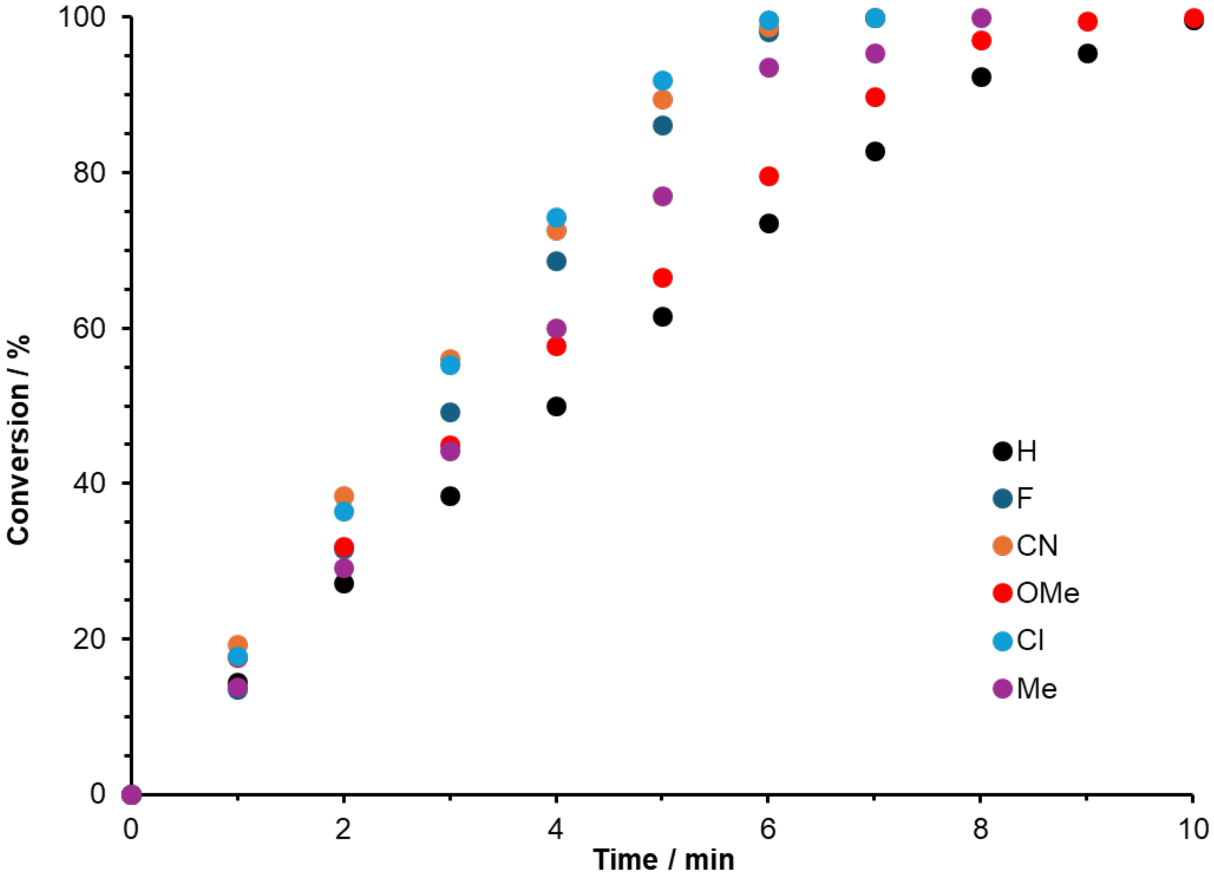

Figure S33. Comparison of the ^{1}H NMR conversion of MPA in the presence of threefold-excess of various 2-bromo-4′-functionalized acetophenones (2 mol% [**Ir**]$^{+}$, 740 nm irradiation).

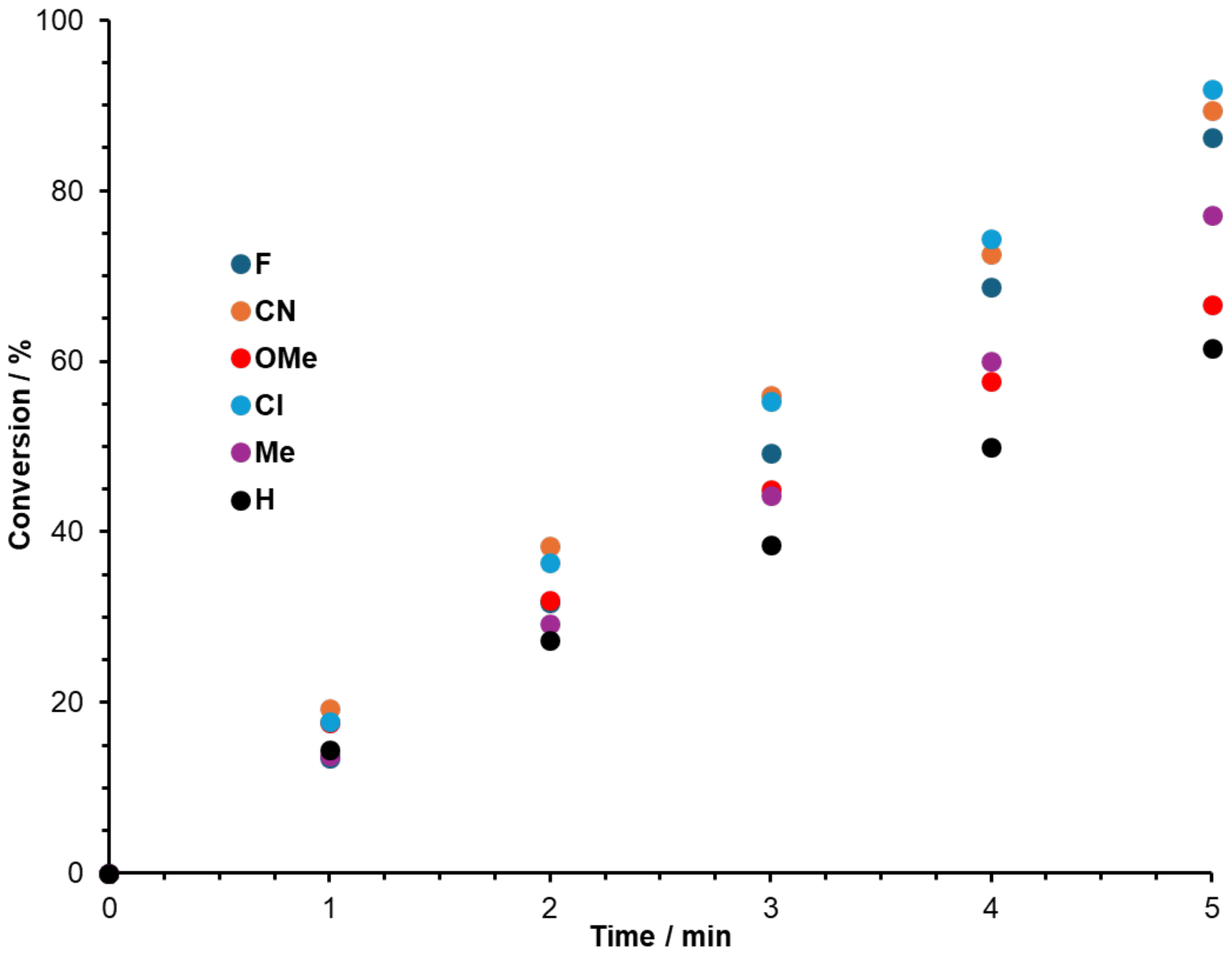

Figure S34. Expansion of the first 5 min of the ^{1}H NMR conversion of MPA in the presence of threefold-excess of various 2-bromo-4′-functionalized acetophenones (2 mol% [**Ir**]$^+$, 740 nm irradiation).

This linear data set was used to calculate the rate of reaction.

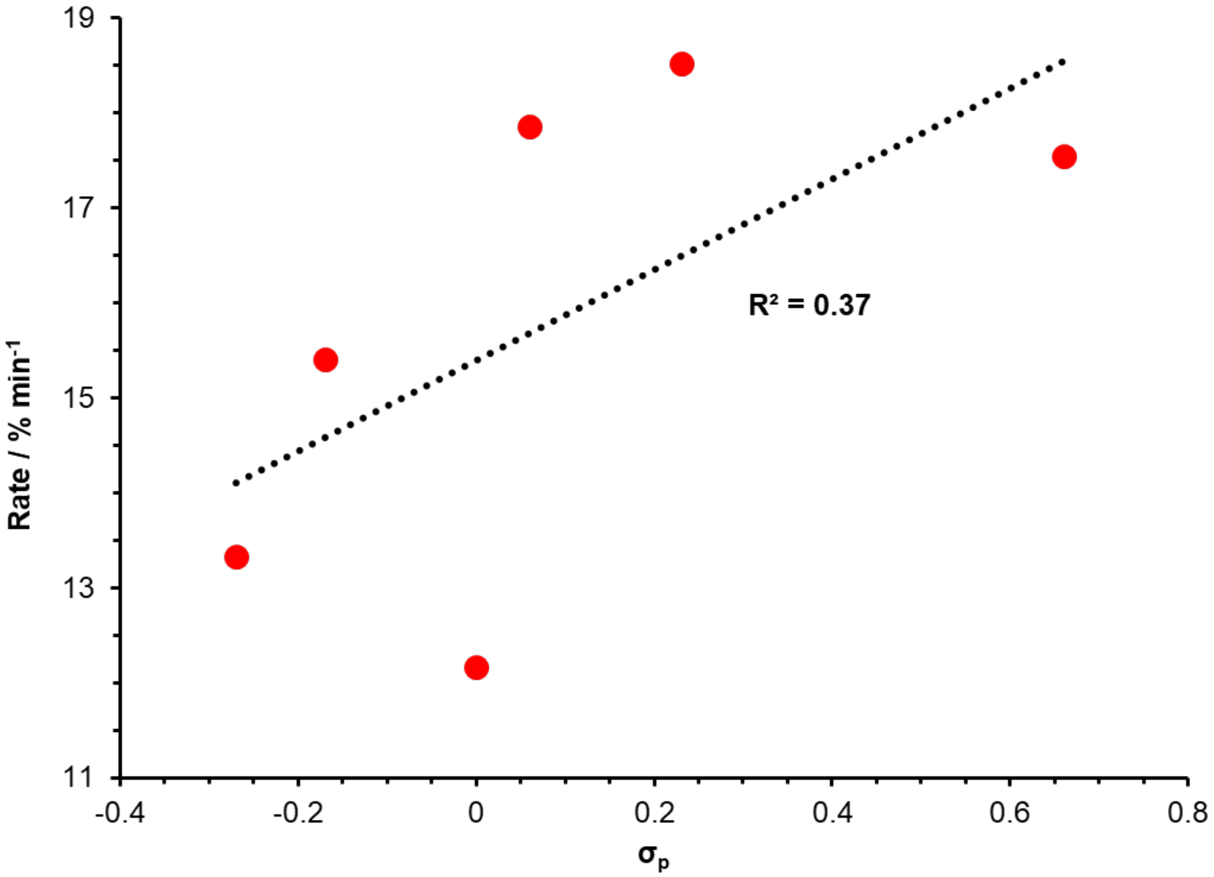

Figure S35. Comparison of the rate of conversion of MPA in the presence of threefold-excess of various 2-bromo-4′-functionalized acetophenones (2 mol% [**Ir**]$^+$, 740 nm irradiation) vs. the Hammett parameter (σ_p) of the functional group on 2-bromo-4′-functionalized acetophenones.

Note the low correlation.

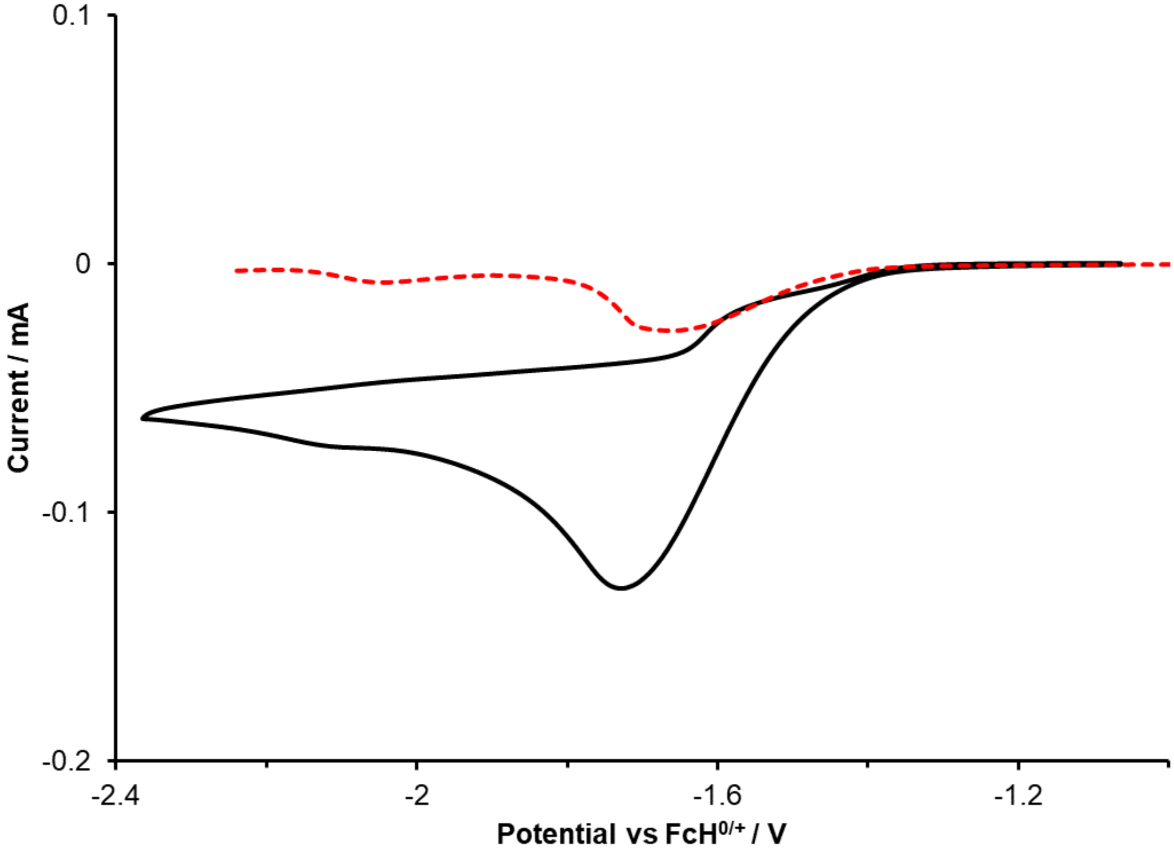

Figure S36. Overlap of cyclic voltammogram (CV; —) and differential pulse voltammogram (DPV; ---) of 2-bromo-4′-methoxyacetophenone.

Conditions: 6 mM acetonitrile solution containing 0.1 M [$n$$Bu_4N$]$PF_6$ electrolyte at a CV scan rate of 100 mV/s. The $E_{1/2}$ of the irreversible reduction is estimated from the DPV to be -1.66 V vs $FcH^{0/+}$.

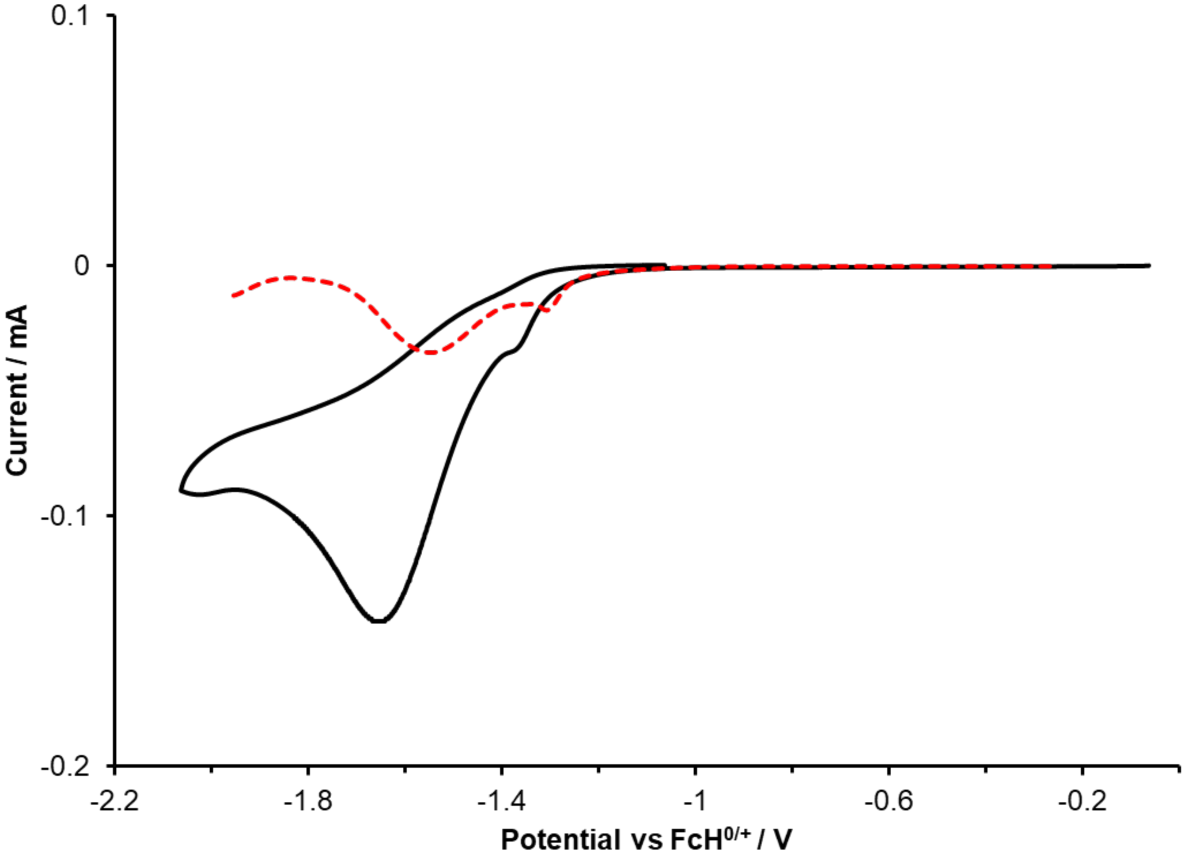

Figure S37. Overlap of cyclic voltammogram (CV; —) and differential pulse voltammogram (DPV; ---) of 2-bromo-4′-methylacetophenone.

Conditions: 6 mM acetonitrile solution containing 0.1 M [*n*Bu_4N]PF_6 electrolyte at a CV scan rate of 100 mV/s. The $E_{1/2}$ of the irreversible reduction is estimated from the DPV to be -1.55 V vs $FcH^{0/+}$.

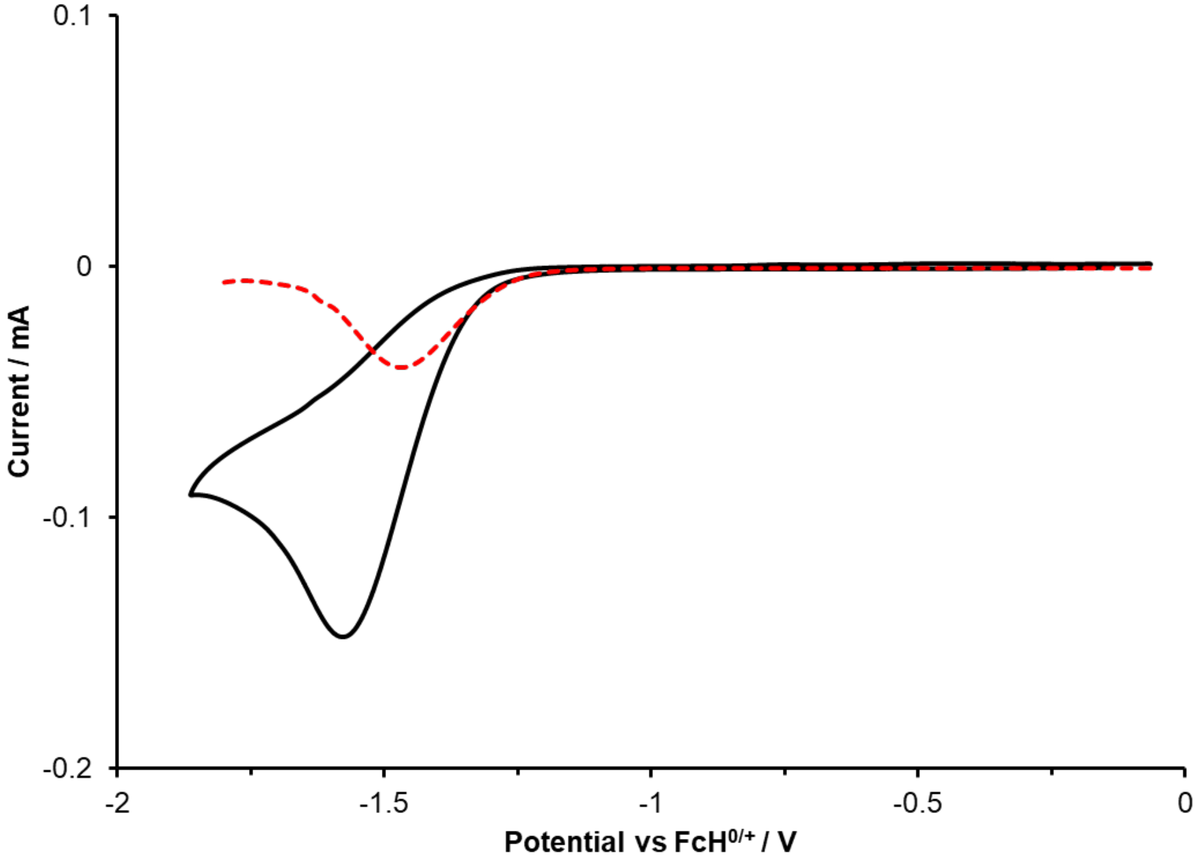

Figure S38. Overlap of cyclic voltammogram (CV; —) and differential pulse voltammogram (DPV; ---) of 2-bromo-4′-fluoroacetophenone.

Conditions: 6 mM acetonitrile solution containing 0.1 M [nBu$_4$N]PF$_6$ electrolyte at a CV scan rate of 100 mV/s. The $E_{1/2}$ of the irreversible reduction is estimated from the DPV to be -1.47 V vs FcH$^{0/+}$.

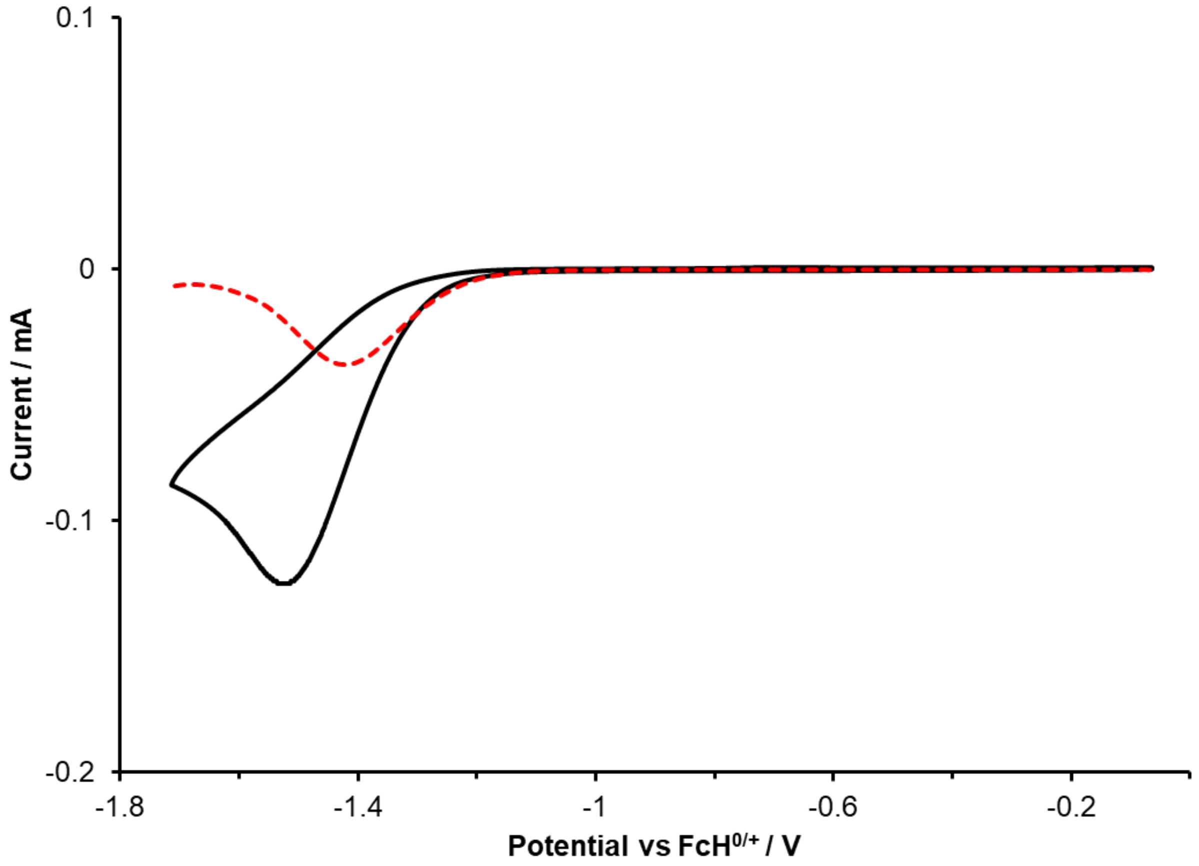

Figure S39. Overlap of cyclic voltammogram (CV; —) and differential pulse voltammogram (DPV; ---) of 2-bromo-4′-chloroacetophenone.

Conditions: 6 mM acetonitrile solution containing 0.1 M [nBu$_4$N]PF$_6$ electrolyte at a CV scan rate of 100 mV/s. The $E_{1/2}$ of the irreversible reduction is estimated from the DPV to be -1.42 V vs FcH$^{0/+}$.

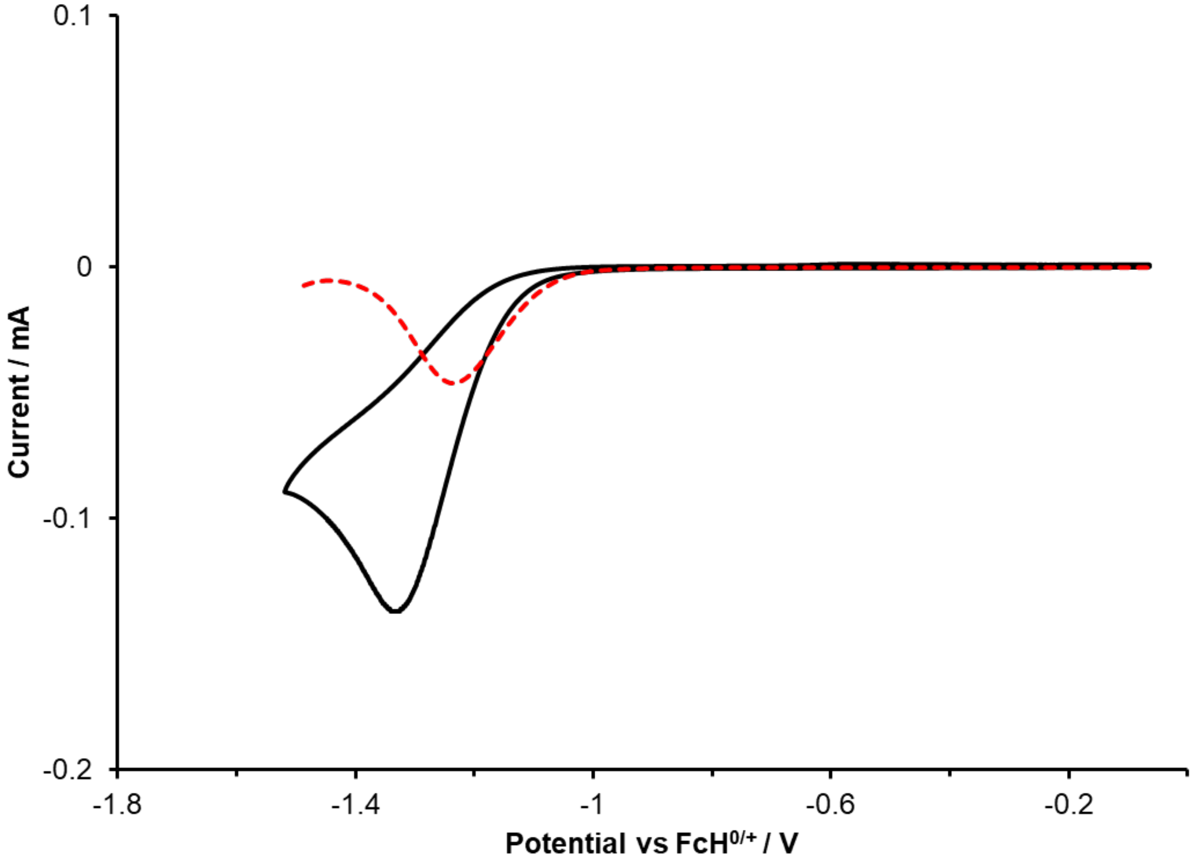

Figure S40. Overlap of cyclic voltammogram (CV; —) and differential pulse voltammogram (DPV; ---) of 2-bromo-4′-cyanoacetophenone.

Conditions: 6 mM acetonitrile solution containing 0.1 M [nBu$_4$N]PF$_6$ electrolyte at a CV scan rate of 100 mV/s. The $E_{1/2}$ of the irreversible reduction is estimated from the DPV to be -1.24 V vs FcH$^{0/+}$.

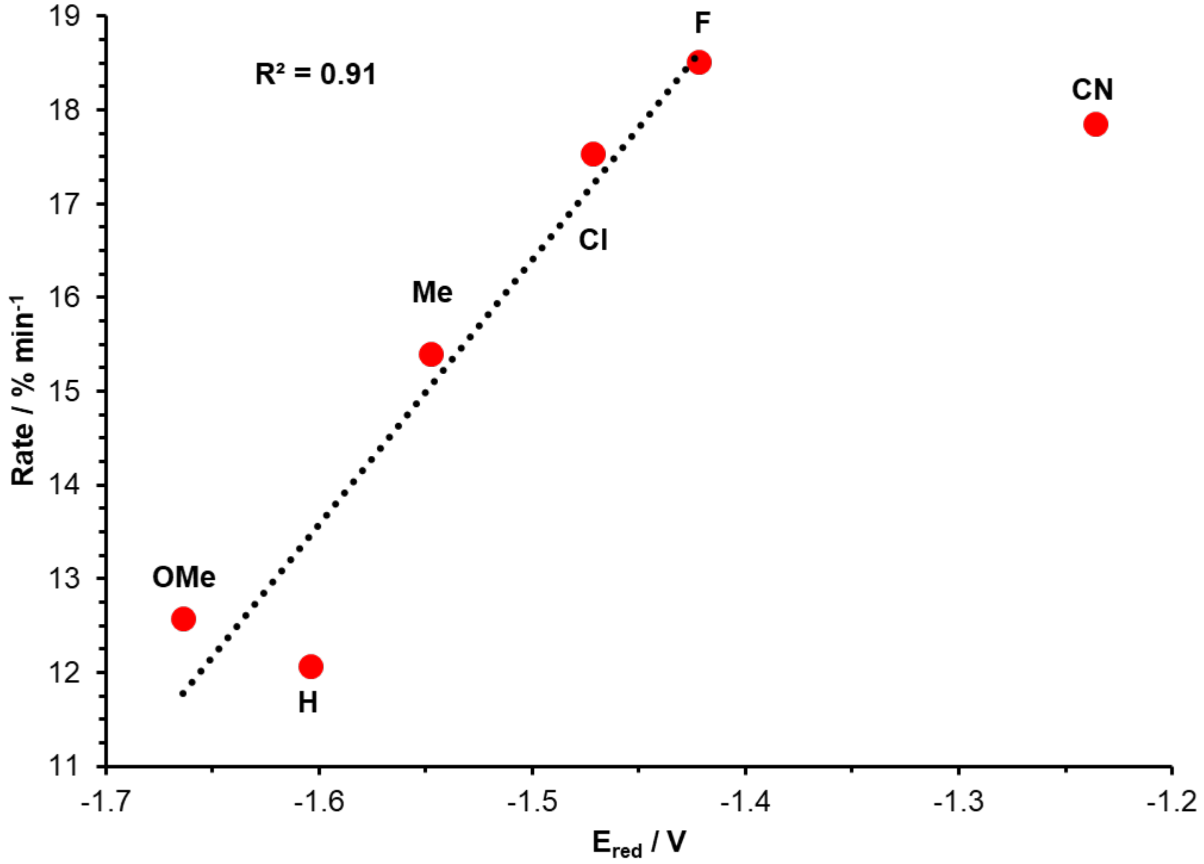

Figure S41. Comparison of the conversion rate of MPA photooxidation versus the reduction potential (E_{red}) of 2-bromo-4′-functionalized acetophenones.

Note the strong correlation up to ~ -1.4 V. At more positive potentials, the rate appears to be diffusion controlled.

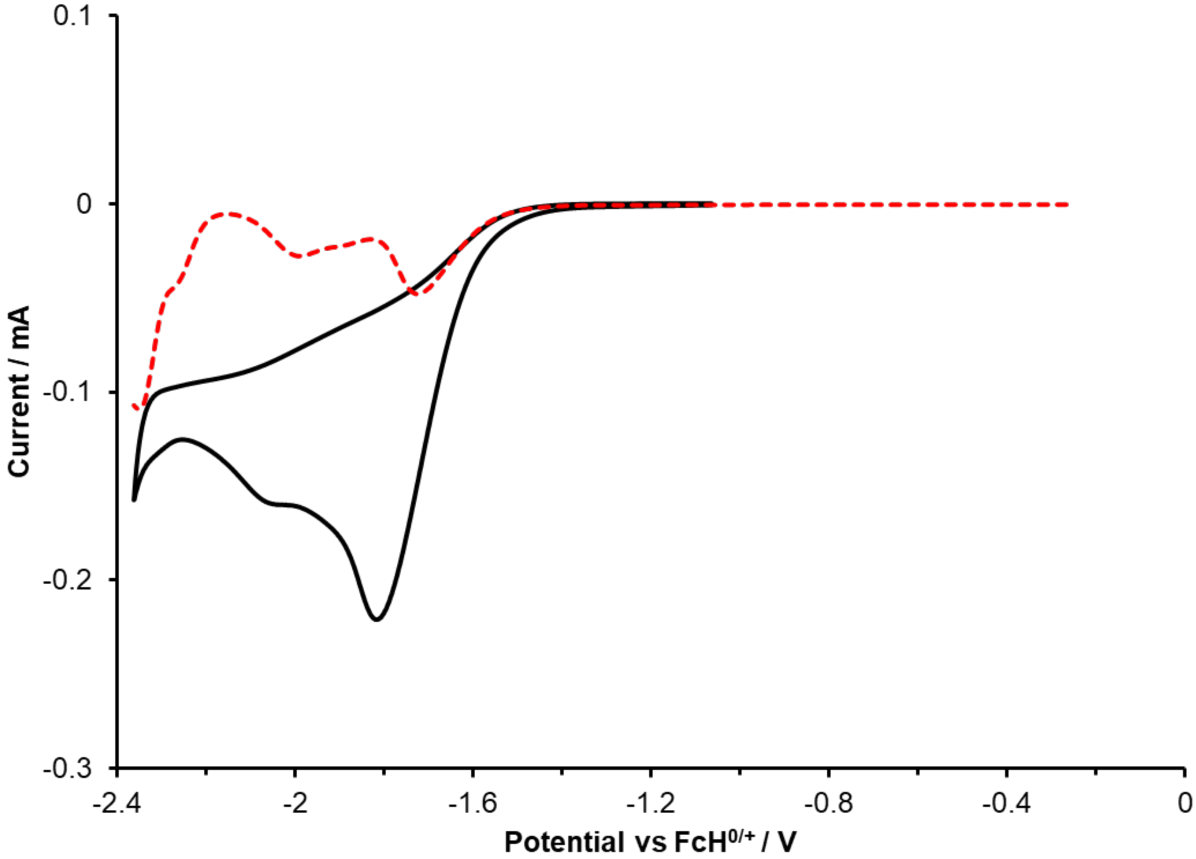

Figure S42. Overlap of cyclic voltammogram (CV; —) and differential pulse voltammogram (DPV; ---) of 2-chloroacetophenone.

Conditions: 6 mM acetonitrile solution containing 0.1 M [*n*Bu_4N]PF_6 electrolyte at a CV scan rate of 100 mV/s. The $E_{1/2}$ of the irreversible reduction is estimated from the DPV to be -1.73 V vs FcH$^{0/+}$.

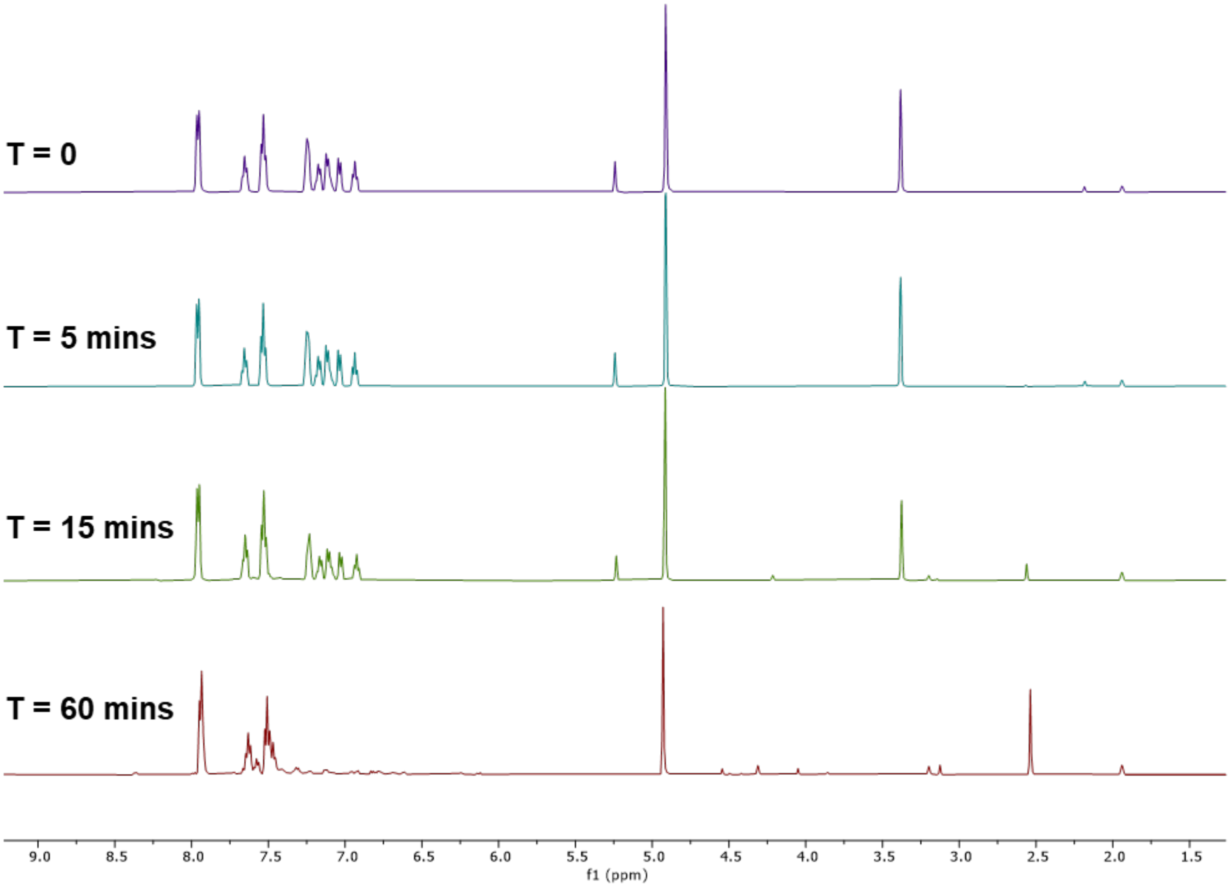

Figure S43. ^{1}H NMR spectra monitoring the photoreaction of 10-methyl-9-phenyl-9,10-dihydroacridine in the presence of 3 equivalents of 2-chloroacetophenone, 2 mol % [**Ir**]$^{+}$ and 740 nm light.

While full consumption of MPA is observed, numerous products are evident.

EXPERIMENTAL DETAILS

Unless otherwise stated, all air sensitive manipulations were performed inside an inert-atmosphere glove box (N_2) or using standard Schlenk techniques (Ar). Chemicals phenacyl bromide (TCI America), 2-bromo-4'-chloroacetophenone (Aldrich), 2-bromo-4'-cyanoacetophenone (Aldrich), 2-bromo-4'-fluoroacetophenone (Aldrich), 2-bromo-4'-methoxyacetophenone (Aldrich), 2-bromo-4'-methylacetophenone (Aldrich), 2-chloroacetophenone (Aldrich), furfural (TCI America), 4-methoxybenzeneboronic acid (Strem Chemicals), and *N*,*N*-diisopropylethylamine (Aldrich) were purchased from commercial suppliers as reagent grade or better and used as received. [Ir(ppy)$_2$(*p*-biphe)]PF$_6$ ([**Ir**]PF$_6$)[1] was synthesized following a published procedure. NMR solvents were used as received. NMR spectra were recorded on a Bruker Avance 400 MHz or a Bruker Avance-III 500 MHz. ^{1}H NMR spectra were referenced to residual solvent peaks.

UV-Vis Absorbance

Electronic absorption spectra were acquired on solutions prepared under ambient conditions in CH_3CN in 1 cm path length quartz cuvettes using a JASCO V-770 spectrophotometer; spectra measured before and after all transient absorption measurements (collected to ensure integrity of the samples) were collected on a Varian Cary50 spectrophotometer (MSU). Gaussian deconvolution was performed using Igor Pro software.

Electrochemistry

For electrochemical analysis, a 15 mL acetonitrile solution containing ~6 mM of analyte and 0.1 M of [nBu$_4$N]PF$_6$ electrolyte was prepared and degassed under argon. Electrochemical experiments were preformed using a CHI 760c bipotentiostat, a 3 mm diameter glassy carbon

working electrode, a Ag/Ag^+ non-aqueous quasi-reference electrode separated by a Vycor tip, and a Pt wire counter electrode. Cyclic voltammetry was conducted using scan rates of 100 mV/s. Differential Pulse Voltammetry was performed using a 5 mV increment, 50 mV amplitude, 0.05 s pulse width, 0.0167 s sample width, and 0.5 s pulse period. Following the experiment ferrocene was added to the solution as an internal standard and all potentials are reported versus the $FcH^{0/+}$ redox couple.

Spectroelectrochemistry

Spectroelectrochemical data were collected on an Agilent Cary 5000 Series UV-Vis NIR spectrophotometer using a CHI-620C electrochemical analyzer and a room temperature optically transparent thin-layer electrochemical (OTTLE) cell. A 3-electrode system of Pt minigrid (32 wires/cm) working and auxiliary electrodes and an Ag wire pseudo-reference electrode was used.[4]

Time-resolved Absorption Spectroscopy

Nanosecond time-resolved absorption measurements were carried out using an Opotek Vibrant 355 LD Q-switched Nd:YAG laser pumping an Opotek optical parametric oscillator coupled to an Edinburgh Instruments LP980 spectrometer fitted with a Hamamatsu R928 photomultiplier tube and interfaced to a Tektronix TDS 3032C oscilloscope. Samples were dissolved in spectral grade CH_3CN, made in a glove box, and placed in an air-tight 1 cm quartz cuvette with an absorbance in the range of 0.4 – 0.7 at the excitation wavelength. The temperature was controlled using a TC 1 Temperature Controller from Quantum Northwest, and all experiments were conducted at room temperature, 298(2) K. All data were plotted in Igor Pro. The kinetic experiment was carried out 2 separate times, with the data averaged between the runs. The error in the lifetime was determined

from the fit, added to and subtracted from the lifetime of that run to get the lower and higher limits, and then the standard deviation between the experiments was taken to give the error. Following all experiments, a UV-Vis was taken of the sample and compared to an absorbance spectrum taken before to confirm the sample integrity.

Computational Details

All computational work was carried out using Orca v.5.0.2.[5–7] The optimized geometries from the reference[1] was used. Only TD-DFT was performed including spin-orbit coupling corrections. The level of theory used in the mentioned reference did not include SOC and did not match the low energy region well, PBE0[8,9] was found to match these transitions the best. ZORA[10] and the OLD-ZORA-TZVP[11] basis set was used on all atoms to account for relativistic effects. Also incorporated was the SMD[12] solvent model (CH_3CN), the resolution of identity approximation (RIJCOSX)[13], to speed up the calculations, with the SARC/J auxiliary basis set.[14] A special grid was placed on the heavy transition metal (RIJCOSX; intaccx: 4.34, 4.34, 4.67; gridx: 2,2,2; specialgridintacc: 9),[13] along with SARC-TZVP[15] and the corresponding auxiliary basis sets.[11,14,16–18] Electron-hole density maps and spin density was visualized in Gabedit,[19] from cube files generated in Multiwfn.[20]

Photocatalytic Experiments

Irradiation of samples was carried out using an EvoluChem PhotoRedOx Box and a non-dimmable, 20 mW/cm^2 740 nm red light (SKU: HCK1012-01-015; voltage 110-220 V, irradiance 20 mW/cm^2). Control reactions protected from light in a closed drawer or without any metal complex added were carried out as noted.

The emission spectrum of the LED used is provide below:

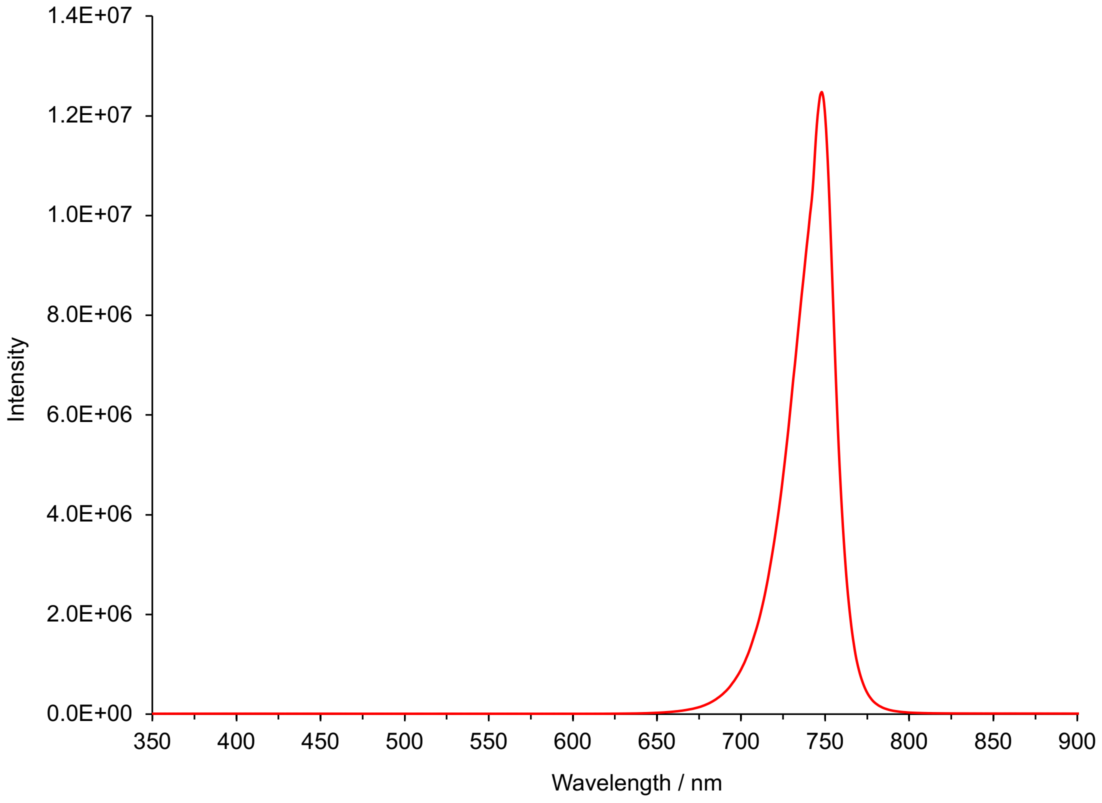

Figure S44. Emission spectrum of the 740 nm LED (Hepatochem) used in the photocatalytic experiments.

Electron-Transfer Photocatalytic Synthesis of 4-Methoxyphenol in Oxygenated Solution

A J-Young NMR tube was charged with 4-methoxybenzeneboronic acid (10 mg, 0.066 mmol), *N,N*,-diisopropylethylamine (23 μL, 0.13 mmol), [**Ir**]PF_6 (1.3 mg, 0.0013 mmol), and 0.6 mL of CD_3CN. Oxygen (O_2) was bubbled in for 10 min and then an NMR spectrum was collected (time zero). The NMR tube was then irradiated with 740 nm light and monitored by ^1H NMR spectroscopy at selected time points, with the final conversion measurements taken at 20 h (85%). This product was not isolated; conversion is reported based on ^1H NMR assignments verified by comparison to the literature.[2] ^1H NMR (500 MHz, CD_3CN, 22 °C): 6.74 (AB quartet, J_{HH} = 9.1 Hz, 4H), 3.70 ppm (s, 3H).

Energy Transfer Photocatalysis of Furfural in Oxygenated Alcoholic Solution

Furfural (8.6 μL, 0.10 mmol) and [**Ir**]PF_6 (1.0 mg, 0.0010 mmol) were added to a J-Young NMR tube and dissolved in 0.6 mL CD_3OD. O_2 was bubbled in for 10 min. A ^{1}H NMR spectrum was collected at time zero. The NMR tube was then irradiated with 740 nm light and monitored by ^{1}H NMR at selected time points. O_2 was re-bubbled in after 23 h of illumination, and a final NMR taken after an additional 3h of irradiation with 740 nm light to yield the final conversion (92%). This product was not isolated and conversion is reported based on ^{1}H NMR assignments verified by comparison to literature.[3] ^{1}H NMR (500 MHz, CD_3OD, 22 °C): 7.39 (d, J_{HH} = 5.7 Hz, 1H), 6.21 (d, J_{HH} = 5.8 Hz, 1H), 6.18 ppm (s, 3H).

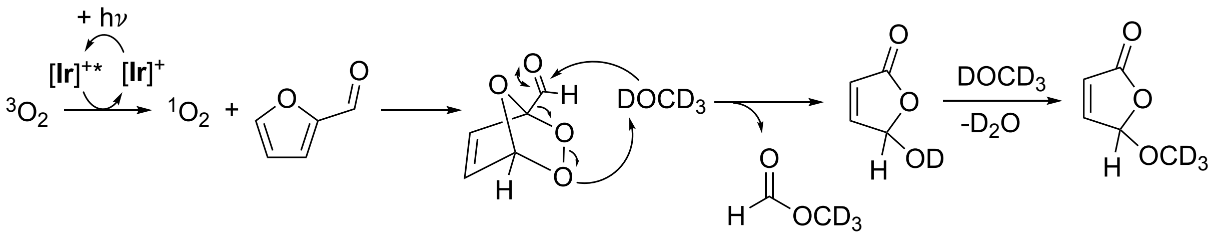

Figure S45. Proposed mechanism of furanone production from singlet oxygen-mediated reactivity of furfural in deuterated methanol.[21–24]

General Procedure for the Photocatalytic Oxidation of 10-Methyl-9-phenyl-9,10-dihydroacridine

In a glovebox, 10-methyl-9-phenyl-9,10-dihydroacridine (20 mg, 0.074 mmol), 2-bromo-4'-functionalized-acetophenone (0.22 mmol), and [**Ir**]PF_6 (1.5 mg, 0.0015 mmol) were added to a J-Young tube along with 0.5 mL CD_3CN. An NMR was taken at time zero and the tube was then irradiated with 740 nm light. The reaction monitored at the appropriate time points. Each reaction was stopped after full conversion was achieved. See above figures for analysis of NMR products.

Using 0.1 mol% of Photocatalyst for the Photocatalytic Oxidation of 10-Methyl-9-phenyl-9,10-dihydroacridine:

The procedure above was followed, with the following difference: 10 mL of a 2 x 10^{-4} M solution of [**Ir**]PF_6 was prepared in acetonitrile, and 0.37 mL of that solution transferred to a vial to isolate 7.4 x 10^{-5} mmol of the photocatalyst. This solution was then dried and dissolved in CD_3CN for use in the reaction.

Subsequent Additions of 10-Methyl-9-phenyl-9,10-dihydroacridine During Photocatalytic Reactions: The general procedure above was followed, however after 10 min of irradiation, full conversion, the NMR tube was brought back in the glovebox. In the glovebox, the solution in the tube was combined with another equivalent of 10-methyl-9-phenyl-9,10-dihydroacridine and irradiated with 740 nm light for another 10 min. This was repeated once more at which point all three equivalents of 2-bromoacetophenone were completely consumed.

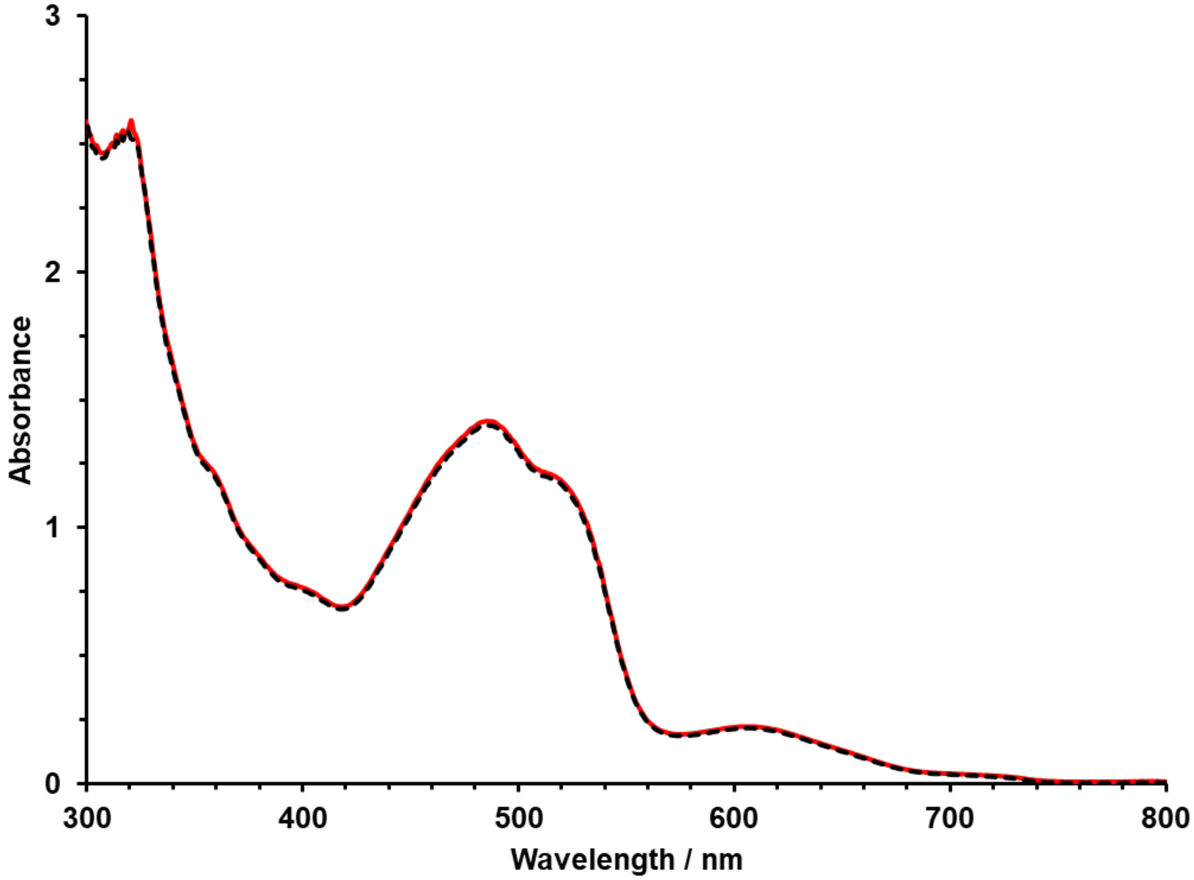

Figure S46. UV-Vis absorption spectra collected before and after ns oTA experiments (λ_{probe} = 540 nm) demonstrating the photostability of [**Ir**]PF_6.

References

(1) Nemez, D. B.; Ortiz, R. J.; Veilleux, K. A.; Williams, J. A. G.; Herbert, D. E. In Pursuit of Low Energy Phosphorescence: Late Metal Coordination Complexes of the Planar, π-Extended Bipyridyl Ligand 6,6′,7,7′-Biphenanthridine. *Chem.-Eur. J.* **2025**, *31*, e01802.

(2) Bürgin, T. H.; Glaser, F.; Wenger, O. S. Shedding Light on the Oxidizing Properties of Spin-Flip Excited States in a Cr^{III} Polypyridine Complex and Their Use in Photoredox Catalysis. *J. Am. Chem. Soc.* **2022**, *144*, 14181–14194.

(3) Ravetz, B. D.; Tay, N. E. S.; Joe, C. L.; Sezen-Edmonds, M.; Schmidt, M. A.; Tan, Y.; Janey, J. M.; Eastgate, M. D.; Rovis, T. Development of a Platform for Near-Infrared Photoredox Catalysis. *ACS Cent. Sci.* **2020**, *6*, 2053–2059.

(4) Krejčik, M.; Daněk, M.; Hartl, F. Simple Construction of an Infrared Optically Transparent Thin-Layer Electrochemical Cell: Applications to the Redox Reactions of Ferrocene, $Mn_2(CO)_{10}$ and $Mn(CO)_3$(3,5-Di-t-Butyl-Catecholate)$^-$. *J. Electroanal. Chem. Interf. Electrochem.* **1991**, *317*, 179–187.

(5) Neese, F. The ORCA Program System. *WIREs Comput. Mol. Sci.* **2012**, *2*, 73–78.

(6) Neese, F. Software Update: The ORCA Program System—Version 5.0. *WIREs Comput. Mol. Sci.* **2022**, *12*, e1606.

(7) Neese, F.; Wennmohs, F.; Becker, U.; Riplinger, C. The ORCA Quantum Chemistry Program Package. *J. Chem. Phys.* **2020**, *152*, 224108.

(8) Adamo, C.; Barone, V. Toward Reliable Density Functional Methods without Adjustable Parameters: The PBE0 Model. *J. Chem. Phys.* **1999**, *110*, 6158–6170.

(9) Ernzerhof, M.; Scuseria, G. E. Assessment of the Perdew–Burke–Ernzerhof Exchange-Correlation Functional. *J. Chem. Phys.* **1999**, *110*, 5029–5036.

(10) Wüllen, C. van. Molecular Density Functional Calculations in the Regular Relativistic Approximation: Method, Application to Coinage Metal Diatomics, Hydrides, Fluorides and Chlorides, and Comparison with First-Order Relativistic Calculations. *J. Chem. Phys.* **1998**, *109*, 392.

(11) Pantazis, D. A.; Chen, X.-Y.; Landis, C. R.; Neese, F. All-Electron Scalar Relativistic Basis Sets for Third-Row Transition Metal Atoms. *J. Chem. Theory Comput.* **2008**, *4*, 908–919.

(12) Marenich, A. V.; Cramer, C. J.; Truhlar, D. G. Universal Solvation Model Based on Solute Electron Density and on a Continuum Model of the Solvent Defined by the Bulk Dielectric Constant and Atomic Surface Tensions. *J. Phys. Chem. B* **2009**, *113*, 6378–6396.

(13) Neese, F.; Wennmohs, F.; Hansen, A.; Becker, U. Efficient, Approximate and Parallel Hartree–Fock and Hybrid DFT Calculations. A 'Chain-of-Spheres' Algorithm for the Hartree–Fock Exchange. *Chem. Phys.* **2009**, *356*, 98–109.

(14) Weigend, F. Accurate Coulomb-Fitting Basis Sets for H to Rn. *Phys. Chem. Chem. Phys.* **2006**, *8*, 1057–1065.

(15) Weigend, F.; Ahlrichs, R. Balanced Basis Sets of Split Valence, Triple Zeta Valence and Quadruple Zeta Valence Quality for H to Rn: Design and Assessment of Accuracy. *Phys. Chem. Chem. Phys.* **2005**, *7*, 3297–3305.

(16) Pantazis, D. A.; Neese, F. All-Electron Scalar Relativistic Basis Sets for the Lanthanides. *J. Chem. Theory Comput.* **2009**, *5*, 2229–2238.

(17) Pantazis, D. A.; Neese, F. All-Electron Scalar Relativistic Basis Sets for the 6p Elements. *Theor. Chem. Acc.* **2012**, *131*, 1292–1298.

(18) Pantazis, D. A.; Neese, F. All-Electron Scalar Relativistic Basis Sets for the Actinides. *J. Chem. Theory Comput.* **2011**, *7*, 677–684.
(19) Allouche, A.-R. Gabedit—A Graphical User Interface for Computational Chemistry Softwares. *J. Comput. Chem.* **2011**, *32*, 174–182.
(20) Lu, T.; Chen, F. Multiwfn: A Multifunctional Wavefunction Analyzer. *J. Comput. Chem.* **2012**, *33*, 580–592.
(21) Schenck, G. O. Photochemische Reaktionen II. Über Die Unsensibilisierte Und Photosensibilisierte Autoxydation von Furanen. *J. Lieb. Ann. Chem.* **1953**, *584*, 156–176.
(22) Grove, M. D.; Weisleder, D. Hydrolysis Products of 4-Acetamido-4-Hydroxy-2-Butenoic Acid .Gamma.-Lactone. *J. Org. Chem.* **1973**, *38*, 815–816.
(23) Heugebaert, T. S. A.; Stevens, C. V.; Kappe, C. O. Singlet-Oxygen Oxidation of 5-Hydroxymethylfurfural in Continuous Flow. *ChemSusChem* **2015**, *8*, 1648–1651.
(24) Ayoub, N.; Hamie, Z.; Toufaily, J.; Guénin, E.; Enderlin, G. Metal or Metal-Free Catalysis for the Oxidation of Biosourced Furfural. *ACS Sustainable Chem. Eng.* **2024**, *12*, 9568–9590.
